# Cuproptosis-related DNA methylation signature predict prognosis and immune microenvironment in cutaneous melanoma

**DOI:** 10.1007/s12672-024-01089-8

**Published:** 2024-06-14

**Authors:** Liucun Zhu, Xudong Kang, Shuting Zhu, Yanna Wang, Wenna Guo, Rui Zhu

**Affiliations:** 1 School of Life Sciences, Shanghai University, Shanghai, China; 2 School of Life Sciences, Zhengzhou University, Zhengzhou, China; 3 Department of Clinical Laboratory Medicine, Shanghai Tenth People’s Hospital of Tongji University, Shanghai, China

**Keywords:** Cuproptosis, DNA methylation, Prognosis, Immune, Cutaneous melanoma

## Abstract

The prognosis for Cutaneous Melanoma (CM), a skin malignant tumor that is extremely aggressive, is not good. A recently identified type of controlled cell death that is intimately related to immunotherapy and the development of cancer is called cuproptosis. Using The Cancer Genome Atlas (TCGA) and the Gene Expression Omnibus (GEO) database, we developed and validated a DNA-methylation located in cuproptosis death-related gene prognostic signature (CRG-located DNA-methylation prognostic signature) to predict CM’s prognosis. Kaplan–Meier analysis of our TCGA and GEO cohorts showed that high-risk patients had a shorter overall survival. The area under the curve (AUC) for the TCGA cohort was 0.742, while for the GEO cohort it was 0.733, according to the receiver operating characteristic (ROC) analysis. Furthermore, this signature was discovered as an independent prognostic indicator over CM patients based on Cox-regression analysis. Immunogenomic profiling indicated that majority immune-checkpoints got an opposite relationship with the signature, and patients in the group at low risk got higher immunophenoscore. Several immune pathways were enriched, according to functional enrichment analysis. In conclusion, a prognostic methylation signature for CM patients was established and confirmed. Because of its close relationship to the immune landscape, this signature may help clinicians make more accurate and individualized choices regarding therapy.

**Supplementary Information:**

The online version contains supplementary material available at 10.1007/s12672-024-01089-8.

## Introduction

Cutaneous Melanoma (CM) is a malignant tumor originating from melanocytes, which are capable of producing melanin. It can metastasize to multiple organs and is one of the most invasive and drug-resistant cancers. Global cancer statistics for 2020 revealed 324,635 new cases of CM, which caused 57,043 deaths [1]. In recent decades, both the number of cases and death rate of CM have increased [1]. There are various treatment options for CM. Early stage localized regional CM can be treated by surgical removal, but the mortality rate remains high and the outcome is poor for CM that has spread and metastasized [2, 3]. Additionally, only 27% of patients with metastatic CM survive chemotherapy for 5 years [4]. The prognosis for patients with CM is still poor, despite improvements in survival rates due to the advancement of immunotherapy and targeted therapy [5]. In recent years, new immune checkpoint blockades combinations have significantly improved the outcome of patients with metastatic melanoma. However, tumor resistance to ICBs has hindered the progress of its clinical application [6, 7]. For example, the response rate for melanoma patients treated with pembrolizumab (anti-PD-1) was only 33% [8]. About half of the patients do not show long-lasting benefit with ICBs [9]. Therefore, the discovery and validation of a new molecular marker for more definitive prognostic assessment and individualized treatment of CM patients is urgently needed.

Copper is an important metal element that is necessary for life, and maintaining normal physiological processes and internal balance depends frequently on copper levels [10]. The human body uses several kinds of Cu chaperones and Cu transport proteins to transport copper [11]. Using the cellular utilization of copper ions as an example, Cu2+ is reduced to Cu+ on the cell surface of the gastrointestinal tract by metalloreductases [12]. Subsequently, CTR1 carries Cu+ into the intestinal epithelial cells [13]. Cu+ is then released into the portal vein by ATP7A [14]. Most Cu+ is delivered into liver and stored by binding to albumin, trans copper protein, and other plasma proteins [15]. When there is an excess of Cu+ in the hepatocytes, it is transported into the bile in the form of vesicles via Cu transporting ATPase B (ATP7B) and excreted through the bile duct [16].

A recently identified type of cell death who necessitates the metabolism of mitochondria is called cuproptosis [17]. Intracellular copper ion buildup may be involved in the lipoy-lation and aggregation of enzymes governed by the mitochondrial TCA. This instability of the associated iron-sulfur cluster proteins can subsequently result in proteotoxic stress and, in the event of a fatality, cell death [17].

Numerous investigations indicate the correlation between copper metabolism and tumorigenesis, and cuproptosis death-related genes (CRGs) have also been found to potentially influence tumor progression and serve as potential prognostic indicators [18, 19]. In order to identify appropriate prognostic indicators for CM, we became interested in the function of CRGs in the progression and prognosis of the disease.

DNA methylation is usually thought of as a gene-silencing mechanism and is crucial for numerous biological processes, including embryogenesis, transcription, genomic imprinting [20]. Meanwhile, DNA methylation patterns are frequently altered in cancer, and aberrant promoter methylation is regulated at almost every step of tumorigenesis [21]. Because abnormal DNA methylation status usually occurs in the initial phases of cancer, and because methylated DNA is more stable than RNA and proteins, the use of methylation as a signature has better stability and reproducibility [22]. Therefore, early cancer detection and prognosis has benefited greatly from the widespread use of DNA methylation signatures [23].

In this study, we investigated the significance of DNA methylation sites located on CRGs in the prognostic profile and tumor microenvironment (TME) of CM. Using DNA methylation levels, gene expression and clinical data of CM, a combination of methylation signatures was established. After validation and independence analysis, as well as functional enrichment analysis, it was found to be associated with immune infiltration of tumor cells and immune checkpoints, revealing its possible mechanism of action. Our research provides valuable insights into the prognostic characteristics of CM and identifies a validated predictive tool that can be used for clinical prediction of CM patient survival.

## Results

### Screening and establishment of prognosis-related CRG DNA methylation signature

A total of 96 CRGs were collected and 1374 DNA methylation sites located in CRGs were extracted. Epigenetic modifications have been demonstrated to affect RNA content and in turn plays a key role in the development of tumour. Therefore, we calculated the spearman correlation coefficient between DNA methylation sites and corresponding genes, and screened 695 methylation sites corresponding to and significantly associated with CRGs (*p* < 0.05). These DNA methylation sites were used in a multivariate Cox regression analysis to construct all possible models of 1–5 sites, a CRG DNA methylation signature was selected as the best prognostic model for predicting survival through ROC analyses. The *p* value of the likelihood ratio test was 4e−09 and the *p* value of the cox proportional hazards hypothesis test was 0.066 for the model. The final model for predicting OS consisted of five DNA methylation sites (cg00668852, cg01130192, cg18645035, cg22134611, cg25522181). The genes corresponding to these five sites are AOC1 (Amine oxidase copper-containing 1), ACO1 (Aconitase 1), DPYD (Dihydropyrimidine Dehydrogenas), ABCB8 (ATP Binding Cassette Subfamily B Member 8), and CIAO1 (Cytosolic Iron-Sulfur Assembly Component 1). Relevant information about these sites is shown in Table 1.

**Table 1:** Information of five CRG-located DNA-methylation sites

Probe ID	Probe location	Gene symbol	Coefficient	HR	95% CI
cg00668852	chr7:150857025–150857026	AOC1	1.13603	3.112	1.487–6.516
cg01130192	chr9:32385004–32385005	ACO1	− 3.86891	0.021	0.002–0.228
cg18645035	chr1:97094252–97094253	DPYD	− 1.1115	0.329	0.193–0.561
cg22134611	chr7:151041217–151041218	ABCB8	− 1.59234	0.203	0.021–1.996
cg25522181	chr2:96267545–96267546	CIAO1	1.4507	4.264	1.584–11.477

### Evaluation of the CRG DNA methylation prognosis signature

The risk scores of CM patients were calculated according to the model: ${\text{riskScore}} = \left( {1.13603 \times\upbeta \;{\text{value}}\;{\text{of}}\;{\text{cg}}00668852} \right) + \left( { - 3.86891 \times \upbeta \;{\text{value}}\;{\text{of}}\;{\text{cg}}01130192} \right)$ $+ \left( { - 1.1115 \times \upbeta \;{\text{value}}\;{\text{of}}\;{\text{cg}}18645035} \right) + \left( { - 1.59234 \times \upbeta \;{\text{value}}\;{\text{of}}\;{\text{cg}}22134611 } \right)$ $+ \left( {1.4507 \times\upbeta \;{\text{value}}\;{\text{of}}\;{\text{cg}}25522181} \right).$ CM patients were separated into high- and low-risk groups based on the median risk score.

**Fig. 1:**
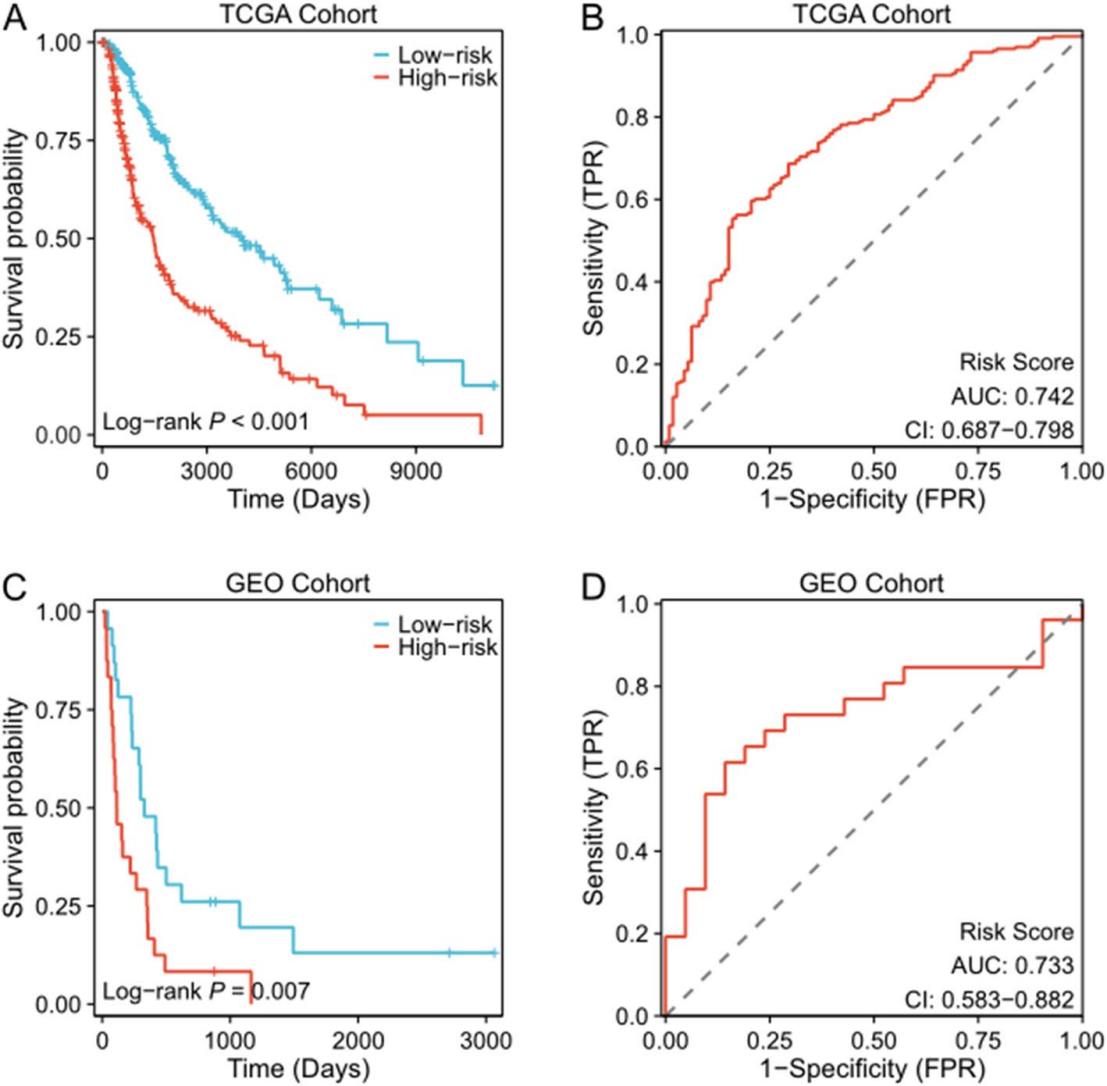
Identification of CRG DNA methylation prognosis signature in CM patients. **A**, **C** Kaplan–Meier curves for high-risk and low-risk groups in TCGA cohort and GEO cohort. **B**, **D** ROC curves of CRG DNA methylation prognosis signature in TCGA cohort and GEO cohort

In the TCGA cohort, Kaplan–Meier survival analysis revealed a significantly lower survival rate in the high-risk group (*p* < 0.001, Fig. 1A). The receiver operating characteristic (ROC) curve analysis exhibited that the signature had a good predictive accuracy with an area under the curve (AUC) value of 0.742 and a Confidence interval (CI) of 0.687–0.798 (Fig. 1B).

To further explore the prognostic value of this signature, we discovered in the independent GEO cohort that patients with a high-risk score had a significantly worse survival prognosis (*p* = 0.007, Fig. 1C), with an AUC of 0.733 (Fig. 1D). This finding validates the excellent predictive performance of this prognostic signature for CM patients.

In addition, univariate Cox regression analysis, Kaplan–Meier and ROC analyses were performed on the five DNA methylation sites in TCGA (Figure S1) and GEO (Figure S2) cohorts to further investigate their prognostic significance in CM. Kaplan–Meier analysis revealed that individual DNA methylation sites could also differentiate high-risk from low-risk patients. ROC analysis indicated that the prognostic performance of DNA methylation site combinations was superior to that of individual DNA methylation sites alone. These findings suggest that DNA methylation site combinations can provide a better prognostic prediction for CM patients.

### Correlation between CRG DNA methylation signature and clinical characteristics

**Fig. 2:**
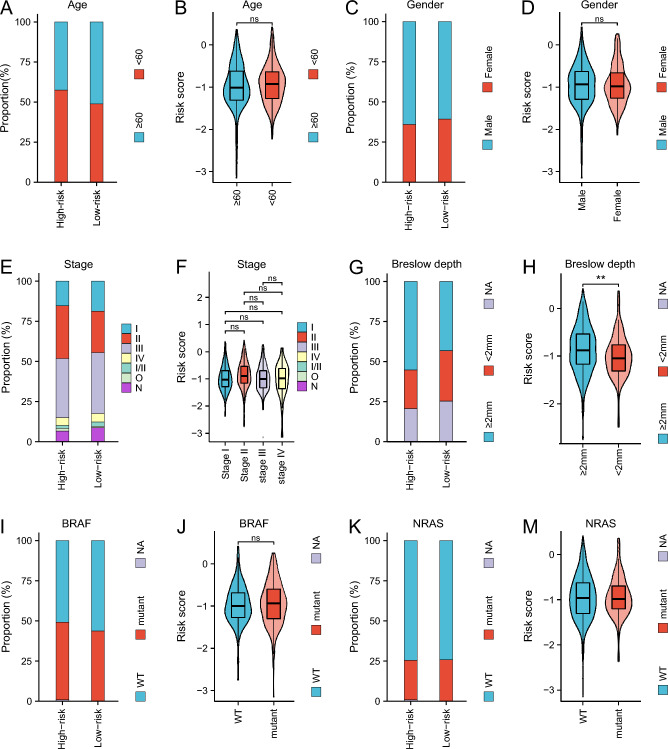
Distribution of risk ratios and scores for patients with different age (**A**, **B**), gender (**C**, **D**), tumor stage (**E**, **F**), Breslow depth (**G**, **H**), BRAF (**I**, **J**) and NRAS (**K**–**M**) mutation in the TCGA cohort. **p < 0.01, *p < 0.05, ns: no significance

To assess the link between the signature and clinical characteristics, we further analyzed the patients stratified by different characteristics and investigated whether characteristics could differentiate patients’ survival in the TCGA cohort. The findings show that the two patient groups’ age (Fig. 2A, B), gender (Fig. 2C, D) and tumor stage (Fig. 2E, F) did not significantly differ from one another, suggesting that the signature was not influenced by these clinical factors and was independent in guiding the prognosis of patients. However, Breslow depth exhibited significant differences, reflecting the consistency between risk scores and Breslow depth (Fig. 2G, H). The pathogenic variants of the BRAF and NRAS are important for CM. So, we assessed the alterations in NRAS and BRAF within the groups with the highest and lowest risk. The analysis revealed no significantly different results between two groups, indicating that BRAF mutation (BRAF p.V600, Fig. 2I, J) and NRAS mutation (NRAS p.Q61, Fig. 2K–M) likewise did not affect the prognostic effect of the signature.

### Analysis of independence and nomogram construction

**Fig. 3:**
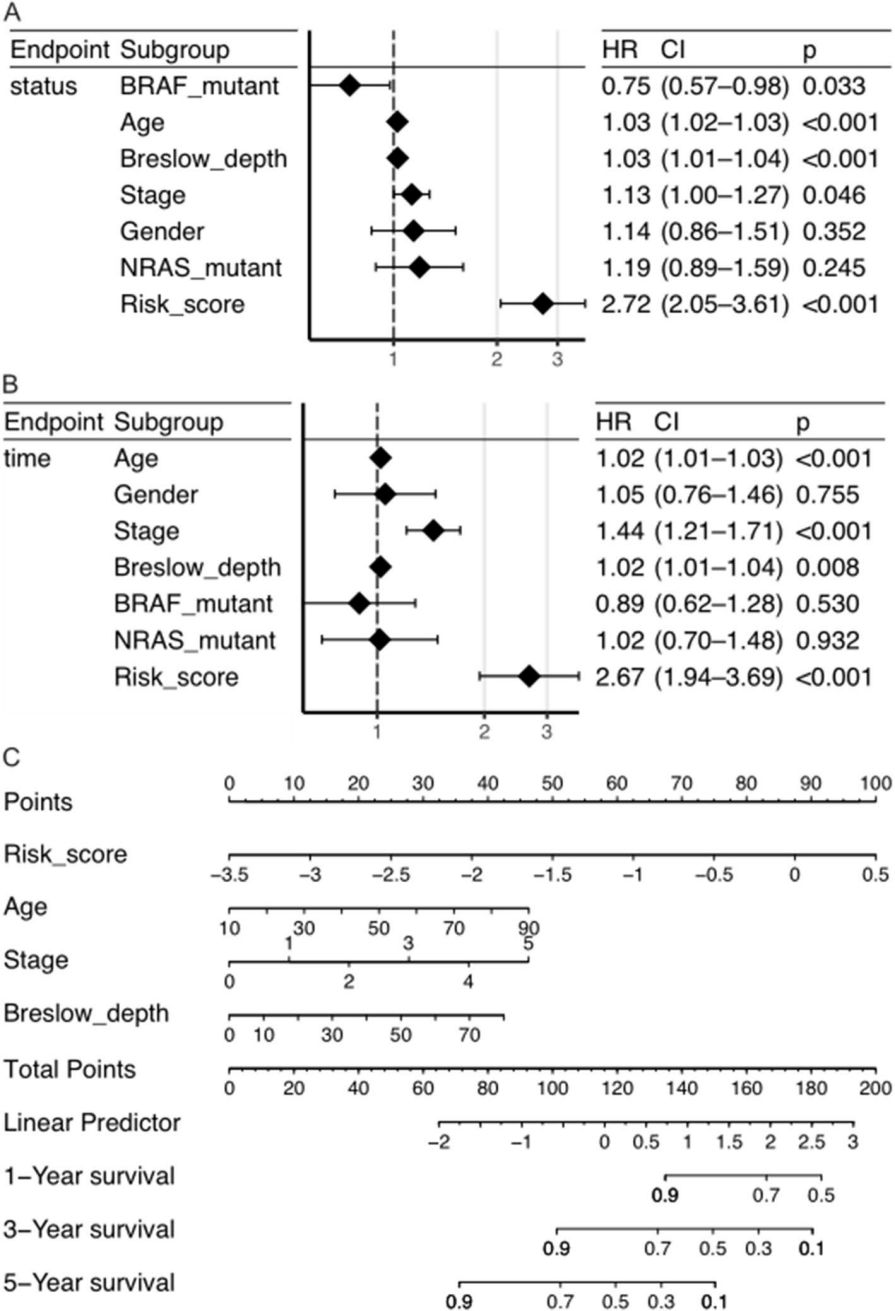
Univariate- (**A**) and multivariate-Cox-regression (**B**) of characteristics (age, gender, stage, and Breslow depth, BRAF- and NRAS-mutant, risk score) in the TCGA cohort. **C** The nomogram of characteristics in the TCGA cohort

We performed Cox regression to examine the signature’s independence from additional clinical characteristics (age, breslow depth, gender, tumor stage, and BRAF and NRAS mutations) in the TCGA cohort (Fig. 3A, B). The findings indicated that the signature could be used as an independent prognostic indicator because risk score, age, stage, and Breslow depth were all strongly related to overall survival (OS) in univariate-Cox-regression and remained so in multivariate-Cox -regression. Next, we used age, stage, breslow depth, and risk score to create nomograms for OS (Fig. 3C). Each predictor with a given value can be mapped to the Points axis, and the sum of these points can be referred to in the Total Points axis. Then the probability of 1-, 3- and 5-year survival for patient can be obtained from corresponding axis. The ROC curve analysis exhibited that the nomograms had a good predictive accuracy, and the AUC value of 1-, 3- and 5-year were 0.86, 0.84 and 0.80, respectively (Figure S3), indicating that the OS prognostic nomograms can guide the clinical management of patient.

### Assessment regarding the CRG DNA methylation signature grouping by clinical characteristics

Different tumor locations can impact patient prognosis (Table 2). For the primary tumor site, Kaplan–Meier survival analysis demonstrates that there is no significant difference in survival between the high- and low-risk groups in the TCGA cohort (*p* = 0.165, Figure S4A). ROC curve analysis indicates that the predictive model holds certain prognostic value, with an AUC value of 0.725 (Figure S4B). For the remaining clinical characteristics included Distant Metastasis, Regional Cutaneous or Subcutaneous, and Regional Lymph, Kaplan–Meier survival analyses showed that patients in the high-risk group all had significantly lower survival rates than those in the low-risk group (Figure S4C-H). The signature demonstrates high sensitivity and specificity in predicting OS associated with these clinical characteristics, with AUC values exceeding 0.72 for all.

**Table 2:** Kaplan–Meier and ROC results of different regrouping

Regrouping factors	Group	Kaplan–Meier *p* value	AUC	95% CI of AUC
Tumor location	Primary tumor	0.165	0.725	0.421–1.000
	Distant metastasis	0.007	0.73	0.566–0.894
	Regional cutaneous or subcutaneous tissue	< 0.001	0.753	0.607–0.899
	Regional lymph node	< 0.001	0.72	0.639–0.800
Ulceration	No	0.106	0.703	0.595–0.812
	Yes	0.002	0.73	0.632–0.828
Radiation therapy	No	< 0.001	0.818	0.738–0.898
	Yes	0.096	0.848	0.686–1.000
Immunotherapy	No	< 0.001	0.727	0.665–0.788
	Yes	0.003	0.846	0.729–0.962
Chemotherapy	No	< 0.001	0.723	0.661–0.784
	Yes	0.003	0.822	0.701–0.944

Different ulceration indicators also effects on the prognosis of patients (Table 2). For patients without ulceration (ulceration NO) in the TCGA cohort, Kaplan–Meier survival analysis demonstrates that there is no significant difference in survival between the high- and low-risk groups (*p* = 0.106, Figure S5A). ROC curve analysis reveals that the predictive model has some predictive value with an AUC of 0.703 (Figure S5B). For CM patients with ulceration (ulceration YES), Kaplan–Meier survival analysis indicated that patients in the high-risk group had significantly lower OS than those in the low-risk group (*p* = 0.002, Figure S5C). ROC curve analysis suggested that the AUC values are both greater than 0.7. This suggests that for melanoma patients with ulceration, the predictive accuracy is 0.730 (Figure S5D).

Some patients with CM receive different therapy in the TCGA cohort (Table 2). For these patients who did undergo radiation therapy (Radiation Therapy YES), Kaplan–Meier survival analysis showed that there was no significant difference between the high- and the low-risk group (*p* = 0.096, Figure S6A). ROC curve analysis indicated that this predictive model has some predictive value, with an AUC of 0.848 (Figure S6B). For patients who did not undergo radiation therapy (Radiation Therapy NO), shorter OS for high-risk patients compared to low-risk patients (*p* < 0.001, Figure S6C) and the AUC of the ROC curve was 0.818 (Figure S6D). In addition, Kaplan–Meier survival analysis showed significant differences between high- and low-risk groups for both patients with immunotherapy (Immunotherapy YES, *p* = 0.003) and non-immunotherapy (Immunotherapy NO, *p* < 0.001), with AUC of 0.846 and 0.727 (Figure S7). Similarly, there were significant differences between high- and low-risk groups of chemotherapy patients (Chemotherapy YES, *p* = 0.003) and non-chemotherapy patients (chemotherapy NO, *p* < 0.001), with AUC of 0.822 and 0.723, respectively (Figure S8). These results suggest that this signature still has high accuracy in predicting OS after regrouping by the above clinical characteristics.

### Association of CRG DNA methylation prognostic signature with immune landscape

**Fig. 4:**
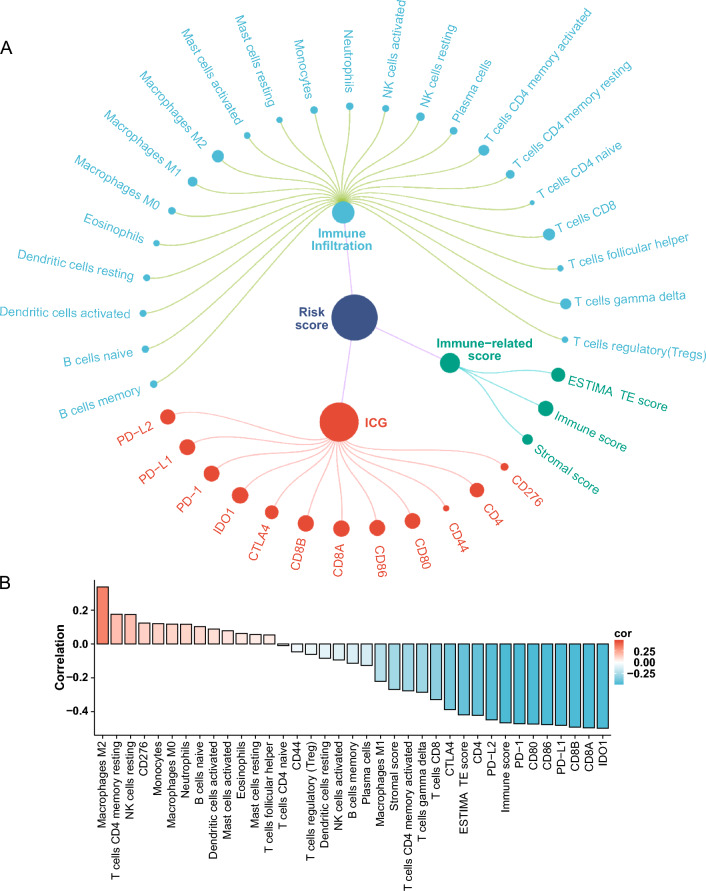
Correlation of CRG DNA methylation signature with 22 types of immune cell infiltration, stromal scores (the level of stromal cells present in tumor tissue), immune scores (the infiltration level of immune cells in tumor tissues), ESTIMATE scores (tumor purity), and ICGs in the TCGA cohort. **A** Correlation *p* value of CRG DNA methylation characteristics with 22 kinds of immune cell infiltration, immune-related scores and ICGs. The size of the dot represents the *p* value. The larger the dot, the smaller the p value. The color of the dot represents the category, blue represents immune cell infiltration, green represents immune-related scores, and red represents ICGs. **B** Correlation coefficient of CRG DNA methylation characteristics with 22 kinds of immune cell infiltration, immune-related scores and ICGs

**Fig. 5:**
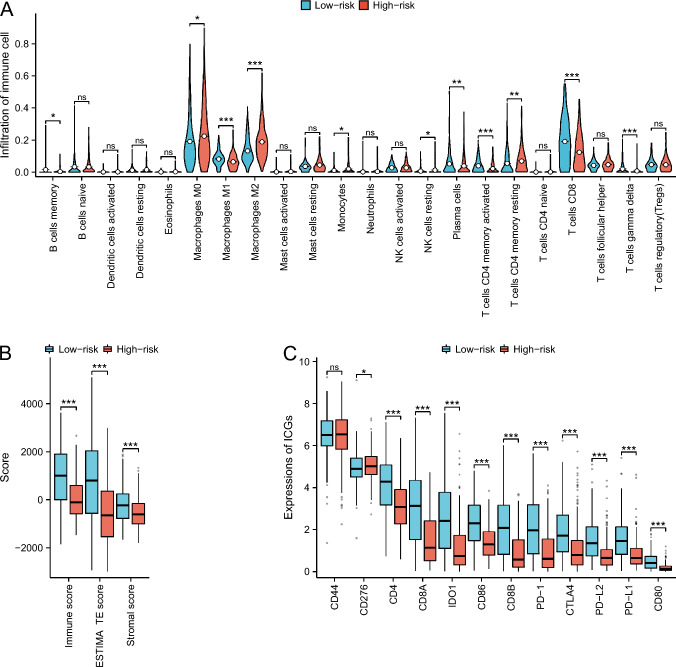
The differences in 22 types of immune infiltration (**A**), stromal scores (the level of stromal cells present in tumor tissue), immune scores (the infiltration level of immune cells in tumor tissues), ESTIMATE scores (tumor purity) (**B**), and expression of ICGs (**C**) between high- and low-risk CM patients in the TCGA cohort

To further understand the potential relationship between the signature and the immune landscape of CM patients, we analyzed the correlation of CRG DNA methylation signature with immune and compared differences in immune cell infiltration among the high- and low-risk groups in the TCGA cohort. The results indicated a negative correlation between risk scores and ESTIMATE scores (tumor purity), immune scores (the infiltration level of immune cells in tumor tissues), and stromal scores (the level of stromal cells present in tumor tissue) (Fig. 4). This result suggests that the lower the three scores, the higher the patient risk. The risk scores were negatively correlated with Plasma cells, T cells CD8, T cells CD4 memory activated, T cells gamma delta, B cells memory and macrophages M1, while positively correlated with Neutrophils, Monocytes, NK cells resting, Macrophages M0, Macrophages M2, and T cells CD4 memory resting (Fig. 4). In addition, the immune cell infiltration level of B cells memory, Macrophages M0, Macrophages M1, Macrophages M2, Monocytes, NK cells resting, Plasma cells, T cells CD4 memory activated, T cells CD4 memory resting, T cells CD8,T cells gamma delta have significant differences between high- and low-risk groups (Fig. 5A). Meanwhile, the analysis confirmed differential ESTIMATE scores, immune scores, and stromal scores between the high- and low-risk groups, with all three scores significantly increased in the low-risk group (Fig. 5B). To investigate tissue-specific and the impact of immunotherapy, we explored immune infiltration with primary tumor, metastatic, immunotherapy, and non-immunotherapy patients. The ESTIMATE scores, immune scores, the immune cell infiltration level of T cells CD4 memory activated, T cells CD4 memory resting, T cells CD8, Macrophages M0, Macrophages M2, and Neutrophils have significant differences between high- and low-risk groups in primary tumor patients (Figure S9). The ESTIMATE scores, immune scores, stromal scores, the immune cell infiltration level of B cells naive, T cells CD4 memory activated, T cells CD4 memory resting, T cells CD8, Plasma cells, T cells gamma delta, Macrophages M1, Macrophages M2, Monocytes, NK cells resting, and Plasma cells have significant differences between two risk groups in metastatic patients (Figure S10). On the non-immunotherapy patients, the ESTIMATE scores, immune scores, stromal scores, the immune cell infiltration level of B cells memory, Macrophages M0, Macrophages M1, Macrophages M2, Monocytes, NK cells resting, Plasma cells, T cells CD4 memory activated, T cells CD4 memory resting, T cells CD8, and T cells gamma delta have significant differences between different groups (Figure S11). On patients with immunotherapy, only immune scores, the immune cell infiltration level of T cells CD4 memory activated and T cells gamma delta have significant differences (Figure S12). Considering the significance of immune checkpoint-associated genes (ICGs) in clinical treatment, we further compared the expression differences of ICGs between the high- and low-risk groups. The ICGs included CD44, CD276, CD4, CD8A, IDO1, CD86, PD-1, CTLA4, PD-L2, PD-L1, and CD80. The results indicated that there was a direct correlation between the risk scores and ICGs expression (Fig. 4). ICGs expression was significantly different between the high- and low-risk group (Fig. 5C).

### Functional enrichment analyses

**Fig. 6:**
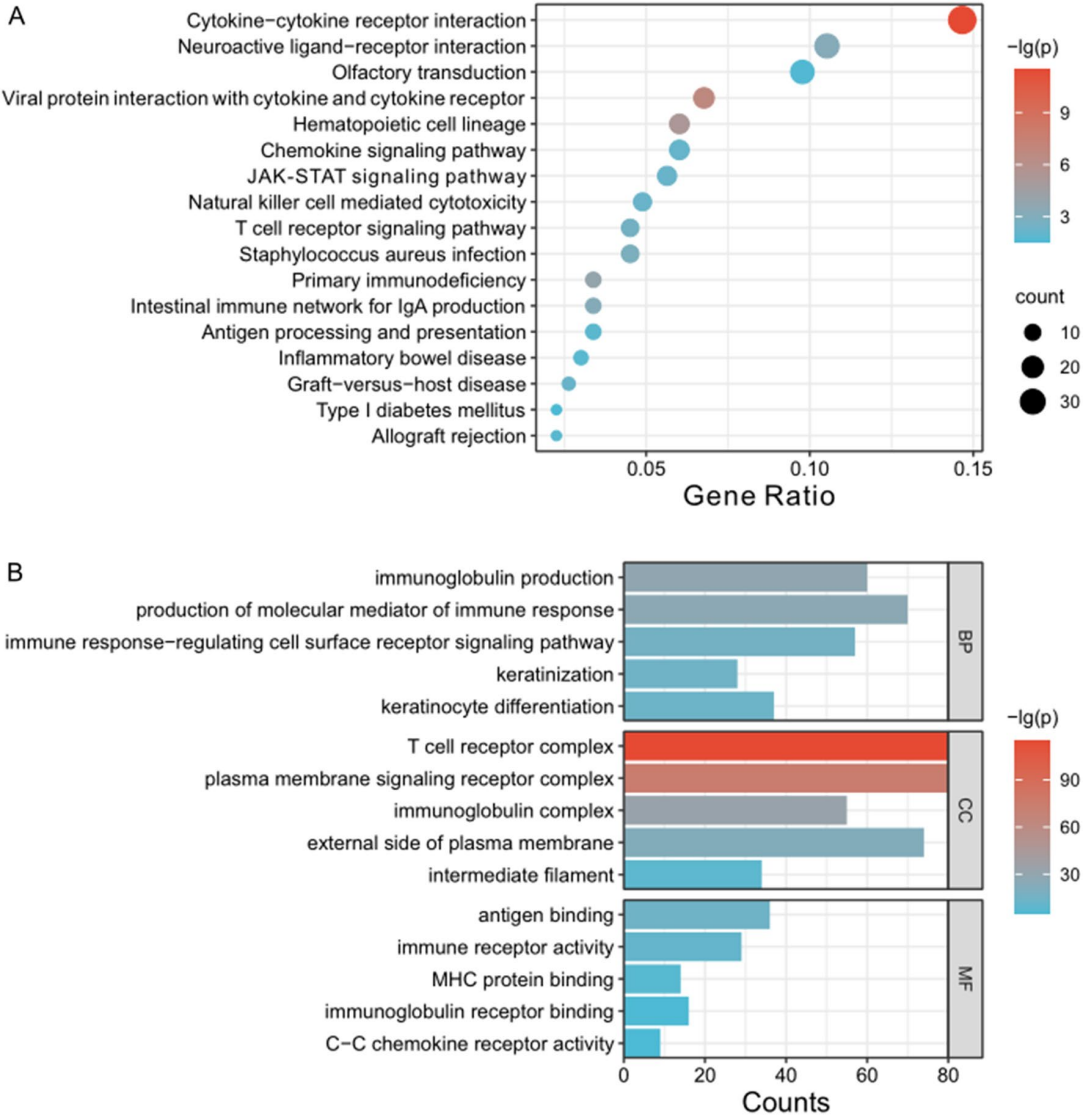
The KEGG enrichment analysis (**A**) and GO enrichment analysis (**B**) of DEGs in high- and low-risk groups in the TCGA cohort

To investigate the potential mechanisms underlying different prognosis risk groups, we identified differentially expressed genes (DEGs) between the high- and low-risk groups in the TCGA cohort (Table S1). Thereafter, Gene Ontology (GO) and Kyoto Encyclopedia of Genes and Genomes (KEGG) analyses were performed on these DEGs. KEGG enrichment analysis revealed significant enrichment in the cytokine-cytokine receptor interaction, viral protein interaction with cytokine and cytokine receptor, hematopoietic cell lineage, neuroactive ligand-receptor interaction, etc. (Fig. 6A, Table S2). GO functional enrichment analysis results indicated that the DEGs were mainly enriched in T-cell receptor complex, plasma membrane signaling receptor complex, and production of molecular mediator of immune response (Fig. 6B, Table S3). The above results suggest that the risk signature potentially contributes to the formation of immune microenvironment in CM patients.

Furthermore, we performed GSVA analyses to explore the differences in enriched signaling pathways between the high- and low-risk groups (Figure S13). The results showed that significant differences in natural killer cell mediated cytotoxicity, toll like receptor signaling pathway, T cell receptor signaling pathway, JAK stat signaling pathway and oxidative phosphorylation between the two risk groups. At the same time, there are differences in GO processes such as interferon gamma mediated signaling pathway, regulation of NK T cell activation, regulation of response to tumor cell, immune response to tumor cell, regulation of response to tumor cell, immune response to tumor cell, positive regulation of natural killer cell activation.

### Association of CRG DNA methylation prognostic signature with gene expression

To examine the tissue characteristics of DNA methylation sites, we analyzed the differences in methylation sites between patients with metastatic and primary tissues in the TCGA cohort. Our analysis revealed the methylation levels of cg01130192 and cg18645035 were significantly higher in the metastatic tissues. However, the regression coefficient of cg01130192 and cg18645035 in the CRG DNA methylation prognostic signature is negative, indicating that they are negatively correlated with patients’ survival risk. The higher the methylation level of sites, the lower the survival risk. These results suggest that the metastasis and primary tumor are not necessarily consistent with the survival risk of patients. In contrast, the methylation levels of cg00668852, cg22134611, and cg25522181 did not differ between the two tissue types (Figure S14).

To investigate the relationship between DNA methylation sites and gene expression, we conducted Spearman correlation analyses between DNA methylation sites and their corresponding gene expressions. Our findings showed that the methylation levels of cg00668852 and cg18645035 were significantly positively correlated with the expression of their respective genes. In addition, we observed a significant negative correlation between the methylation levels of cg22134611, cg25522181, and cg01130192 and their corresponding gene expressions (Figure S15).

## Discussion

CM is one of the deadliest types of skin tumors, often rapidly progressing and infiltrating lymph nodes and other organs [21, 22]. Consequently, the lack of precise and effective prognostic tools has remained a critical weakness in CM treatment [23]. Copper, an essential element involved in cellular proliferation and apoptosis, serves as a crucial cofactor for enzymes and transport proteins [24]. Recent research indicates a significant increase in copper levels within malignant tumors, where cuproptosis plays a pivotal role in cancer progression [14]. Predictive signature based on CRG DNA methylation sites may contribute to enhancing the survival rates of CM patients. Therefore, in this study, we constructed and validated DNA methylation site signature associated with cuproptosis in CM.

DNA methylation is an epigenetic modification that can regulate gene expression without altering the DNA sequence [25, 26]. Abnormal DNA methylation is associated with the development of various malignant tumors, such as gastric cancer, liver cancer, lung cancer, breast cancer, prostate cancer [27–29]. The role of epigenetic modifications in tumorigenesis primarily involves two aspects. First, it promotes tumorigenesis and progression by altering the epigenetic status of tumor cells themselves, leading to the silencing of tumor suppressor genes or the activation of oncogenes [30, 31]. Second, it regulates the immune response to tumors by altering the epigenetic status of immune and mesenchymal stromal cells in the tumor microenvironment, affecting immune surveillance and immune escape [32, 33].

Our research found that the methylation levels of cg00668852 and cg18645035 were significantly positively correlated with their gene expression, and the methylation levels of cg22134611, cg25522181 and cg01130192 were significantly negatively correlated with their gene expression. Several studies have also highlighted that the corresponding genes of these five DNA methylation sites may play an important role in cancer development. AOC1 is a copper-containing diamine oxidase that catalyzes the oxidative degradation of histamine and other polyamines [34]. AOC1 also activates the AKT signaling pathway and regulates the epithelial-to-mesenchymal transition (EMT) process, which promotes tumor cell proliferation and migration [35]. ACO1 is a gene that encodes a mitochondrial iron-sulfur protein involved in the citric acid cycle and iron metabolism [36, 37]. ACO1 can also further promote or inhibit cuproptosis by influencing the stability of iron-sulfur cluster proteins. DPYD is a lipidated tricarboxylic acid cycle protein that catalyzes the conversion of uracil to uridine, thus participating in uracil metabolism. As a lipidated tricarboxylic acid cycle protein, DPYD is also one of the targets of cuproptosis. Consequently, intracellular oxidative stress and DNA damage were inducted, ultimately facilitating the process of cuproptosis. ABCB8 and CIAO1 are mitochondrial membrane proteins that can participate in mitochondrial iron metabolism and iron-sulfur cluster synthesis [38–40]. ABCB8 can bind to mRNAs containing iron-regulatory elements (IREs) in the nucleus, resulting in the regulation of the expression of iron-related genes [41]. CIAO1 also affects tolerance and toxicity of fluoropyrimidines in tumor patients.

Furthermore, several studies have shown that the expression of AOC1 is significantly negatively correlates with the expression of CRGs [42]. The downregulation of AOC1 may lead to intracellular copper accumulation, which induces cuproptosis. The expression levels of ABCB8 and ACO1 in CM were negatively correlated with the prognostic risk of the tumor. The lower the expression level, the higher the risk. The downregulation of ABCB8 and ACO1 can enhance CM cell proliferation, migration and invasion capabilities, thus promoting the development of tumor. On the other hand, the expression levels of CIAO1 and DPYD in CM have demonstrated a positive correlation with tumor prognostic risk.

Various components of the immune system play an important role in the occurrence and development of tumors, and tumor immunotherapy has received significant attention and development. In our study, the expression of some immune checkpoint genes was strongly correlated with risk scores, suggesting that the state of tumor immune microenvironment may be influenced by CRG DNA methylation prognostic signature. Immunotherapy is a relatively important part of the CM treatment strategy, but about half of patients do not achieve long-term benefits, and adverse reactions often occur. The CRG DNA methylation prognostic signature demonstrated high predictive accuracy for OS across various therapies. Kaplan–Meier analysis confirmed prolonged OS in the low-risk group for both patients with/without immunotherapy or chemotherapy. Nevertheless, there is a lack of data to explore the performance of CRG DNA methylation prognostic signature in clinical practice, so further studies is needed in the future to understand whether CM patients stratified by the signature might specifically benefit from immunotherapy. It is expected that the CRG DNA methylation prognostic signature proposed in this study could accurately stratify the survival risk of patients, reduce the abuse of anticancer drugs, achieve individual precision immunotherapy, and in turn improve the quality of treatment.

Epigenetic modifications could affect RNA content and plays a key role in the development of tumour. In our study, we found that the methylation levels of the sites were significantly correlated with their corresponding gene expression, and these corresponding genes were closely related to the occurrence and development of tumors, and some studies have explored the mechanism of these genes. This suggests that the CRG DNA methylation signature may alter tumor status by influencing the expression of corresponding genes. Using our model to stratify patients’ risk, we found that the DEGs between high- and low-risk groups were significantly enriched in the cytokine-cytokine receptor interaction, T-cell receptor complex, and production of molecular mediator of immune response. The GSVA analyse results also showed that significant differences in natural killer cell mediated cytotoxicity, T cell receptor signaling pathway, JAK stat signaling pathway, regulation of NK T cell activation and immune response to tumor cell between the two risk groups. These results suggest that the risk signature potentially contributes to the formation of immune microenvironment in CM patients. At present, the CRG DNA methylation signature has potential application value in clinical diagnosis, prognosis assessment and personalized treatment, but there is no direct evidence to clarify its physiological significance. In the future, we will further explore the mechanism and physiological significance.

## Materials and methods

### Dataset source and pre-processing

We collected DNA methylation data, transcriptome profiles, and clinical data from The Cancer Genome Atlas (TCGA) databases (TCGA-SKCM, N = 457) as the training cohort. The patient's clinical information is shown in Table S4. Data from the Gene Expression Omnibus (GEO) databases (GSE51547, N = 47) were used as the validation cohort. Only samples with both survival status and time information were used. DNA methylation levels were represented as β values ranging from 0 (no methylation) to 1 (100% methylation), and only use samples that have both survival and temporal information. CRGs were obtained from literature [42]. The technical roadmap is detailed in Figure S16.

### Prognosis-related DNA methylation filtering and risk model construction

Univariate Cox regression analysis was performed using the R “survival” package to identify DNA methylation sites associated with overall survival (OS) from clinical information of CM patients and DNA methylation level of the site. Statistically significant sites were then selected for multivariate Cox regression analysis. Models containing all possible combinations of two to five sites were constructed and further screened for DNA methylation signatures associated with OS. Using the DNA methylation levels and the corresponding regression coefficients, a prognostic risk score could be calculated for each CM patient. Patients were then stratified into high- and low-risk groups based on their risk scores.

### DNA methylation prognostic signature validation

To assess the utility of prognostic signatures, Kaplan–Meier survival curves and Wilcoxon rank test were employed to compare the differences in prognosis between high- and low-risk groups [43]. The receiver operating characteristic (ROC) curves were utilized to calculate the sensitivity and specificity of the signatures, estimating their predictive capability for CM patient prognosis [44].

### Analysis of independence and nomogram construction

Univariate and multivariate Cox regression analysis were used to assess the independence of prognostic signatures. Nomogram models were then constructed to predict CM patient survival [45].

### Immunogenomic landscape evaluation

The R “ESTIMATE” package was used to calculate the stromal and immune cell infiltration levels of each CM patient, and to compare the differences in immunophenoscores and immune cell infiltration levels between the high- and low-risk groups of CM patients [46]. Additionally, the Spearman correlation between DNA methylation sites and Immune Checkpoint-associated genes (ICGs) was analyzed, and Mann–Whitney U-test was used to compare the difference in ICGs expression values between CM patients in the high- and low-risk groups.

### Functional enrichment analysis

Differentially expressed genes (DEGs) between high- and low-risk group patients were determined using the R “limma” package, and adjusted *p* < 0.05, |logFC| ≥ 1 were considered statistically significant differences. Gene Ontology (GO) functional enrichment and Kyoto Encyclopedia of Genes and Genomes (KEGG) pathway analysis was conducted for the identified DEGs to validate their biological functions and pathways.

### Statistical analysis

Statistical analyses in this study were conducted using R.4.2.2. The Kaplan–Meier analysis was conducted to demonstrate the difference in prognosis between the two groups, and log-rank test was employed to identify the significance of differences. The Mann–Whitney-U test was conducted to illustrate the variables between the two groups. Correlation coefficients were assessed using spearman analysis. *p* values < 0.05 indicated statistical significance.

## Limitation

There are several limitations on the study. The CRG DNA methylation prognosis signature was based on the information of CM patients in the TCGA dataset, and it was verified only in the GEO datasets. More experimental validation has to be gathered in the future to reinforce the applicability of the signature. Simultaneously, the effect of signature in improving the quality of life in patients was still needed to be verified in clinical practice. Given the intricate etiology and development mechanisms of CM, abnormalities in the cuproptosis pathway may directly impact disease advancement in only a subgroup of individuals. Future research ought to explore strategies to discover this patient subgroup through stratification studies, as CRG DNA methylation prognosis signature might be more sensitive and specific within this population.

### Supplementary Information

Below is the link to the electronic supplementary material.

Supplementary file 1 (DOCX 5642 KB)

## Data Availability

Data is provided within the manuscript or supplementary information files.

## Abbreviations

CM: Cutaneous melanoma
TCGA: The Cancer Genome Atlas
GEO: Gene Expression Omnibus
CRG-located DNA-methylation prognostic signature: DNA-methylation located in cuproptosis death-related gene prognostic signature
AUC: Area under the curve
ROC: Receiver operating characteristic
CRGs: Cuproptosis death-related genes
TME: Tumor microenvironment
AOC1: Amine oxidase copper-containing 1
ACO1: Aconitase 1
DPYD: Dihydropyrimidine Dehydrogenas
ABCB8: ATP Binding Cassette Subfamily B Member 8
CIAO1: Cytosolic Iron-Sulfur Assembly Component 1
CI: Confidence interval
OS: Overall survival
ICGs: Immune checkpoint-associated genes
DEGs: Differentially expressed genes
GO: Gene Ontology
KEGG: Kyoto Encyclopedia of Genes and Genomes
EMT: Epithelial-to-mesenchymal transition
IREs: Iron-regulatory elements
