# Supplementary material for: Cuproptosis-related DNA methylation signature predict prognosis and immune microenvironment in cutaneous melanoma
Source: Discov Oncol. 2024 Jun 14;15:228. doi: 10.1007/s12672-024-01089-8 (PMC11178724; doi:10.1007/s12672-024-01089-8)
Supplement: Supplementary file 1 — (DOCX 5642 KB) [file 12672_2024_1089_MOESM1_ESM.docx]

**Supplementary Material**


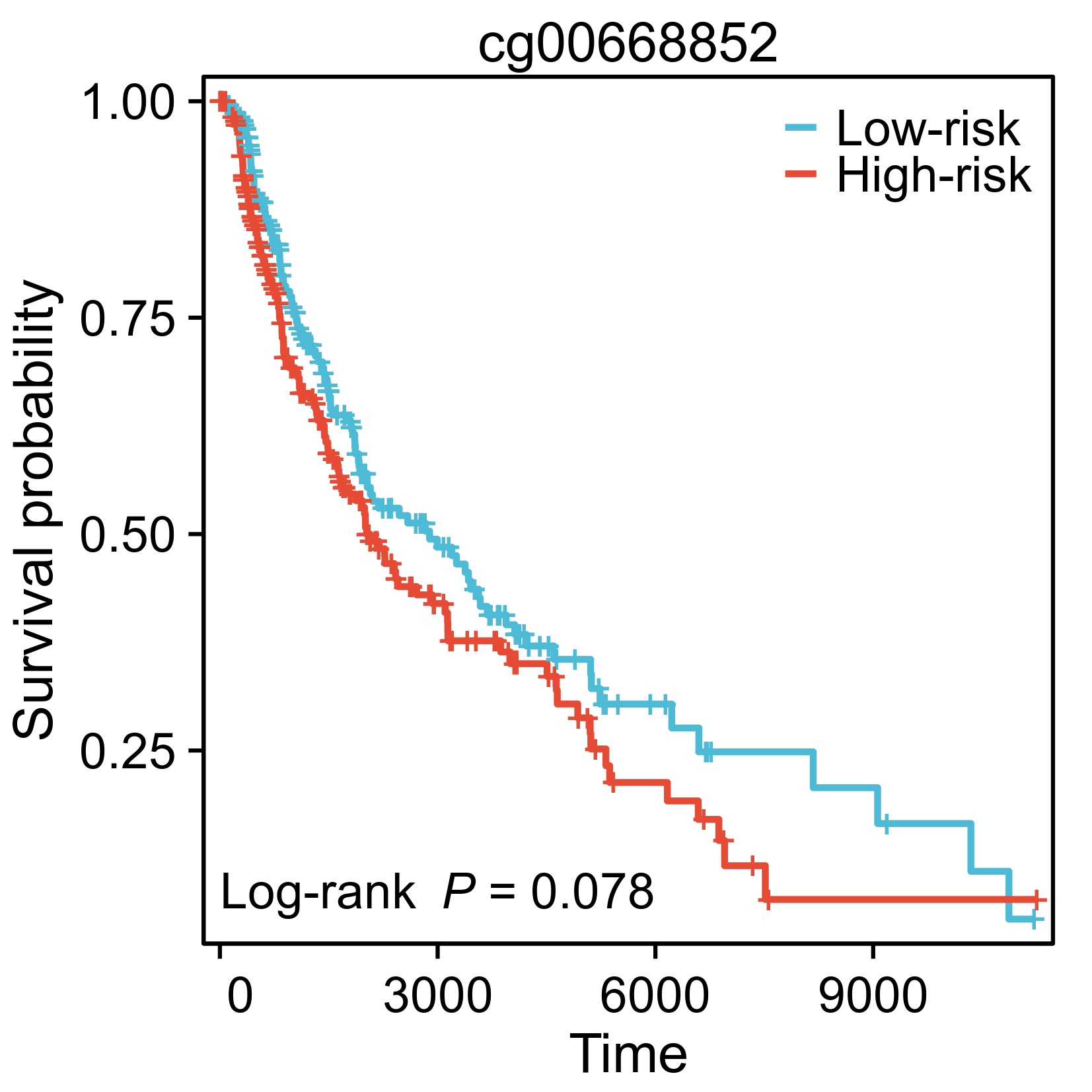

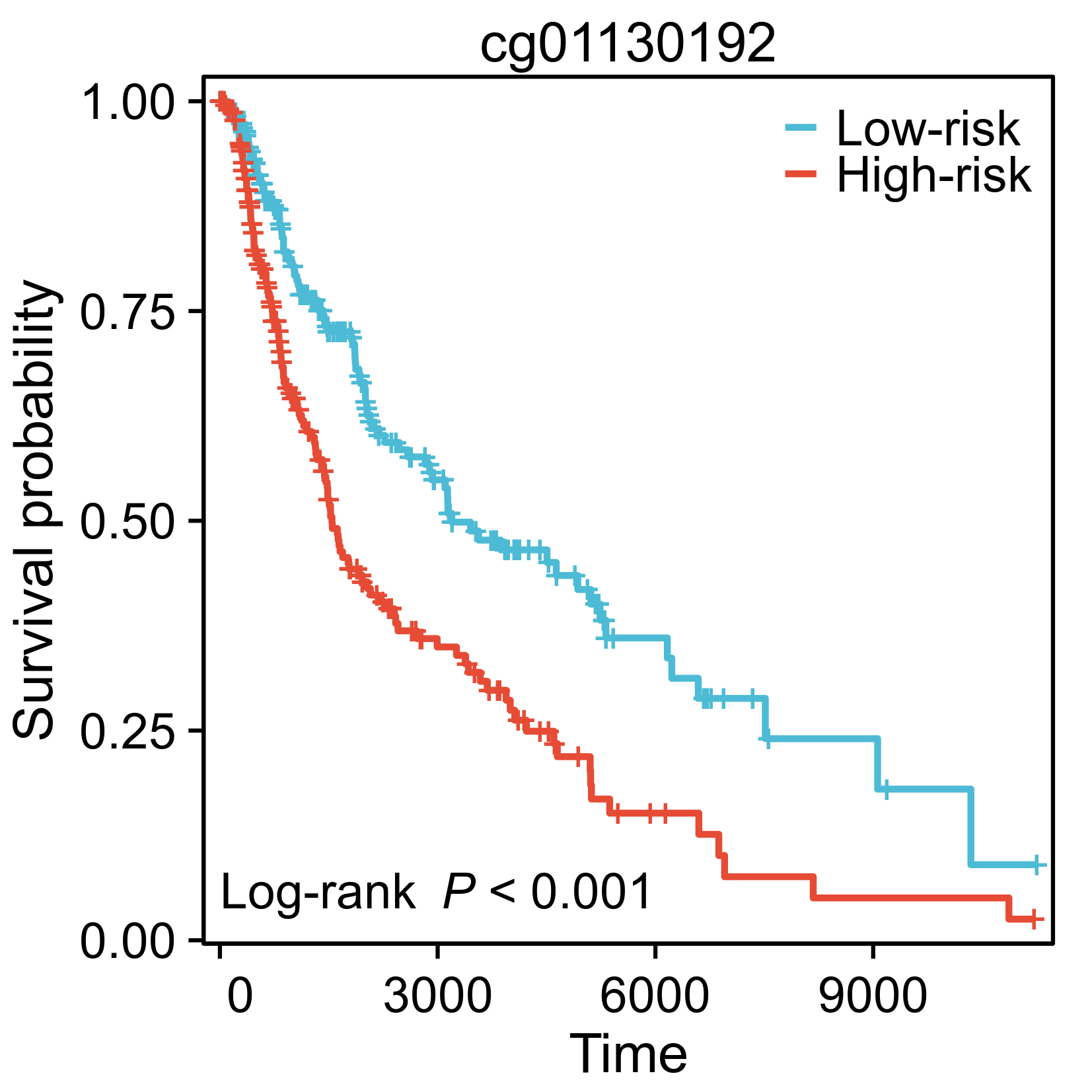


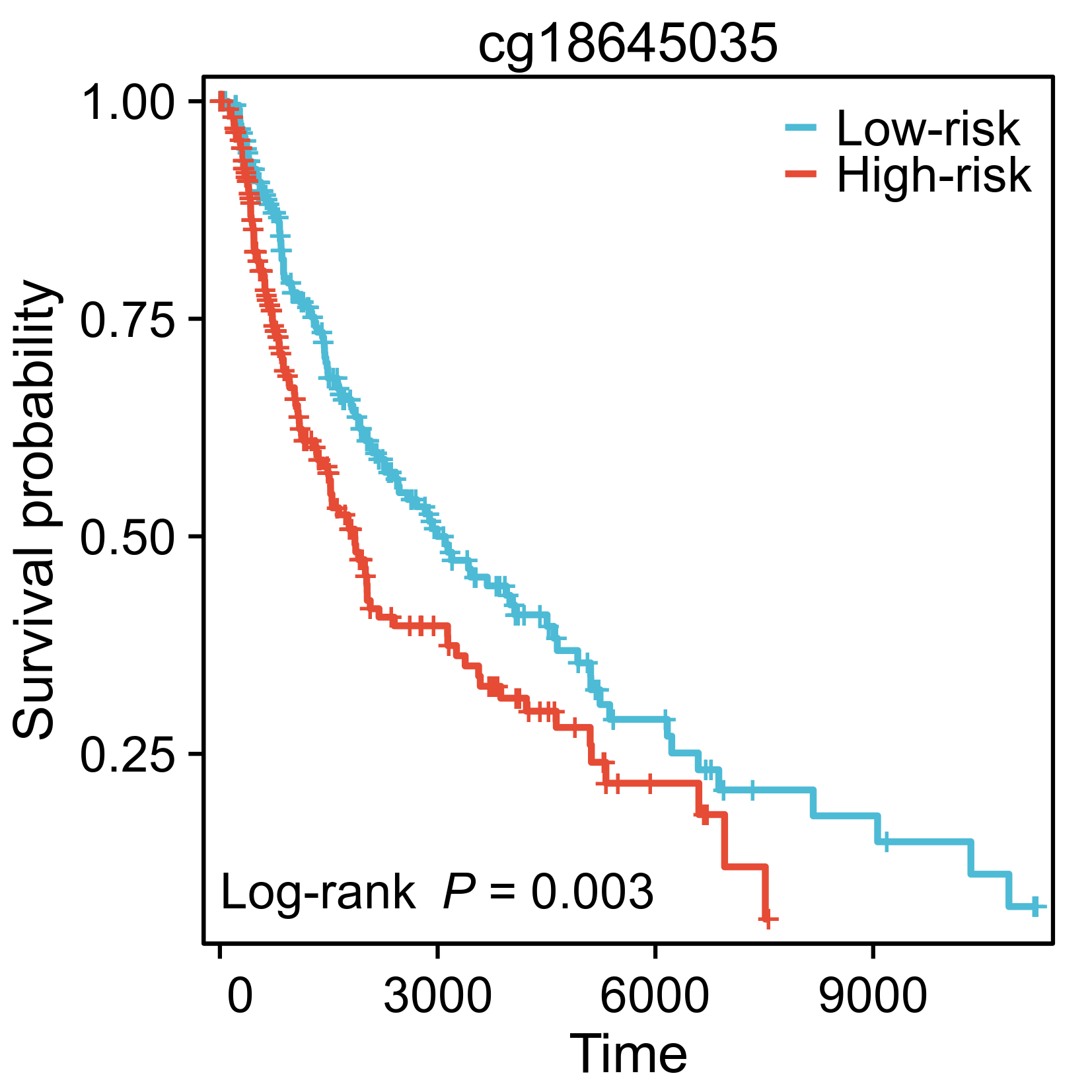

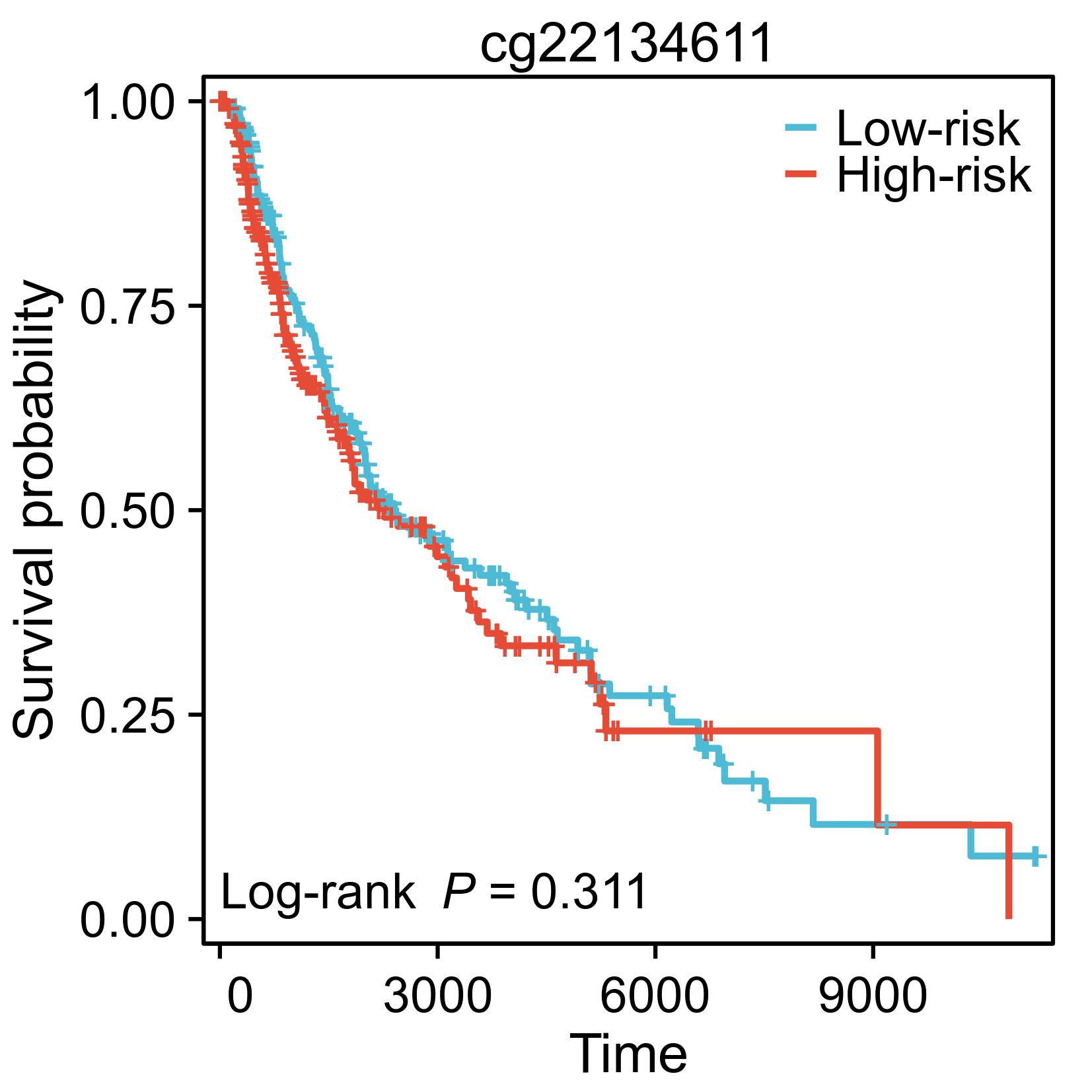

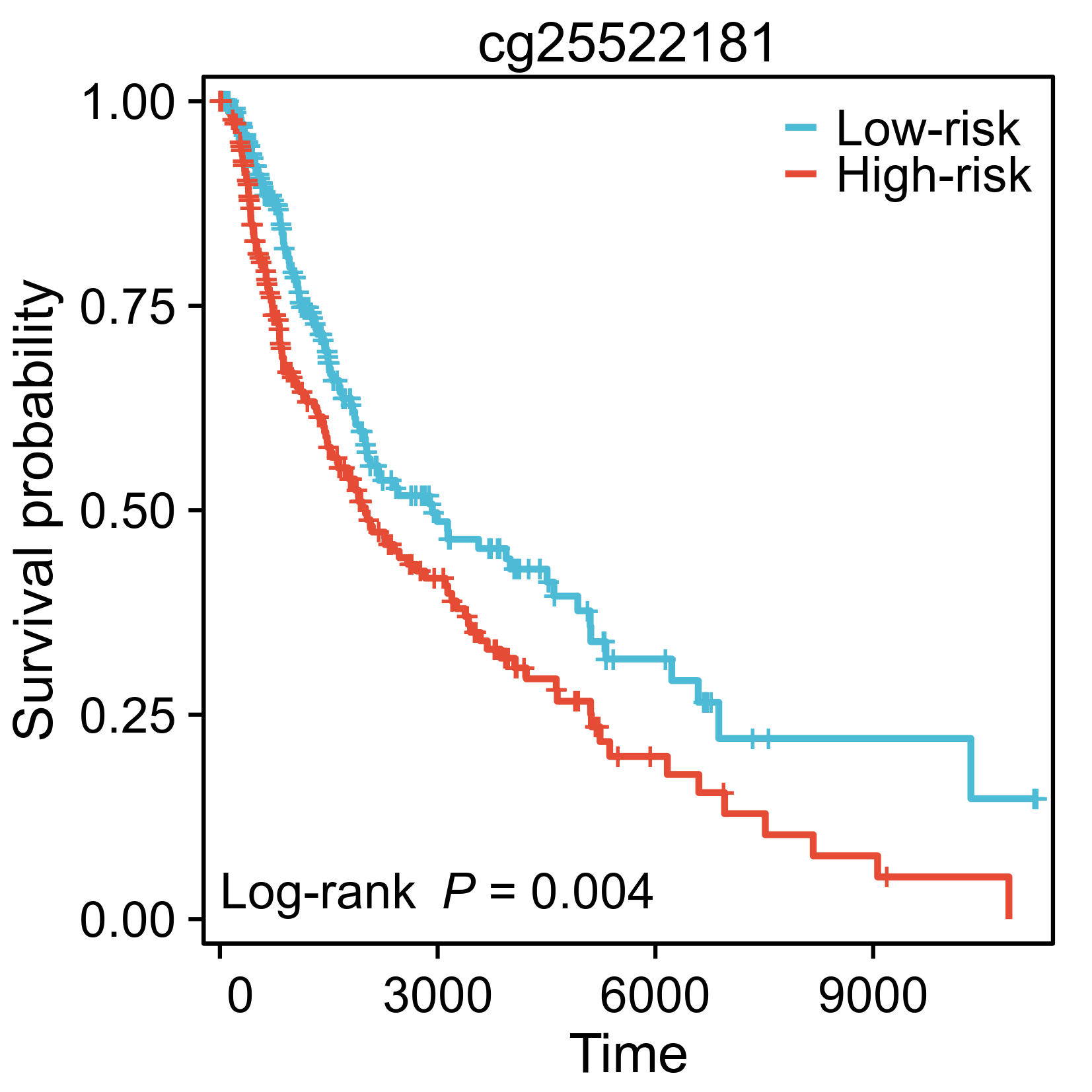

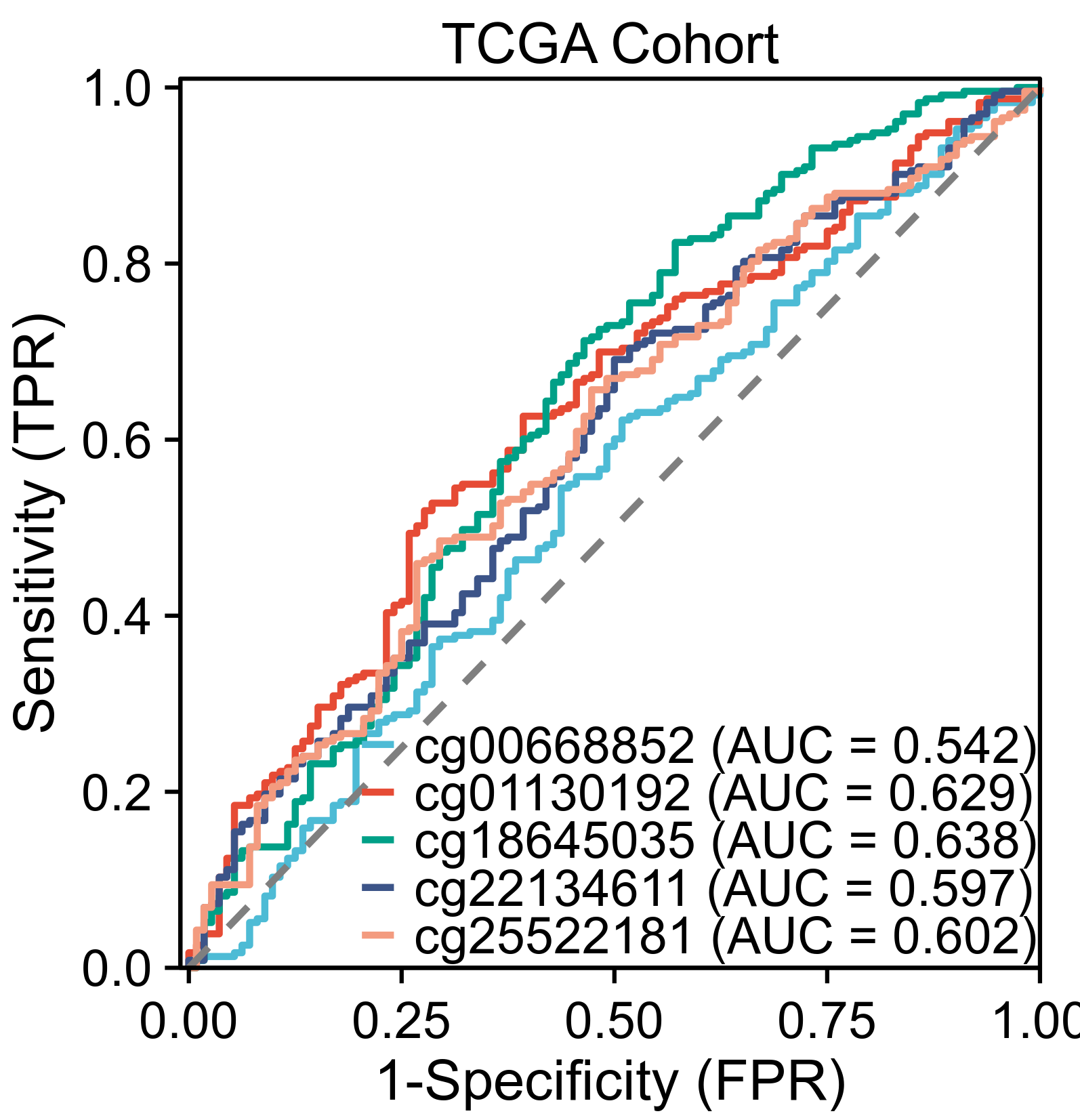


Figure S1. Prognostic value of five CRG-located DNA methylation sites in the TCGA cohort. The units of time are days.


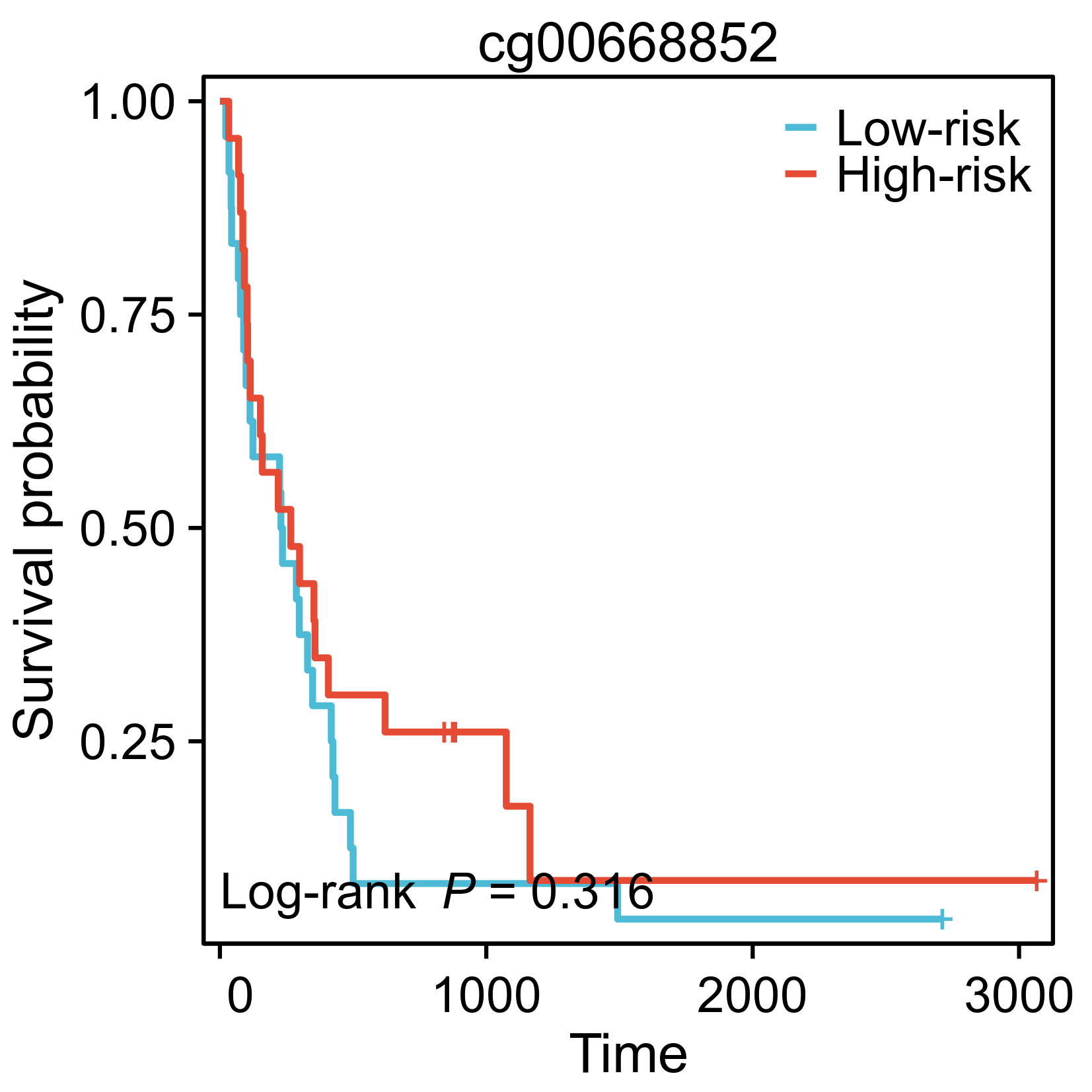

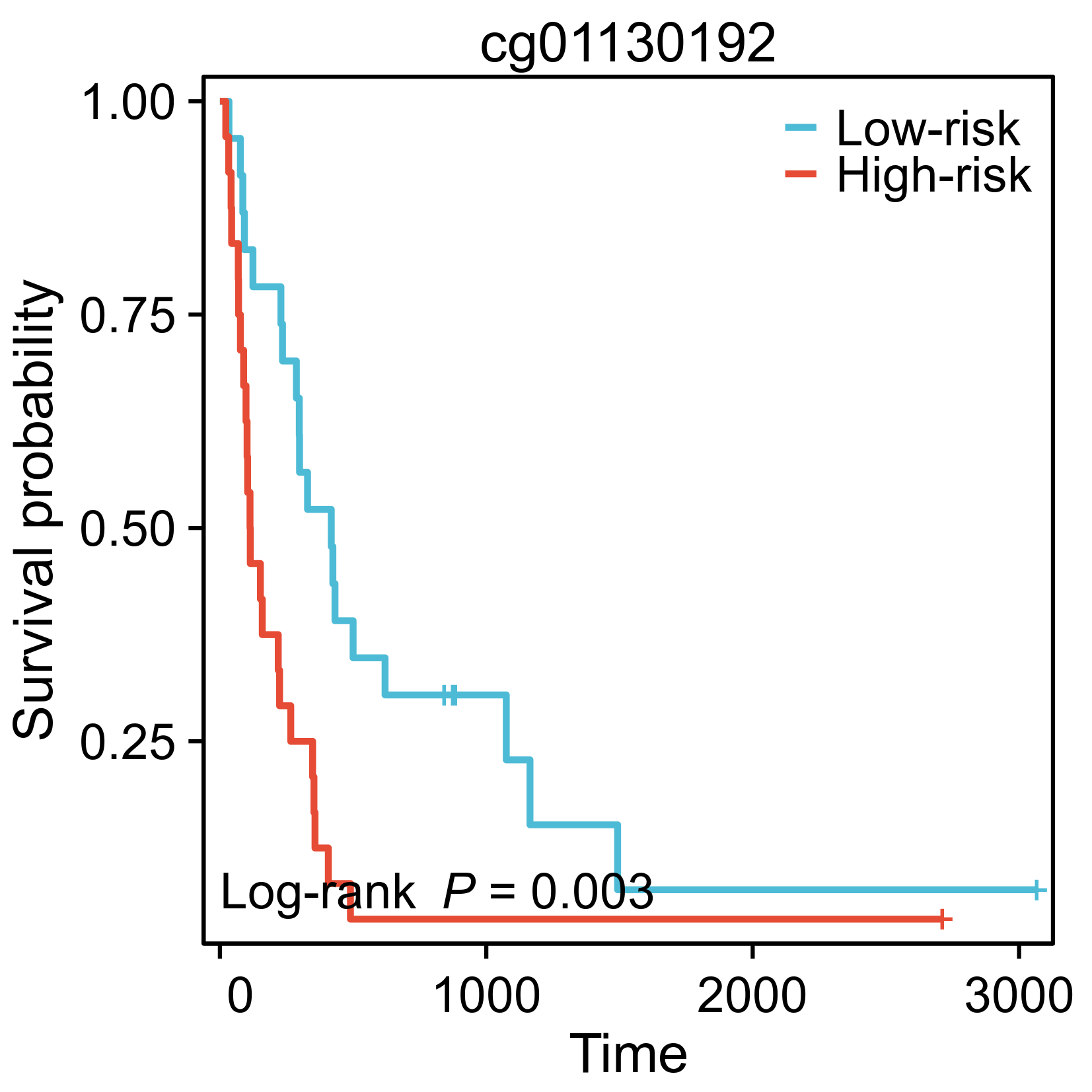

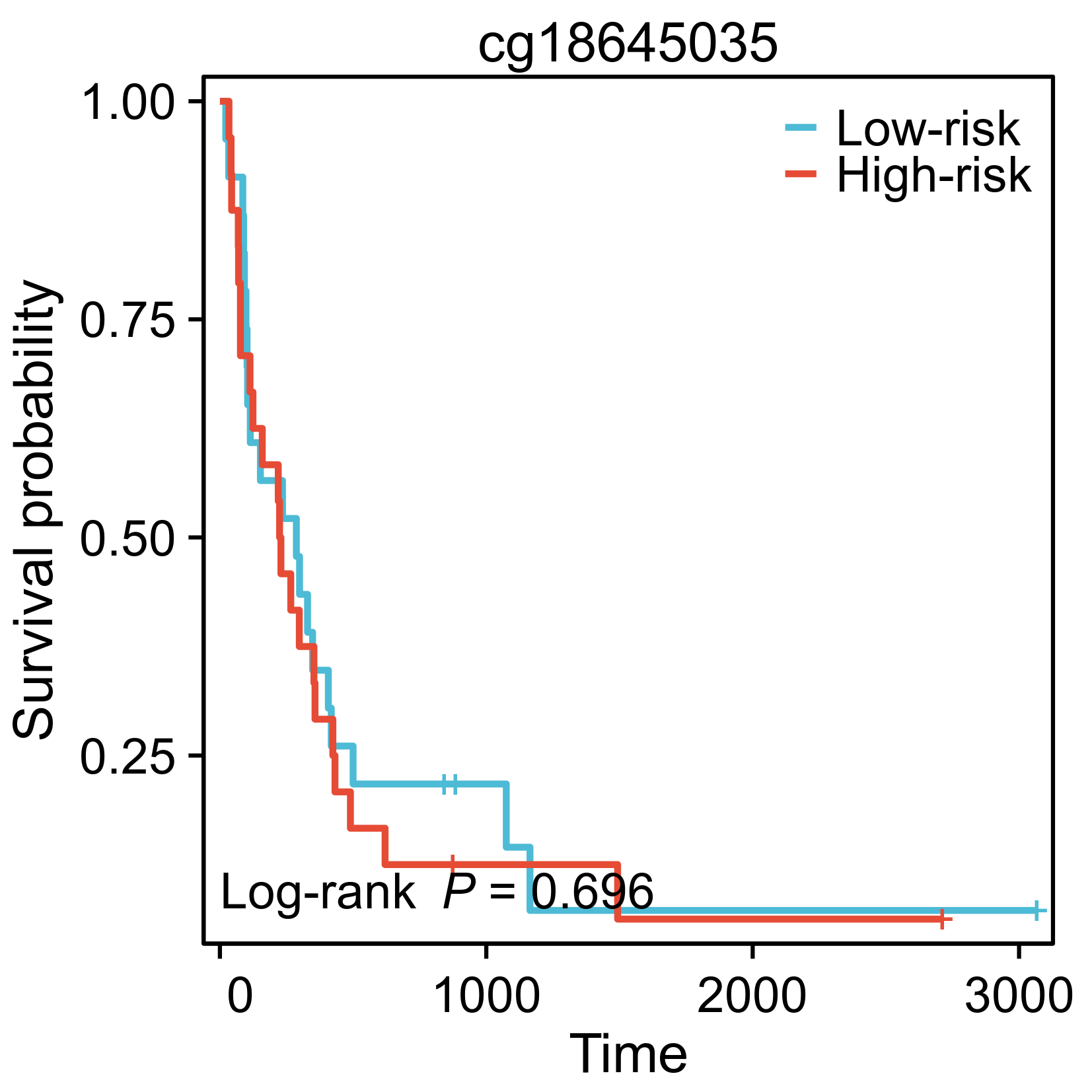

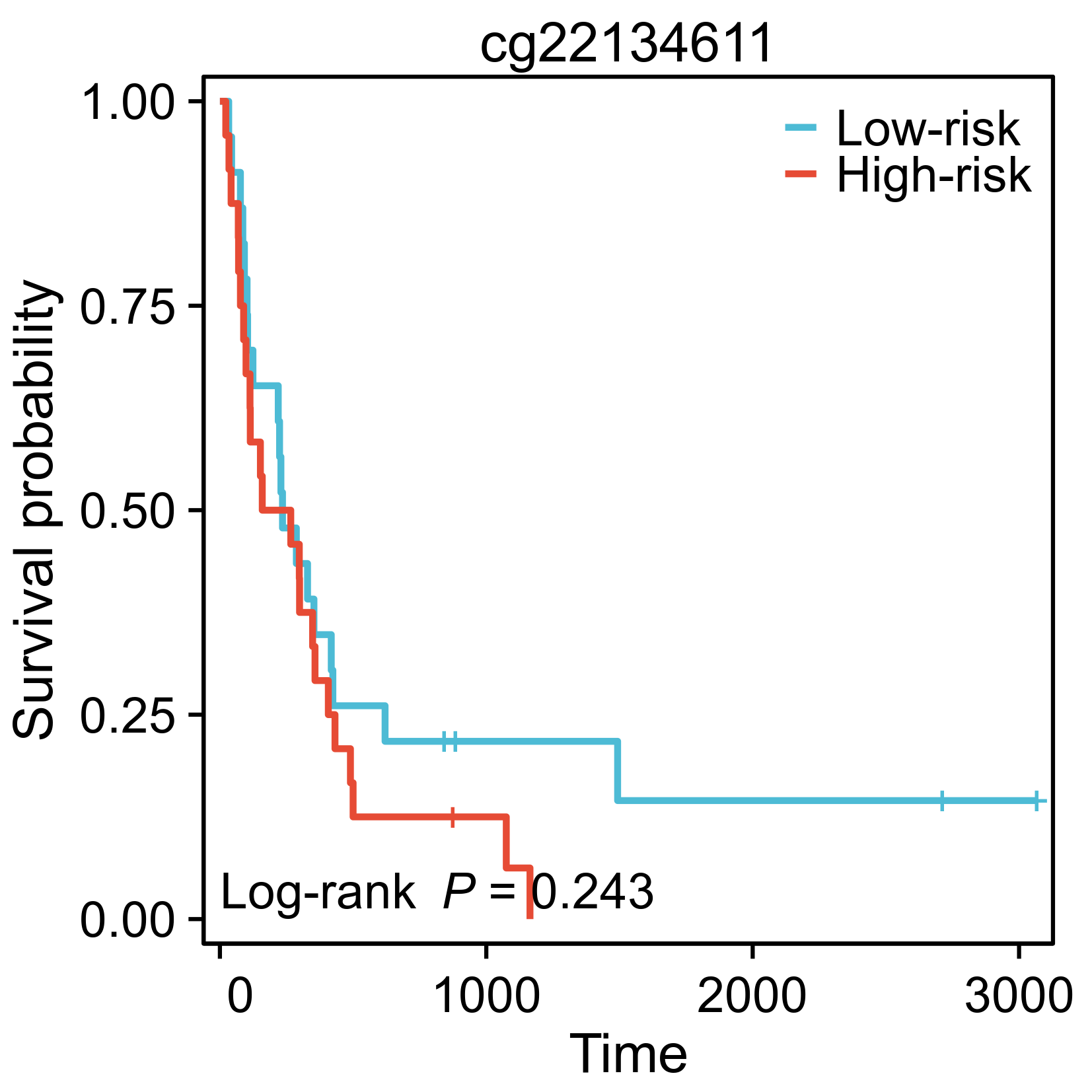

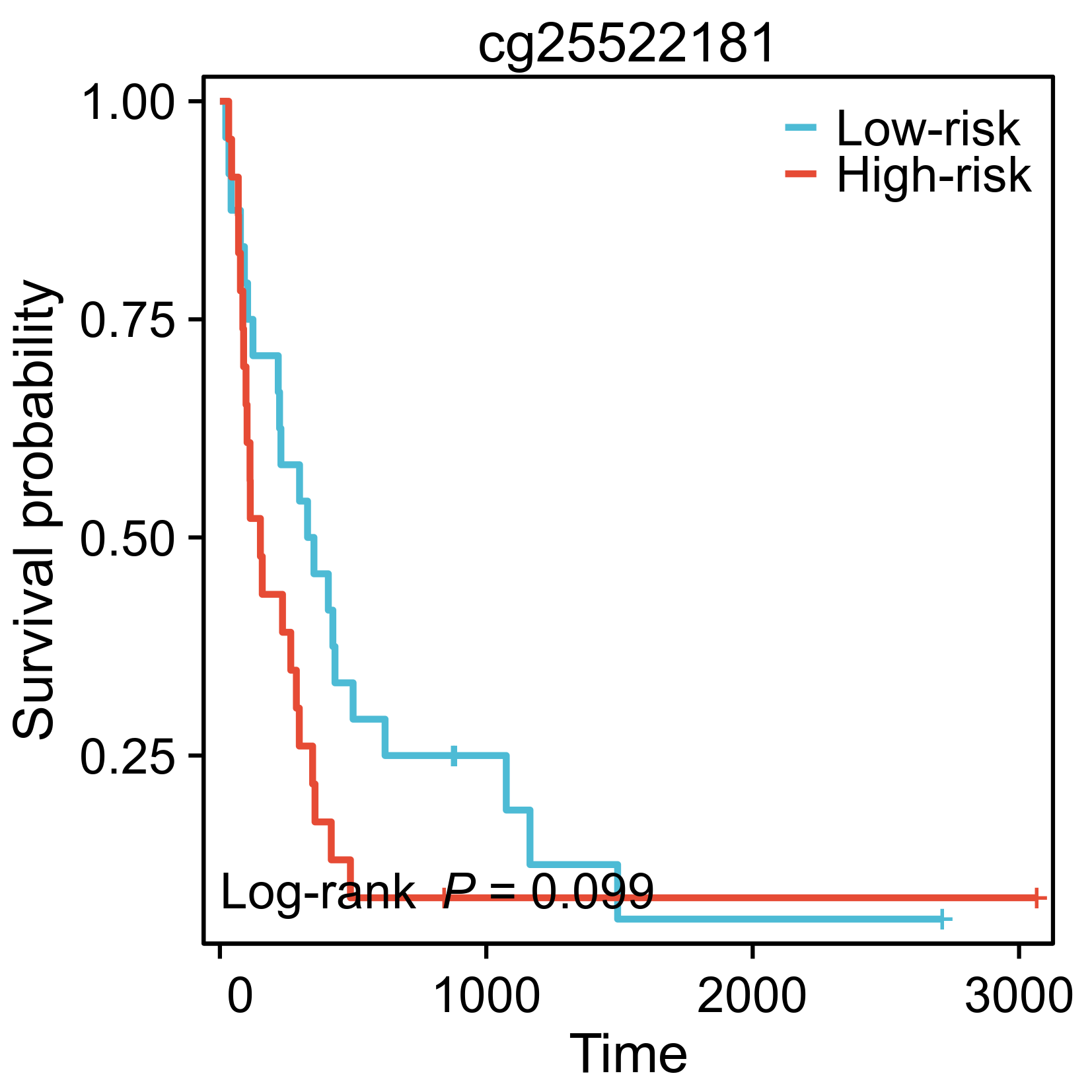

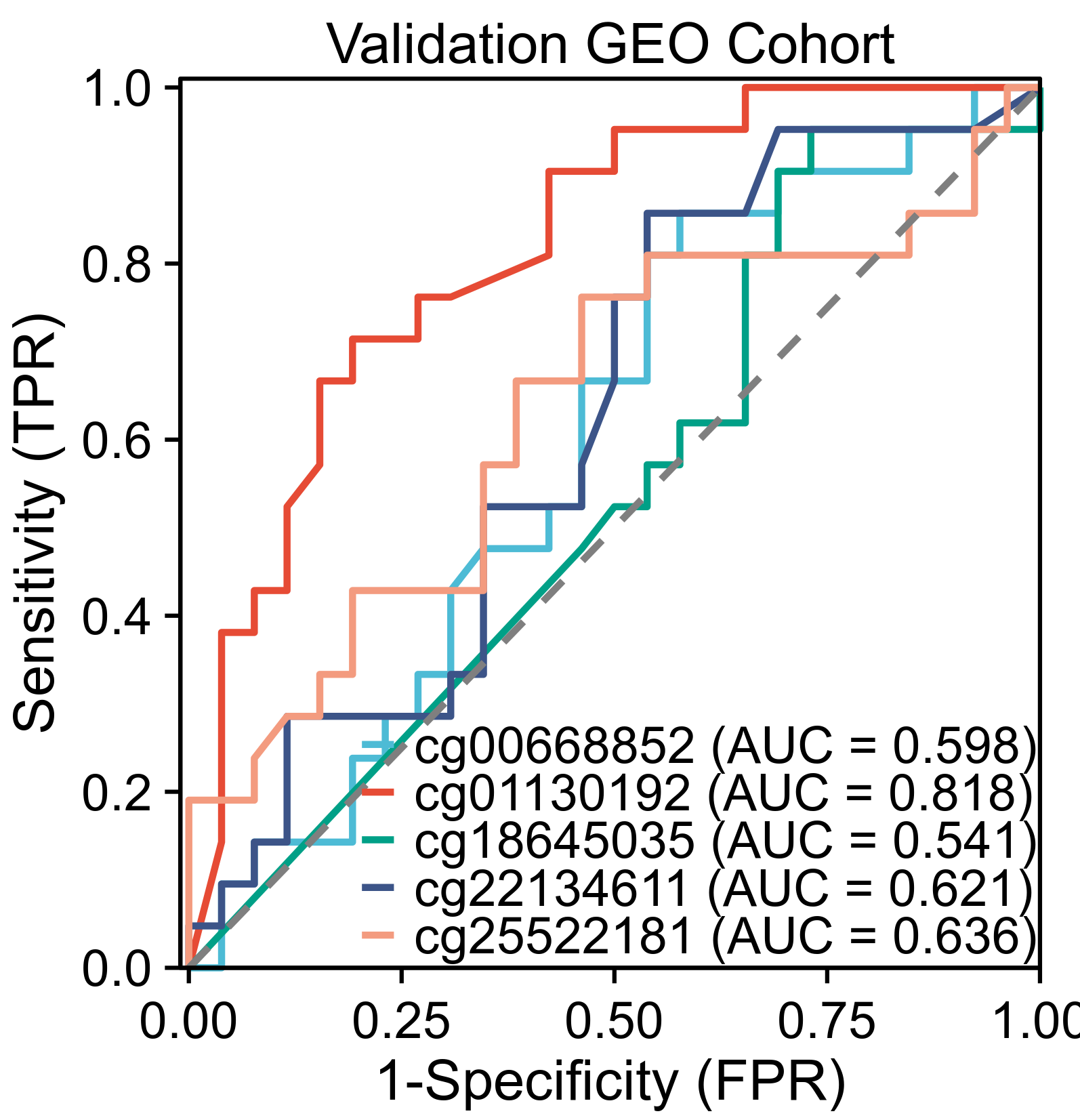


Figure S2. Prognostic value of five CRG-located DNA methylation sites in the GEO cohort. The units of time are days.


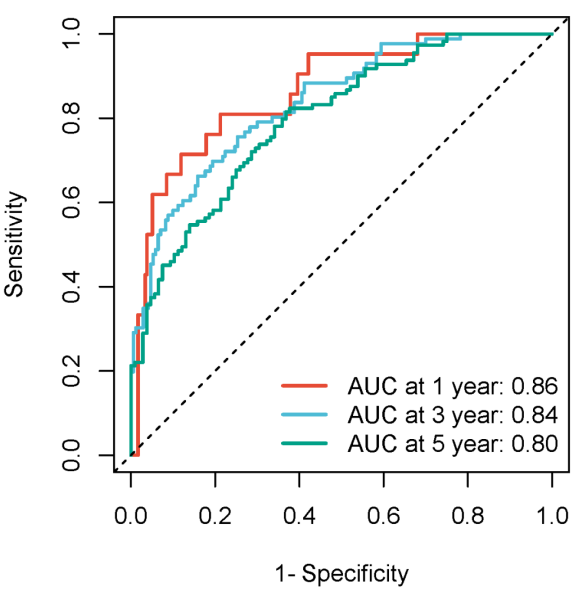


Figure S3. Performance evaluation of the nomogram in the TCGA cohort.


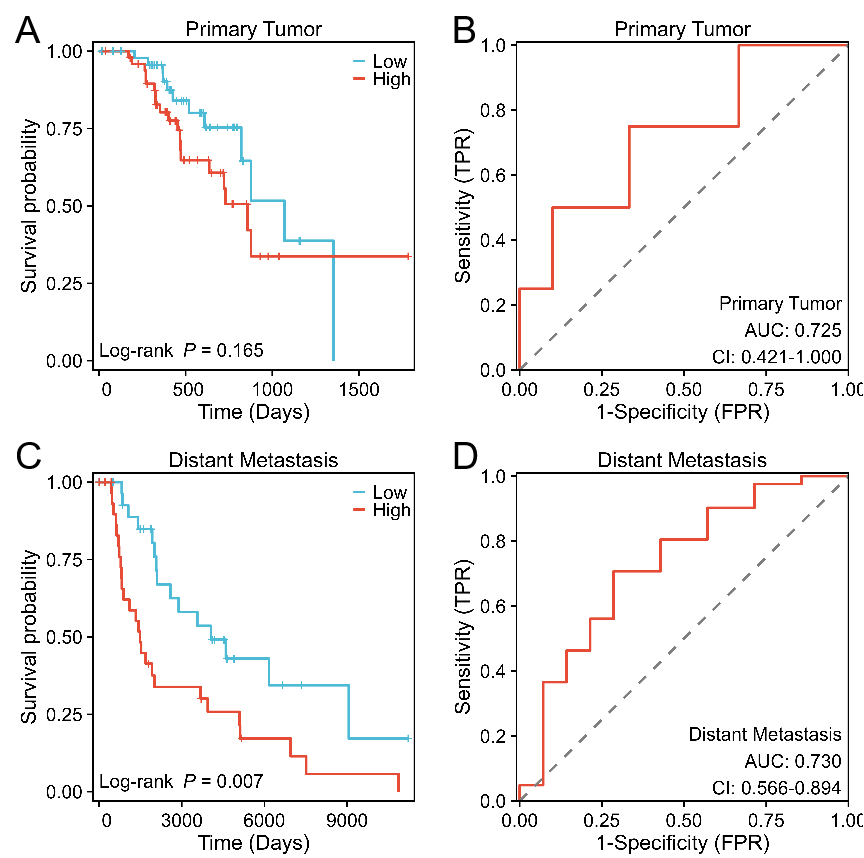

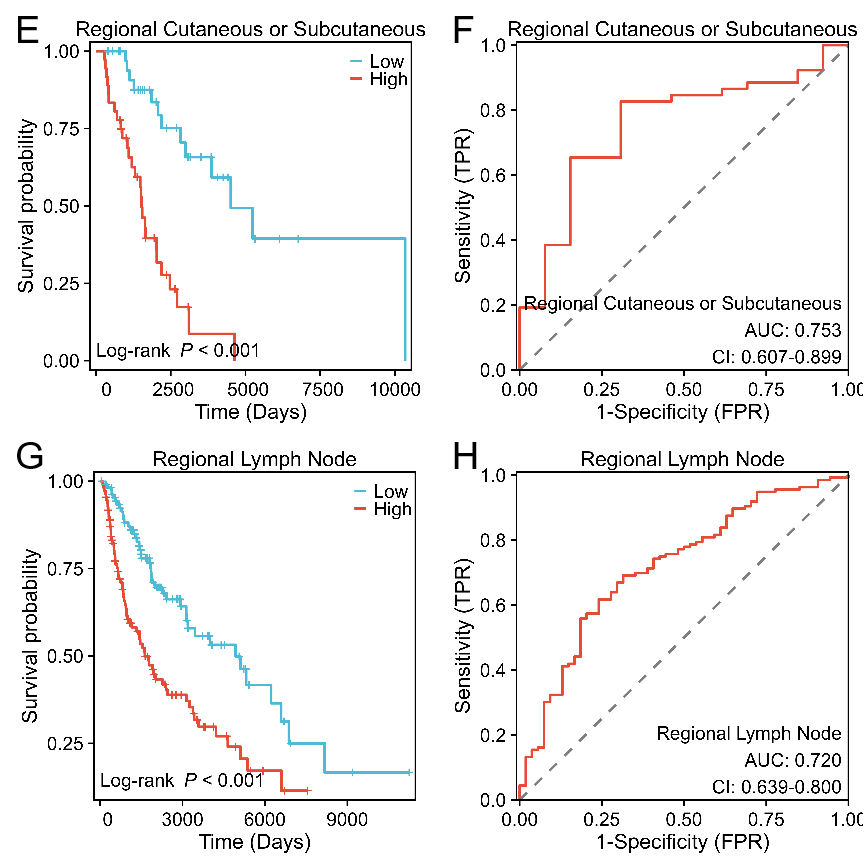


Figure S4. Kaplan-Meier and ROC analyses of DNA methylation prognostic signature of patients with different Tumor locations in the TCGA cohort.


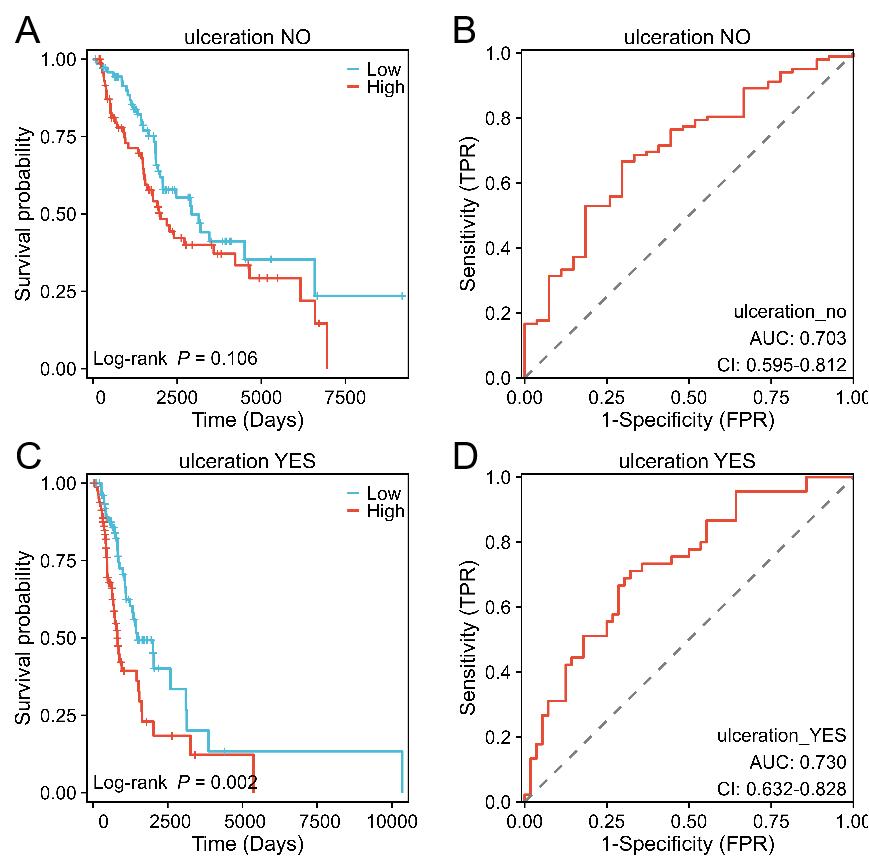


**Figure S5**. Kaplan-Meier and ROC analyses of DNA methylation prognostic signature of patients with different ulceration indicators in the TCGA cohort.


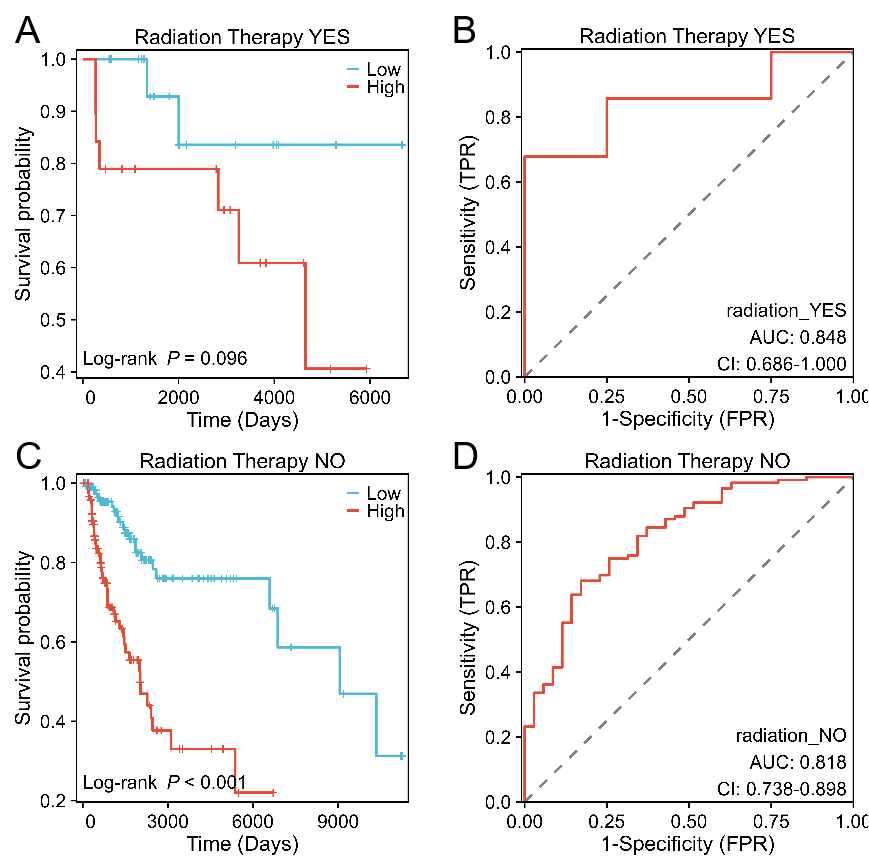


**Figure S6**. Kaplan-Meier and ROC analyses of DNA methylation prognostic signature of patients with different radiation therapy in the TCGA cohort.


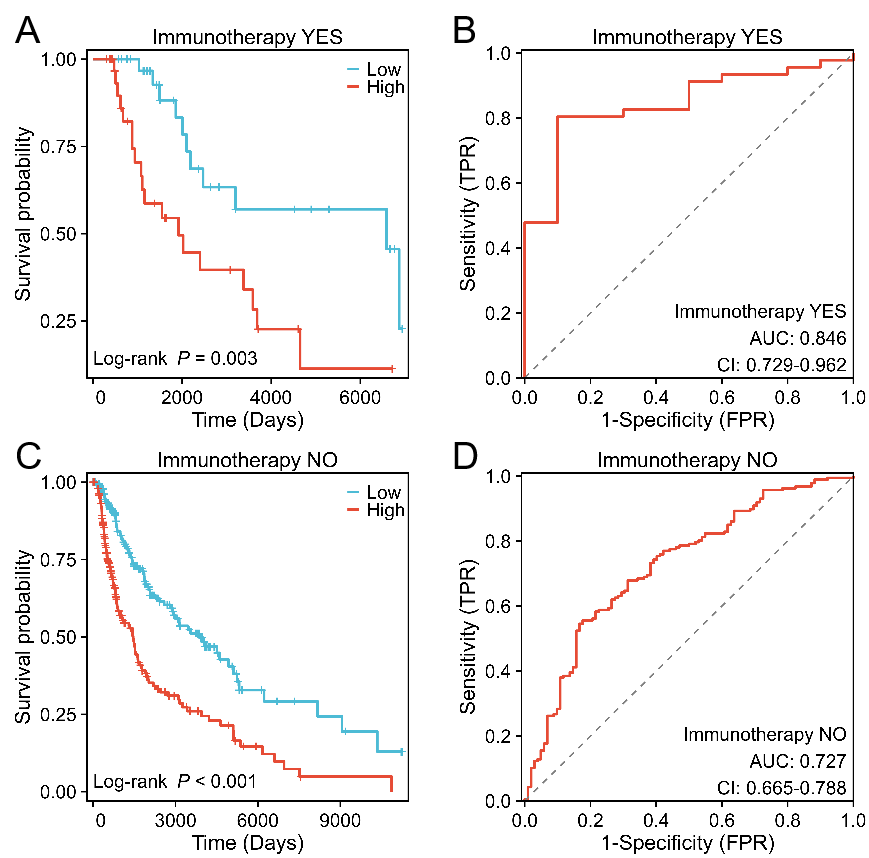


**Figure S7**. Kaplan-Meier and ROC analyses of DNA methylation prognostic signature of patients with different immunotherapy in the TCGA cohort.


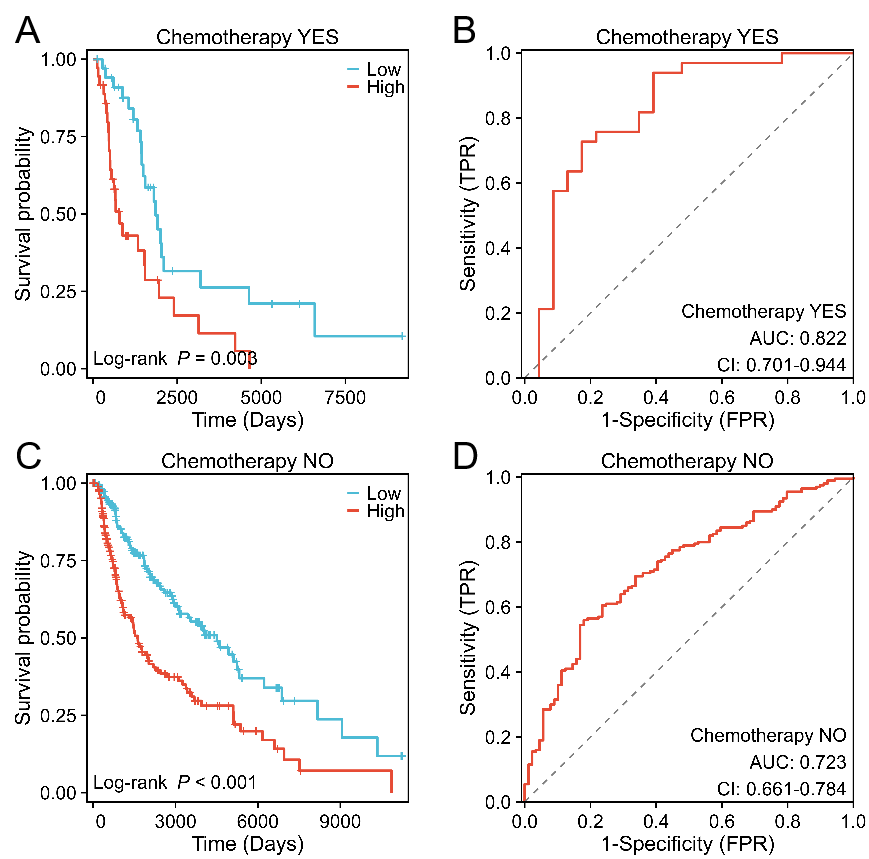


**Figure S8**. Kaplan-Meier and ROC analyses of DNA methylation prognostic signature of patients with different chemotherapy in the TCGA cohort.


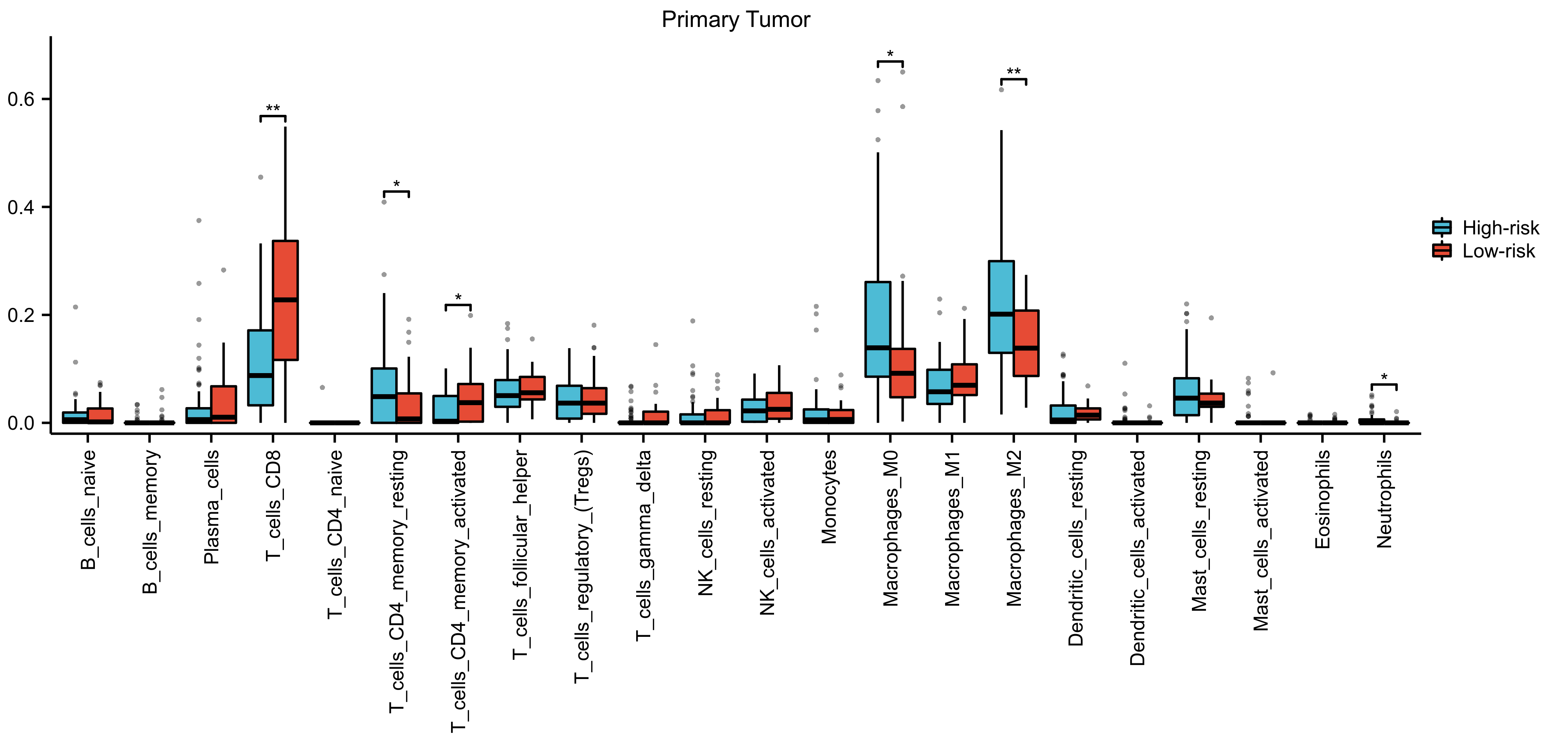

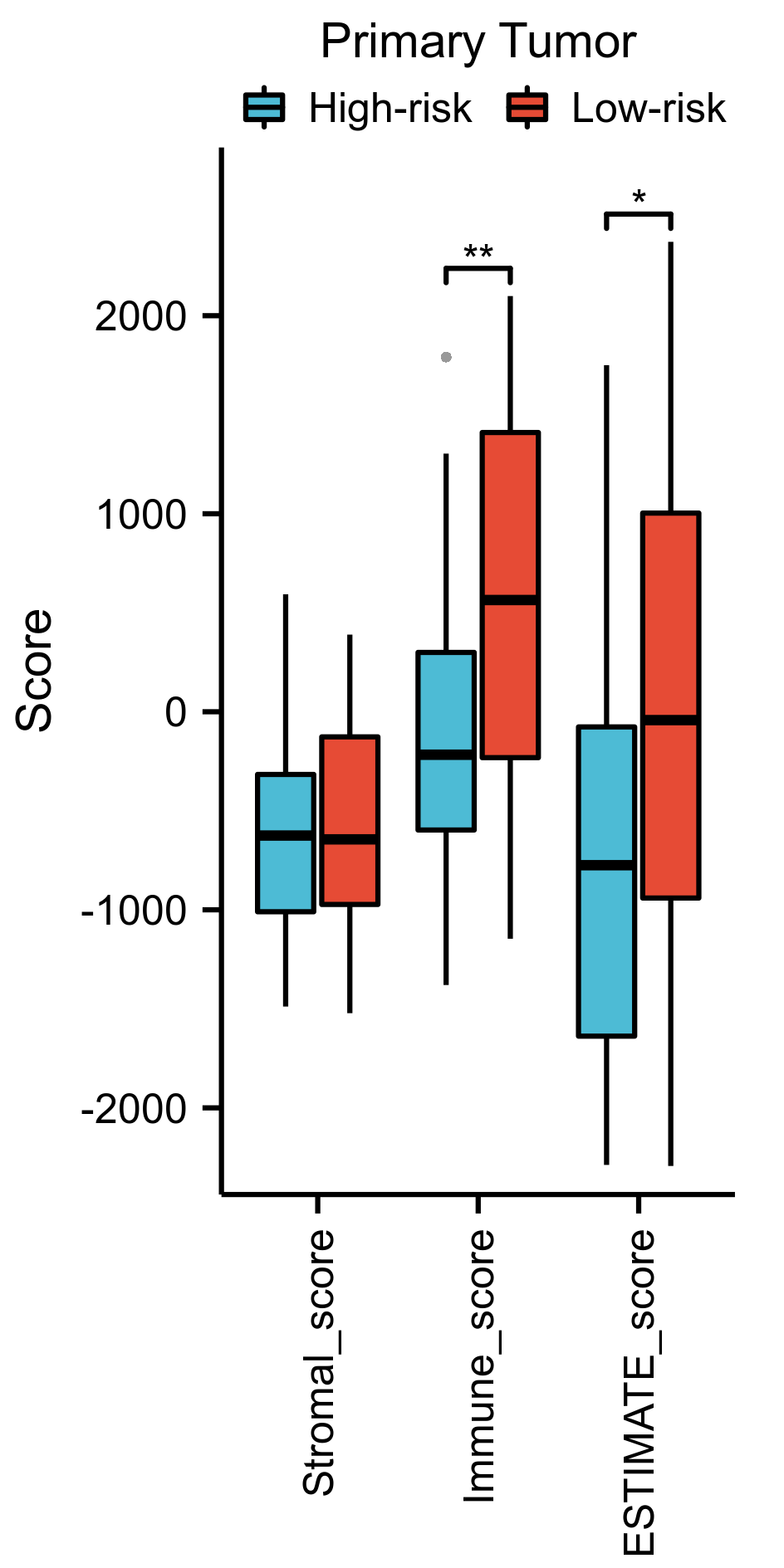


**Figure S9.** The differences in 22 types of immune infiltration, stromal scores (the level of stromal cells present in tumor tissue), immune scores (the infiltration level of immune cells in tumor tissues), ESTIMATE scores (tumor purity) between high- and low-risk in the primary tumor patients of TCGA cohort.


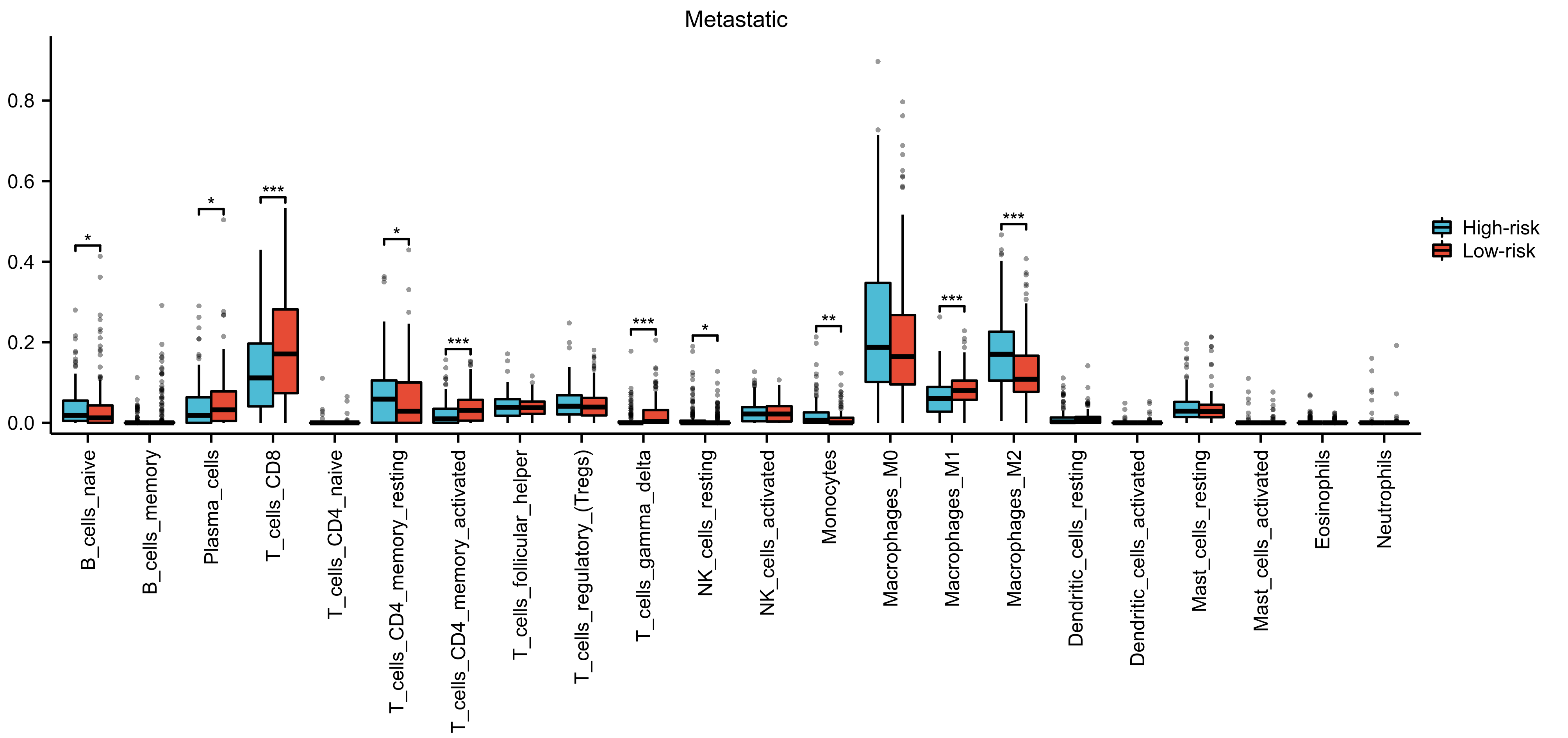

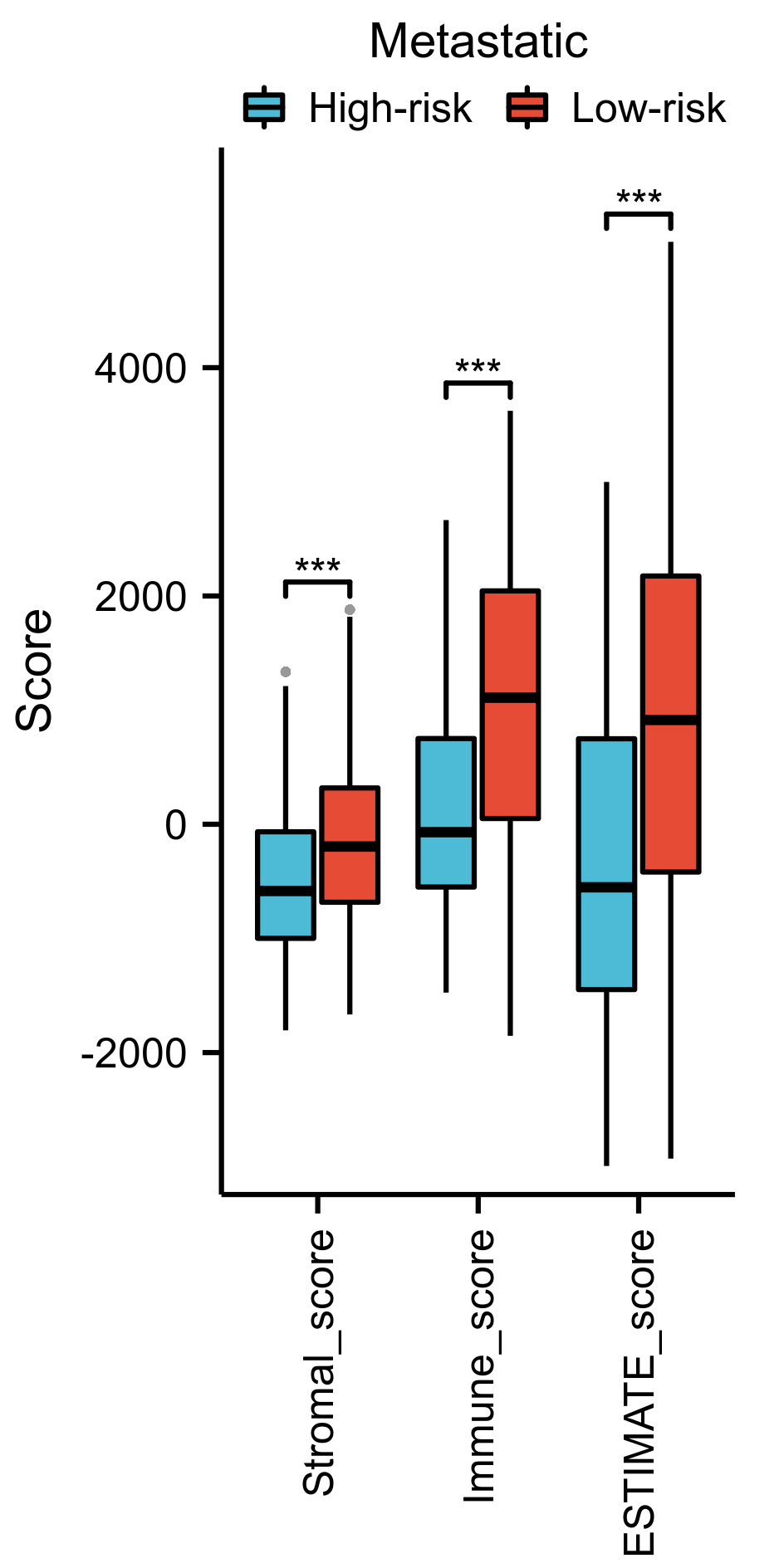


**Figure S10.** The differences in 22 types of immune infiltration, stromal scores (the level of stromal cells present in tumor tissue), immune scores (the infiltration level of immune cells in tumor tissues), ESTIMATE scores (tumor purity) between high- and low-risk in the metastatic patients of TCGA cohort.


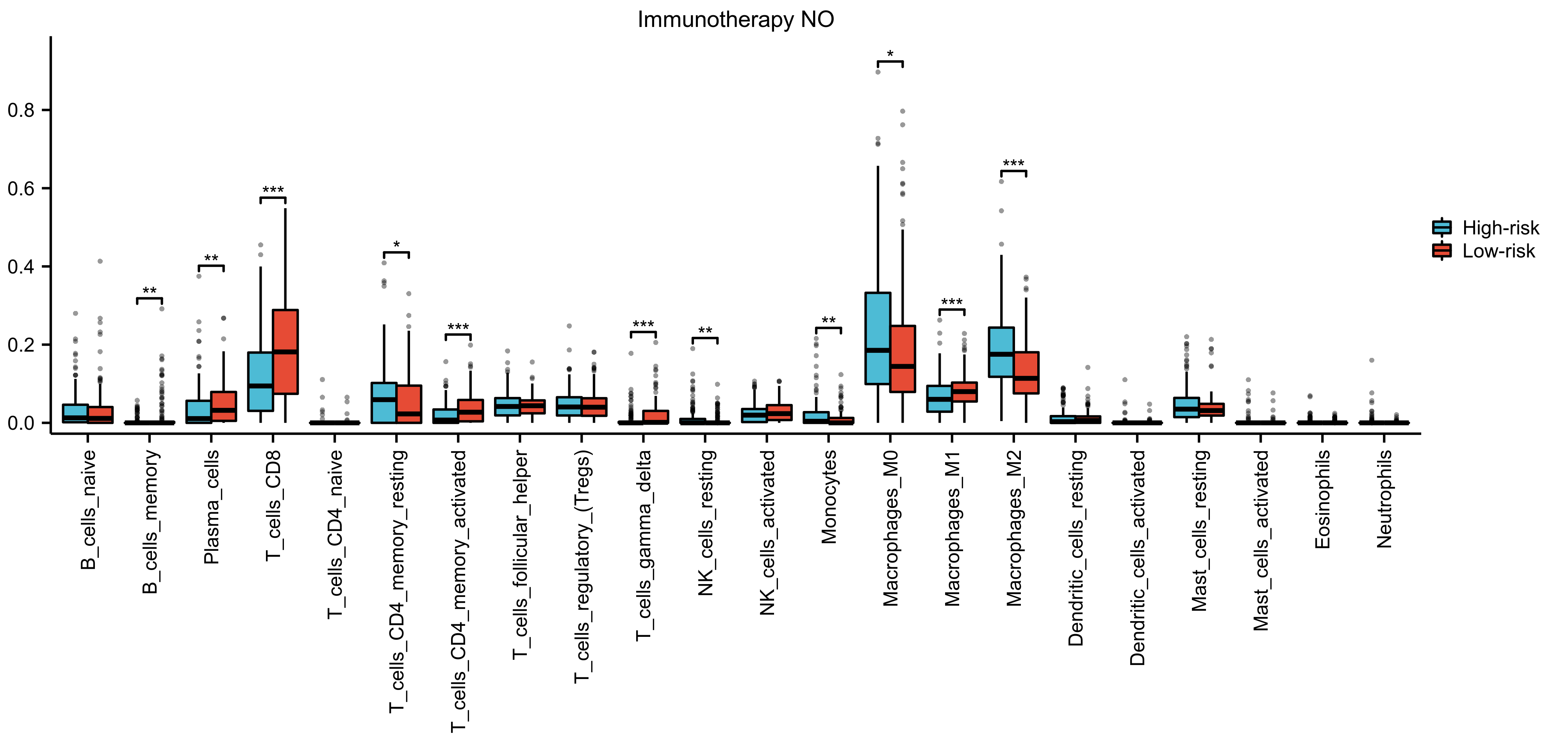

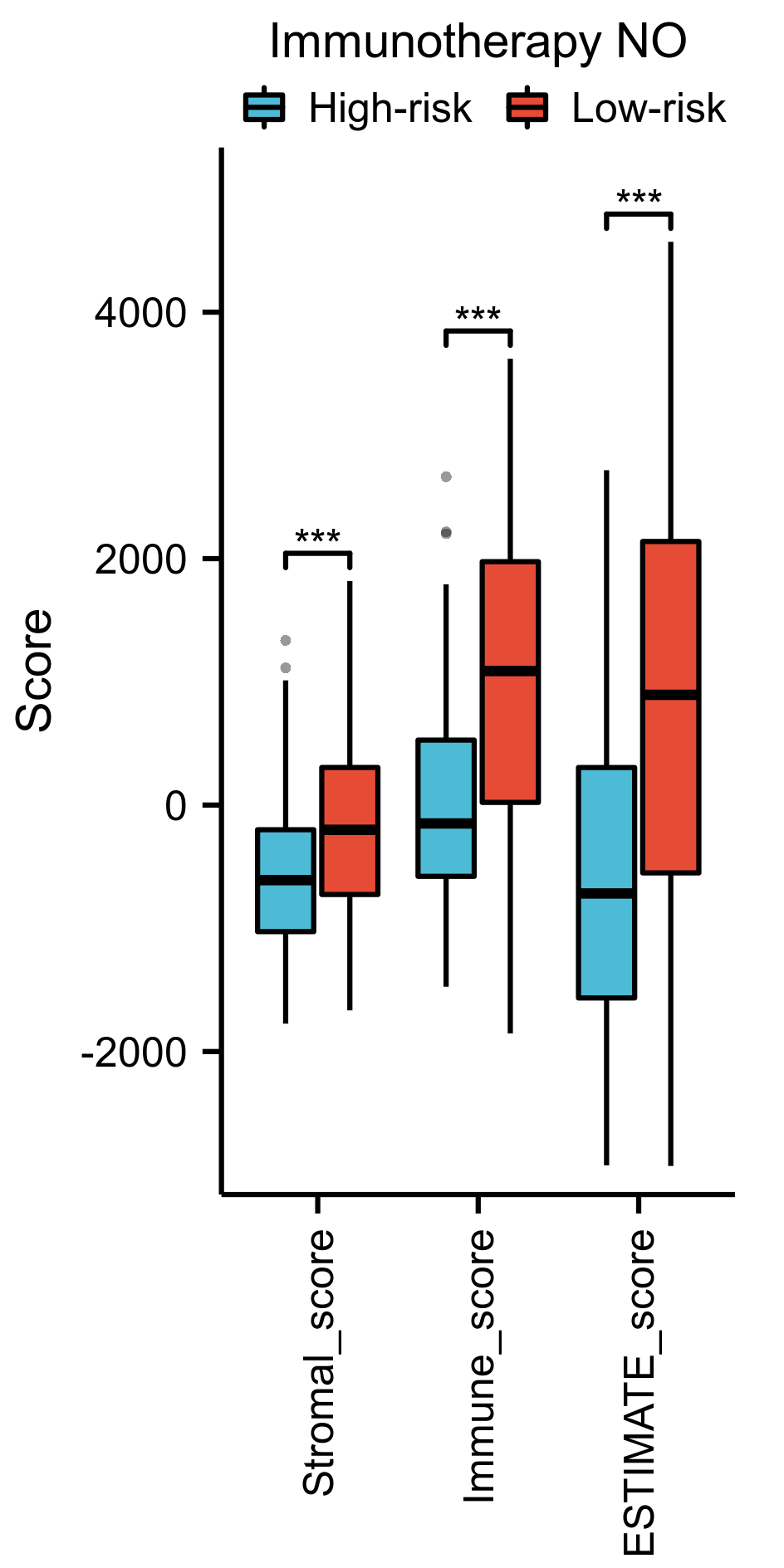


**Figure S11.** The differences in 22 types of immune infiltration, stromal scores (the level of stromal cells present in tumor tissue), immune scores (the infiltration level of immune cells in tumor tissues), ESTIMATE scores (tumor purity) between high- and low-risk in the non-immunotherapy patients of TCGA cohort.


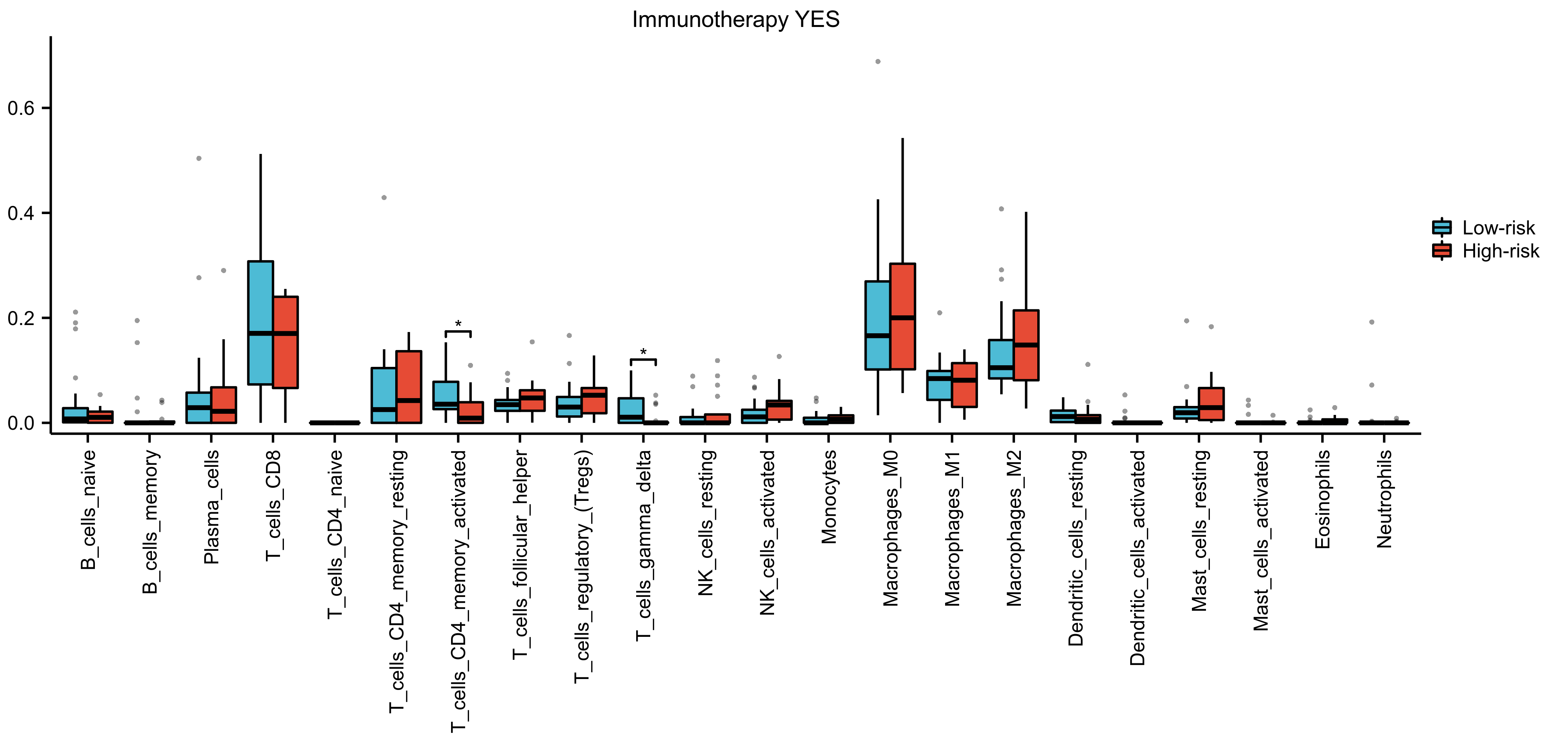

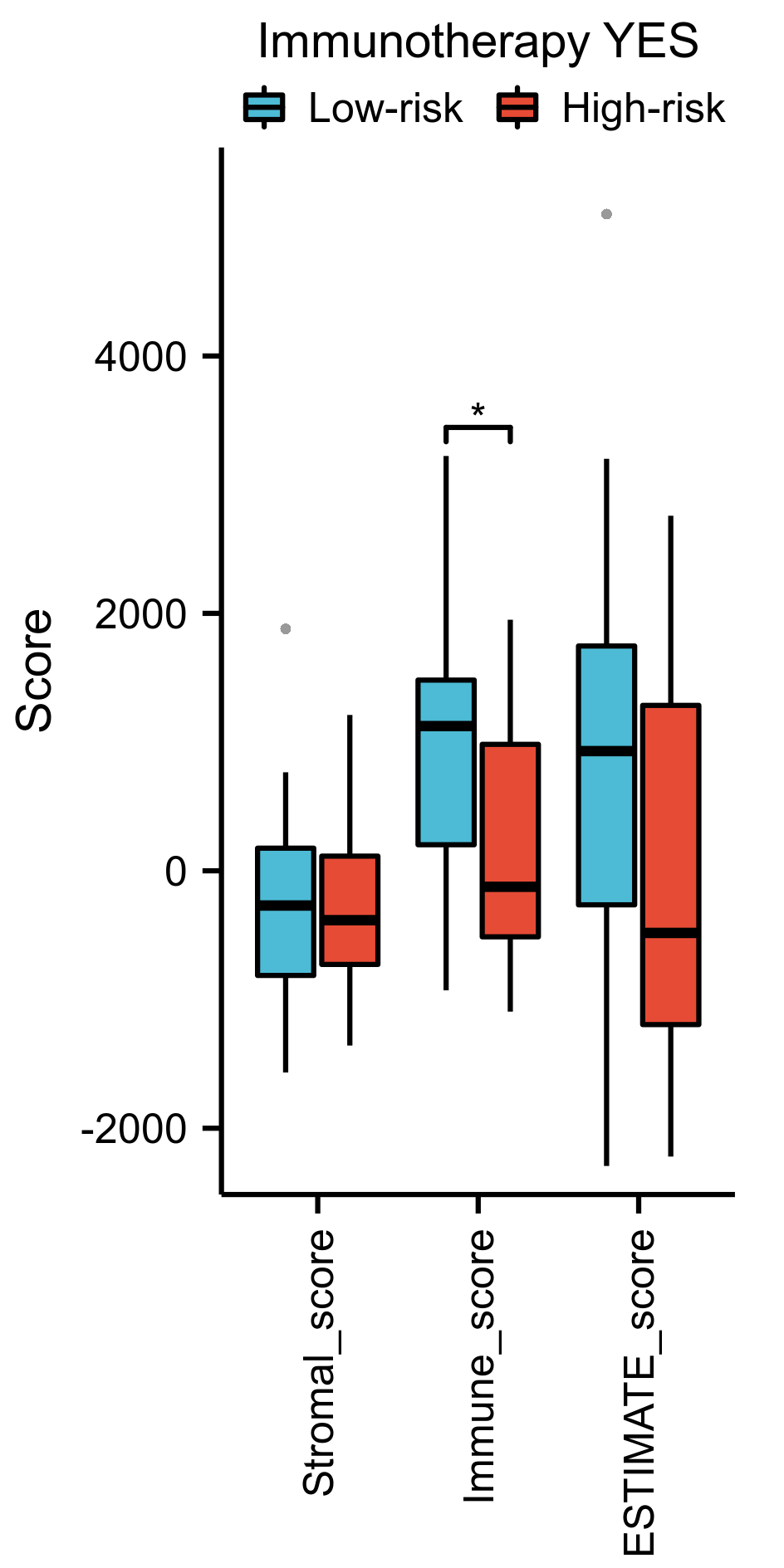


**Figure S12.** The differences in 22 types of immune infiltration, stromal scores (the level of stromal cells present in tumor tissue), immune scores (the infiltration level of immune cells in tumor tissues), ESTIMATE scores (tumor purity) between high- and low-risk in patients with immunotherapy of TCGA cohort.


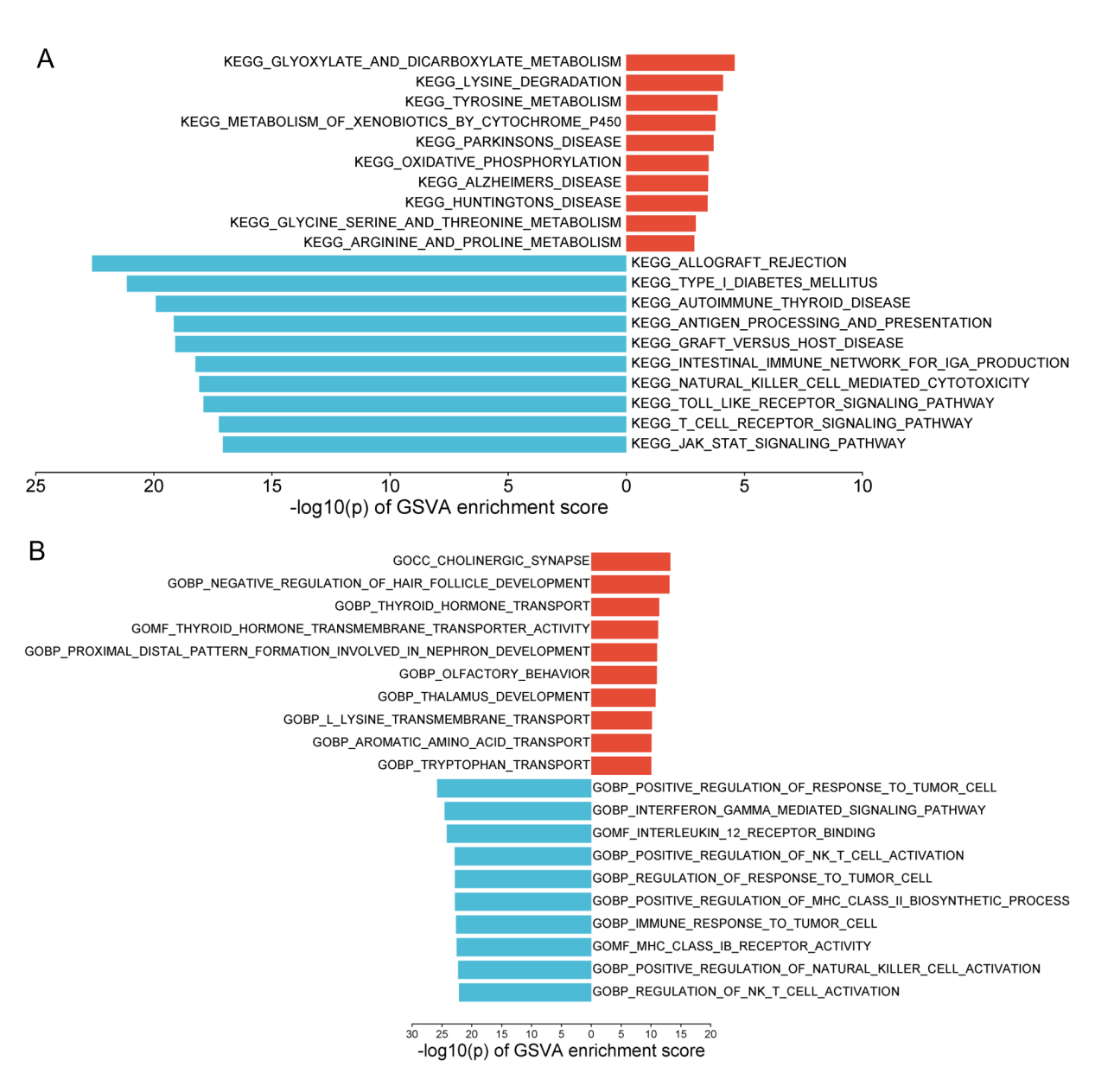


**Figure S13.** GSVA analysis of the CRG DNA methylation prognostic signature between low- and high-risk group based on KEGG pathway (A) and GO term (B).


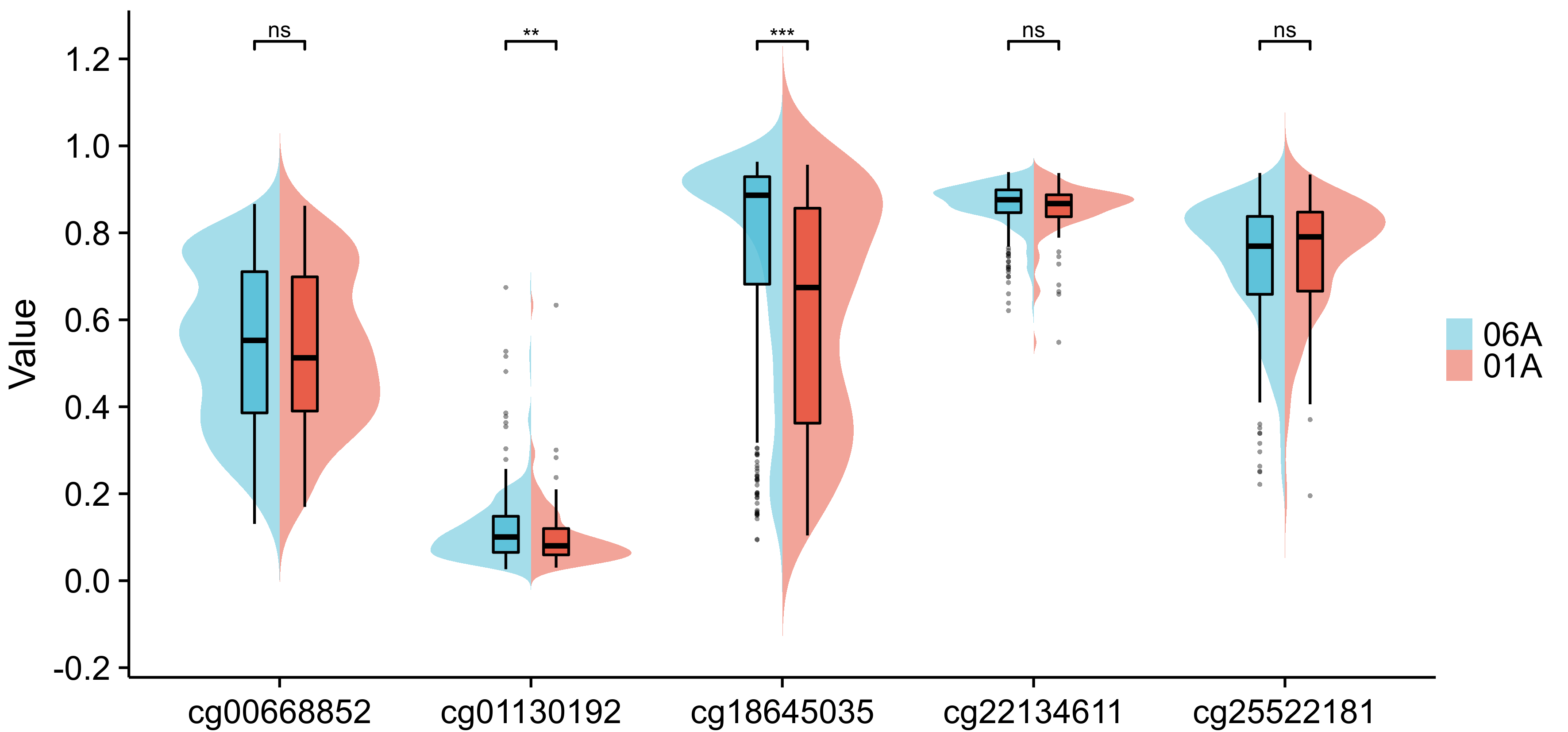


**Figure S14.** Differences for methylation level of sites between primary tissue (01A) and metastatic tissue (06A).


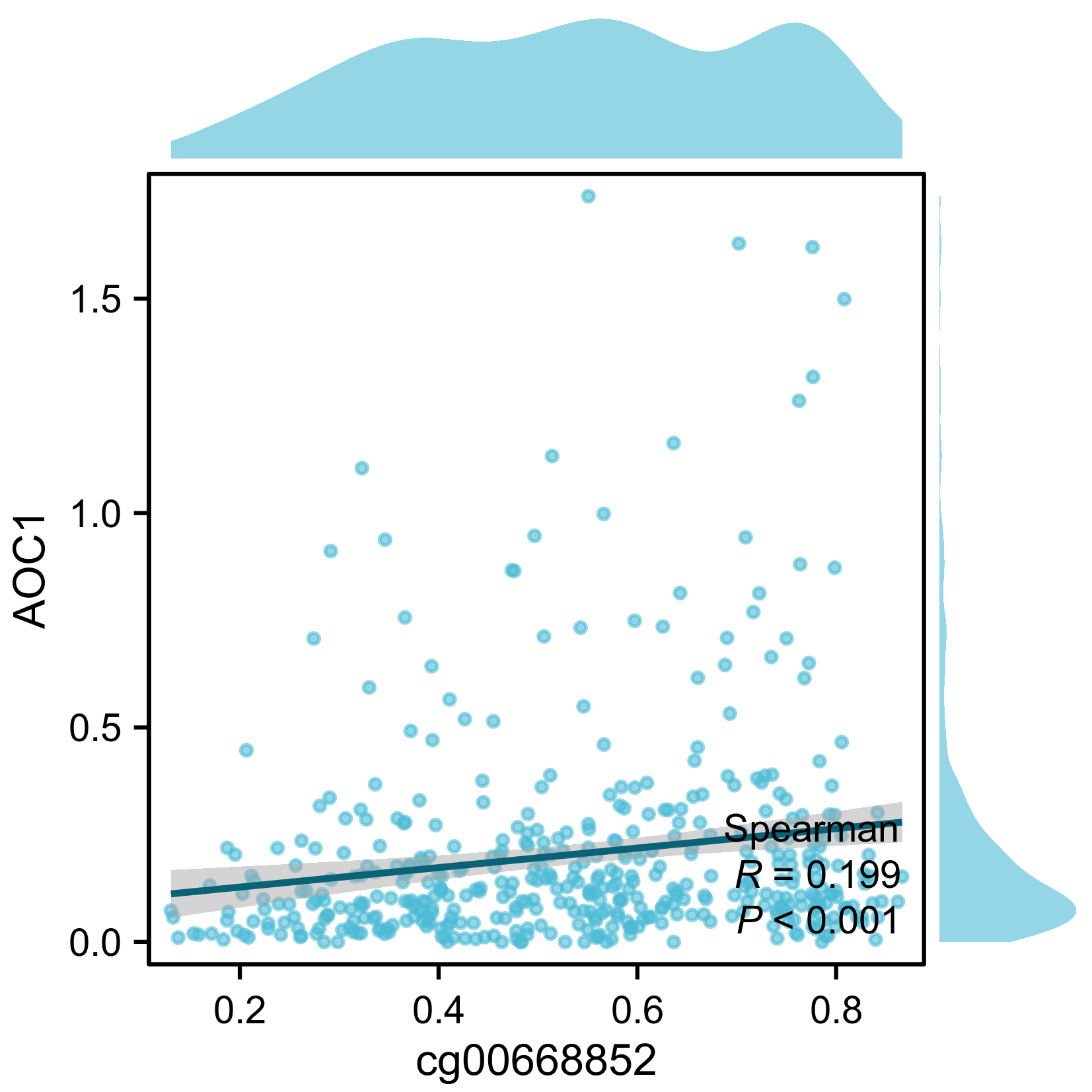

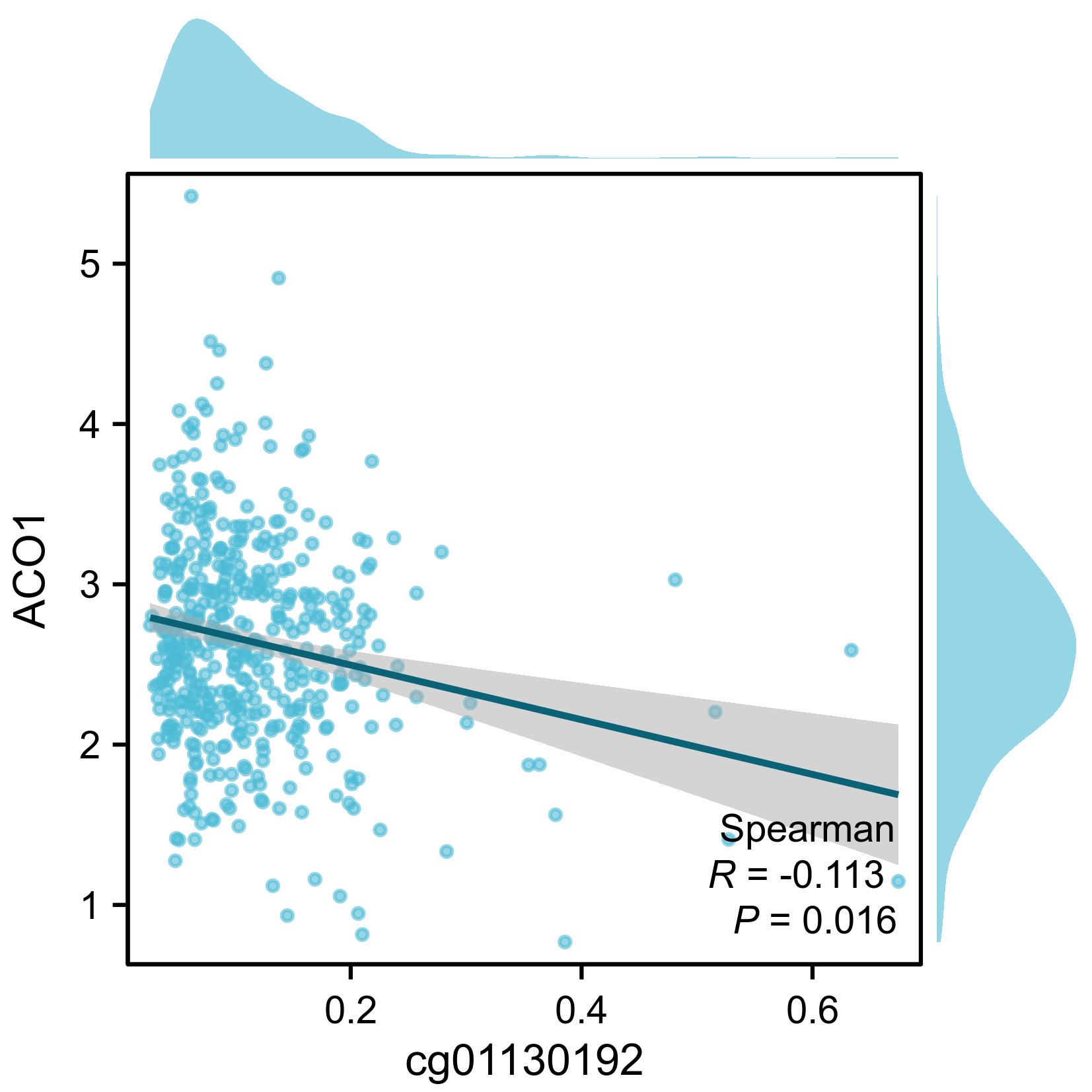

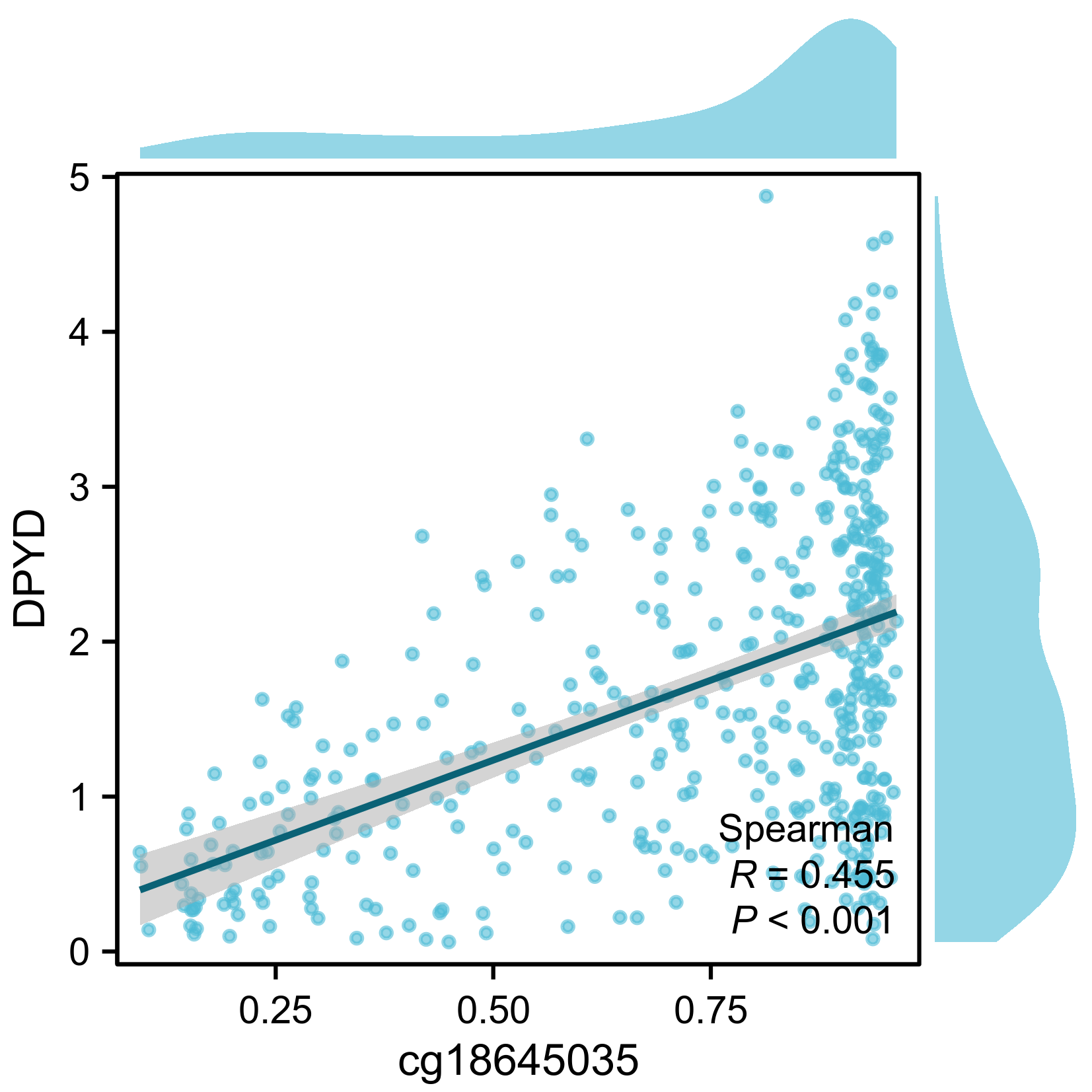

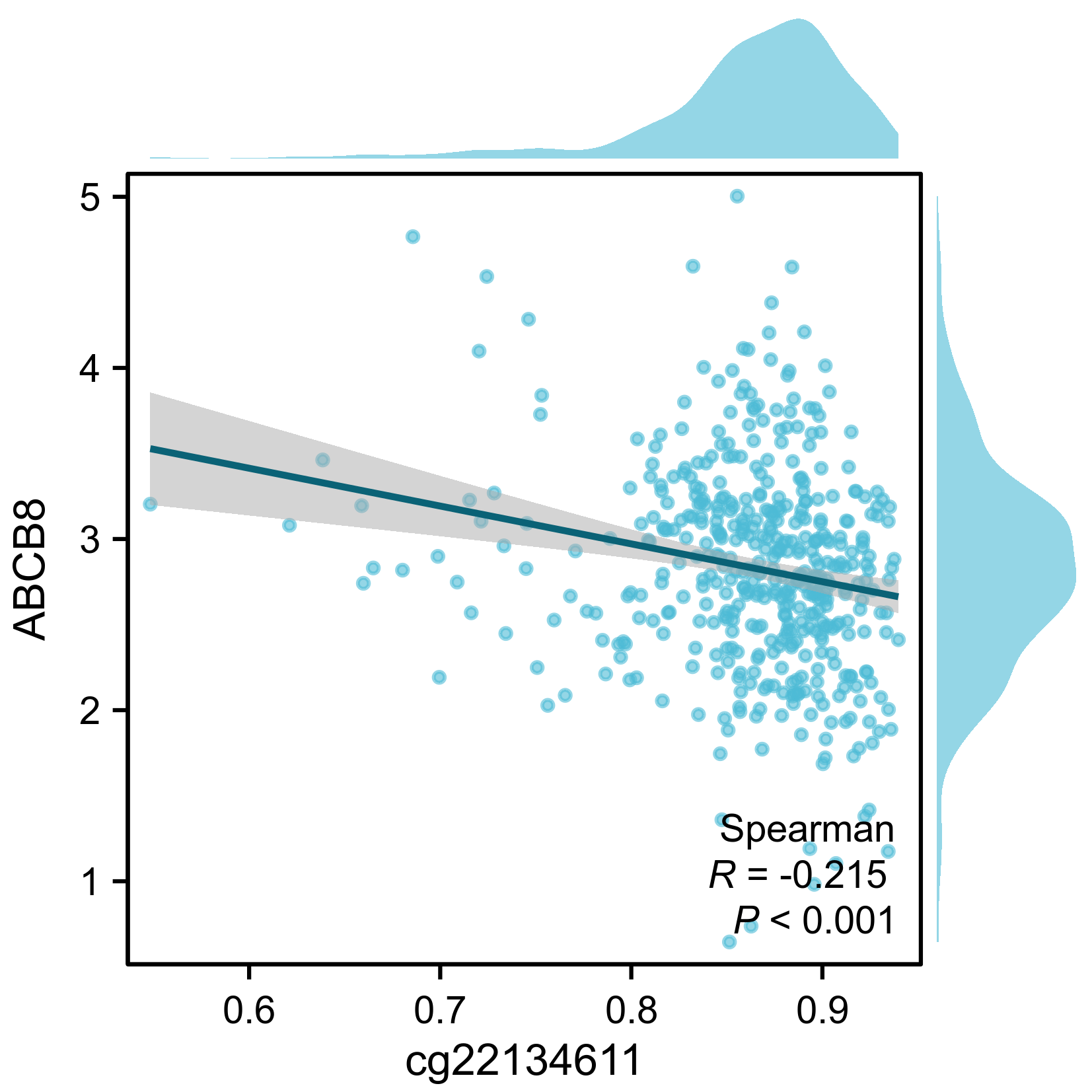

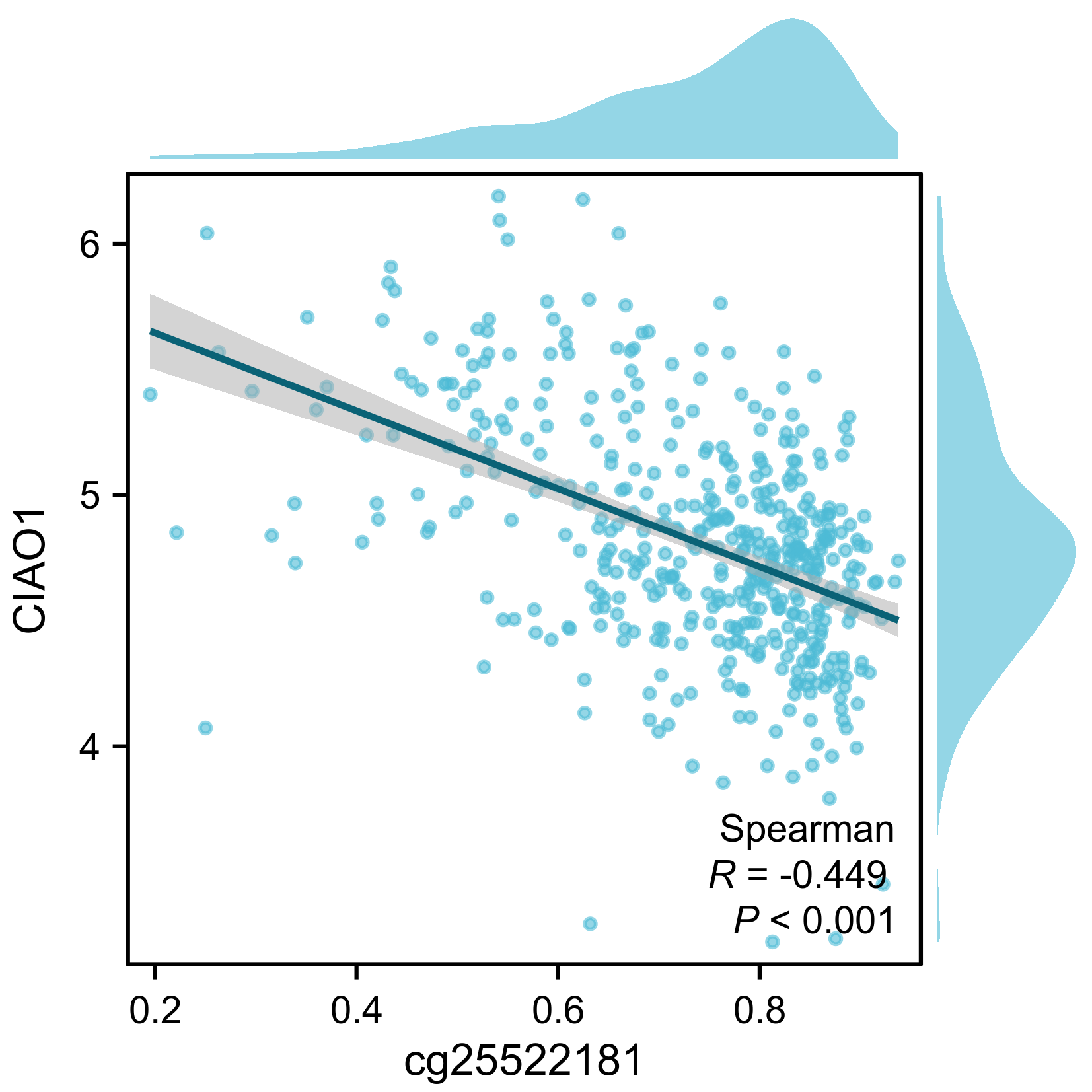


**Figure S15.** Correlation of CRG DNA methylation sites with gene expression in the TCGA cohort.


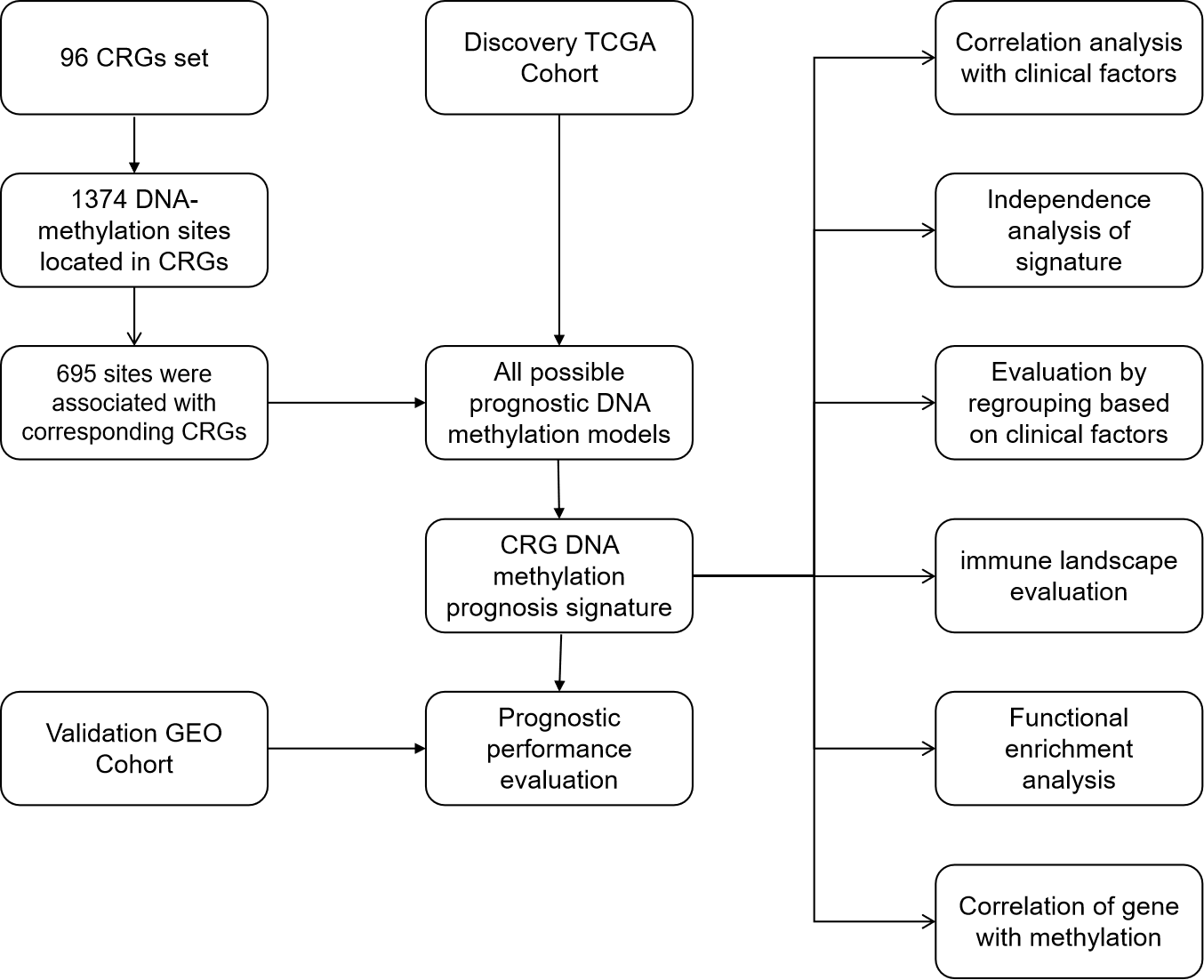


**Figure S16.** The data analysis workflow for the whole study.

**TableS1.** The list of DEGs between the high-risk and low-risk groups in the TCGA cohort.

| **ENSG00000233712** | **ENSG00000211952** | **ENSG00000235880** | **ENSG00000177455** | **ENSG00000199788** | **ENSG00000225598** | **ENSG00000111339** | **ENSG00000112486** |
| --- | --- | --- | --- | --- | --- | --- | --- |
| **ENSG00000225396** | **ENSG00000124334** | **ENSG00000271638** | **ENSG00000211789** | **ENSG00000186075** | **ENSG00000162891** | **ENSG00000211930** | **ENSG00000241451** |
| **ENSG00000189357** | **ENSG00000120659** | **ENSG00000253939** | **ENSG00000168903** | **ENSG00000264210** | **ENSG00000163207** | **ENSG00000270505** | **ENSG00000131142** |
| **ENSG00000254053** | **ENSG00000258135** | **ENSG00000246790** | **ENSG00000211804** | **ENSG00000244273** | **ENSG00000206075** | **ENSG00000255369** | **ENSG00000109684** |
| **ENSG00000254541** | **ENSG00000259276** | **ENSG00000267593** | **ENSG00000042980** | **ENSG00000253587** | **ENSG00000178934** | **ENSG00000115934** | **ENSG00000228375** |
| **ENSG00000280897** | **ENSG00000253989** | **ENSG00000232747** | **ENSG00000224034** | **ENSG00000230709** | **ENSG00000278716** | **ENSG00000179088** | **ENSG00000279127** |
| **ENSG00000207287** | **ENSG00000234895** | **ENSG00000275788** | **ENSG00000273090** | **ENSG00000252719** | **ENSG00000202380** | **ENSG00000197794** | **ENSG00000253742** |
| **ENSG00000251033** | **ENSG00000187997** | **ENSG00000273415** | **ENSG00000249820** | **ENSG00000253460** | **ENSG00000199411** | **ENSG00000254228** | **ENSG00000261030** |
| **ENSG00000227177** | **ENSG00000203756** | **ENSG00000211923** | **ENSG00000134028** | **ENSG00000281318** | **ENSG00000235382** | **ENSG00000276557** | **ENSG00000211749** |
| **ENSG00000249763** | **ENSG00000007129** | **ENSG00000201733** | **ENSG00000224049** | **ENSG00000239745** | **ENSG00000174950** | **ENSG00000264452** | **ENSG00000275158** |
| **ENSG00000237759** | **ENSG00000212568** | **ENSG00000223823** | **ENSG00000238094** | **ENSG00000258482** | **ENSG00000103067** | **ENSG00000279275** | **ENSG00000242324** |
| **ENSG00000204648** | **ENSG00000100721** | **ENSG00000263751** | **ENSG00000202222** | **ENSG00000143006** | **ENSG00000187170** | **ENSG00000212391** | **ENSG00000204670** |
| **ENSG00000280443** | **ENSG00000260997** | **ENSG00000234861** | **ENSG00000226979** | **ENSG00000253152** | **ENSG00000237138** | **ENSG00000232117** | **ENSG00000132704** |
| **ENSG00000263838** | **ENSG00000280655** | **ENSG00000230379** | **ENSG00000234534** | **ENSG00000228329** | **ENSG00000172818** | **ENSG00000211803** | **ENSG00000276602** |
| **ENSG00000273461** | **ENSG00000147234** | **ENSG00000251408** | **ENSG00000229836** | **ENSG00000174255** | **ENSG00000226156** | **ENSG00000164485** | **ENSG00000264799** |
| **ENSG00000265547** | **ENSG00000223870** | **ENSG00000211805** | **ENSG00000204475** | **ENSG00000252490** | **ENSG00000129354** | **ENSG00000253545** | **ENSG00000265612** |
| **ENSG00000211680** | **ENSG00000198821** | **ENSG00000272412** | **ENSG00000231331** | **ENSG00000229613** | **ENSG00000226145** | **ENSG00000259073** | **ENSG00000258379** |
| **ENSG00000238236** | **ENSG00000265206** | **ENSG00000116748** | **ENSG00000237604** | **ENSG00000254790** | **ENSG00000188373** | **ENSG00000221469** | **ENSG00000211806** |
| **ENSG00000252287** | **ENSG00000211625** | **ENSG00000230156** | **ENSG00000207237** | **ENSG00000249025** | **ENSG00000278131** | **ENSG00000256357** | **ENSG00000180988** |
| **ENSG00000226183** | **ENSG00000273012** | **ENSG00000211816** | **ENSG00000275210** | **ENSG00000263394** | **ENSG00000196570** | **ENSG00000226660** | **ENSG00000237286** |
| **ENSG00000281921** | **ENSG00000233928** | **ENSG00000199668** | **ENSG00000253842** | **ENSG00000211849** | **ENSG00000204538** | **ENSG00000211780** | **ENSG00000207836** |
| **ENSG00000276902** | **ENSG00000236985** | **ENSG00000211812** | **ENSG00000221887** | **ENSG00000271093** | **ENSG00000137699** | **ENSG00000276819** | **ENSG00000227196** |
| **ENSG00000277499** | **ENSG00000211827** | **ENSG00000253149** | **ENSG00000257927** | **ENSG00000253714** | **ENSG00000131264** | **ENSG00000205038** | **ENSG00000273736** |
| **ENSG00000253367** | **ENSG00000240296** | **ENSG00000230006** | **ENSG00000225899** | **ENSG00000274979** | **ENSG00000281663** | **ENSG00000259444** | **ENSG00000211879** |
| **ENSG00000248311** | **ENSG00000145681** | **ENSG00000163534** | **ENSG00000211820** | **ENSG00000229968** | **ENSG00000270779** | **ENSG00000257221** | **ENSG00000214215** |
| **ENSG00000219666** | **ENSG00000241566** | **ENSG00000211944** | **ENSG00000253653** | **ENSG00000256440** | **ENSG00000206929** | **ENSG00000255354** | **ENSG00000251163** |
| **ENSG00000238804** | **ENSG00000265118** | **ENSG00000279554** | **ENSG00000143184** | **ENSG00000231682** | **ENSG00000233502** | **ENSG00000267402** | **ENSG00000223506** |
| **ENSG00000236550** | **ENSG00000114204** | **ENSG00000264781** | **ENSG00000267731** | **ENSG00000211865** | **ENSG00000261068** | **ENSG00000239453** | **ENSG00000276759** |
| **ENSG00000254937** | **ENSG00000236345** | **ENSG00000253957** | **ENSG00000271321** | **ENSG00000251929** | **ENSG00000234942** | **ENSG00000236024** | **ENSG00000183822** |
| **ENSG00000252423** | **ENSG00000267057** | **ENSG00000211802** | **ENSG00000233515** | **ENSG00000227335** | **ENSG00000267537** | **ENSG00000227972** | **ENSG00000229313** |
| **ENSG00000274711** | **ENSG00000182487** | **ENSG00000211800** | **ENSG00000131203** | **ENSG00000242736** | **ENSG00000229505** | **ENSG00000225370** | **ENSG00000279223** |
| **ENSG00000230559** | **ENSG00000105851** | **ENSG00000249516** | **ENSG00000211771** | **ENSG00000216035** | **ENSG00000084674** | **ENSG00000279913** | **ENSG00000253666** |
| **ENSG00000258712** | **ENSG00000202125** | **ENSG00000233732** | **ENSG00000211846** | **ENSG00000235181** | **ENSG00000146910** | **ENSG00000230225** | **ENSG00000253120** |
| **ENSG00000248282** | **ENSG00000125084** | **ENSG00000139304** | **ENSG00000269667** | **ENSG00000263690** | **ENSG00000184716** | **ENSG00000260271** | **ENSG00000224605** |
| **ENSG00000211843** | **ENSG00000257599** | **ENSG00000243440** | **ENSG00000229970** | **ENSG00000211837** | **ENSG00000130701** | **ENSG00000240868** | **ENSG00000204776** |
| **ENSG00000219314** | **ENSG00000204677** | **ENSG00000255005** | **ENSG00000207835** | **ENSG00000226619** | **ENSG00000255020** | **ENSG00000228325** | **ENSG00000227090** |
| **ENSG00000201273** | **ENSG00000226075** | **ENSG00000253291** | **ENSG00000234890** | **ENSG00000235532** | **ENSG00000256577** | **ENSG00000145864** | **ENSG00000226036** |
| **ENSG00000253963** | **ENSG00000250954** | **ENSG00000205784** | **ENSG00000158485** | **ENSG00000271691** | **ENSG00000164379** | **ENSG00000256862** | **ENSG00000164483** |
| **ENSG00000229756** | **ENSG00000243012** | **ENSG00000276597** | **ENSG00000185304** | **ENSG00000244278** | **ENSG00000267879** | **ENSG00000271130** | **ENSG00000244493** |
| **ENSG00000217083** | **ENSG00000156009** | **ENSG00000234931** | **ENSG00000196092** | **ENSG00000244039** | **ENSG00000267564** | **ENSG00000278908** | **ENSG00000173208** |
| **ENSG00000278534** | **ENSG00000168702** | **ENSG00000253686** | **ENSG00000259989** | **ENSG00000224610** | **ENSG00000205076** | **ENSG00000218565** | **ENSG00000113303** |
| **ENSG00000223554** | **ENSG00000265237** | **ENSG00000270864** | **ENSG00000189350** | **ENSG00000224730** | **ENSG00000239587** | **ENSG00000270759** | **ENSG00000140835** |
| **ENSG00000252499** | **ENSG00000231105** | **ENSG00000234073** | **ENSG00000167077** | **ENSG00000262151** | **ENSG00000268307** | **ENSG00000048462** | **ENSG00000262312** |
| **ENSG00000253387** | **ENSG00000281255** | **ENSG00000124203** | **ENSG00000225206** | **ENSG00000215580** | **ENSG00000259759** | **ENSG00000248605** | **ENSG00000253590** |
| **ENSG00000255664** | **ENSG00000254704** | **ENSG00000227427** | **ENSG00000205274** | **ENSG00000226477** | **ENSG00000167759** | **ENSG00000254176** | **ENSG00000257495** |
| **ENSG00000234587** | **ENSG00000237622** | **ENSG00000266153** | **ENSG00000170486** | **ENSG00000197176** | **ENSG00000214100** | **ENSG00000211796** | **ENSG00000238241** |
| **ENSG00000232752** | **ENSG00000229131** | **ENSG00000241604** | **ENSG00000233316** | **ENSG00000257275** | **ENSG00000090512** | **ENSG00000250114** | **ENSG00000227145** |
| **ENSG00000270990** | **ENSG00000271901** | **ENSG00000120436** | **ENSG00000248138** | **ENSG00000256988** | **ENSG00000070748** | **ENSG00000136541** | **ENSG00000225107** |
| **ENSG00000199733** | **ENSG00000223608** | **ENSG00000278278** | **ENSG00000101082** | **ENSG00000237197** | **ENSG00000212176** | **ENSG00000189013** | **ENSG00000229228** |
| **ENSG00000260564** | **ENSG00000267129** | **ENSG00000122188** | **ENSG00000260341** | **ENSG00000211866** | **ENSG00000112041** | **ENSG00000253359** | **ENSG00000200455** |
| **ENSG00000211882** | **ENSG00000253519** | **ENSG00000211728** | **ENSG00000233828** | **ENSG00000236597** | **ENSG00000253288** | **ENSG00000242258** | **ENSG00000211914** |
| **ENSG00000220076** | **ENSG00000249463** | **ENSG00000211813** | **ENSG00000140284** | **ENSG00000260719** | **ENSG00000274139** | **ENSG00000251393** | **ENSG00000254036** |
| **ENSG00000260303** | **ENSG00000237385** | **ENSG00000260496** | **ENSG00000211764** | **ENSG00000256582** | **ENSG00000231863** | **ENSG00000237470** | **ENSG00000124196** |
| **ENSG00000261637** | **ENSG00000281327** | **ENSG00000178965** | **ENSG00000150637** | **ENSG00000226757** | **ENSG00000212392** | **ENSG00000178199** | **ENSG00000234777** |
| **ENSG00000277669** | **ENSG00000211701** | **ENSG00000253242** | **ENSG00000234758** | **ENSG00000225741** | **ENSG00000254933** | **ENSG00000196329** | **ENSG00000211863** |
| **ENSG00000253732** | **ENSG00000237404** | **ENSG00000236703** | **ENSG00000101292** | **ENSG00000244071** | **ENSG00000250504** | **ENSG00000267554** | **ENSG00000198798** |
| **ENSG00000216265** | **ENSG00000228714** | **ENSG00000211626** | **ENSG00000125735** | **ENSG00000279942** | **ENSG00000249430** | **ENSG00000211785** | **ENSG00000251419** |
| **ENSG00000234580** | **ENSG00000223552** | **ENSG00000240306** | **ENSG00000269050** | **ENSG00000245466** | **ENSG00000236292** | **ENSG00000221535** | **ENSG00000222017** |
| **ENSG00000234540** | **ENSG00000279617** | **ENSG00000230231** | **ENSG00000270443** | **ENSG00000253690** | **ENSG00000052344** | **ENSG00000253131** | **ENSG00000266216** |
| **ENSG00000229205** | **ENSG00000250829** | **ENSG00000259261** | **ENSG00000240671** | **ENSG00000228131** | **ENSG00000165953** | **ENSG00000251922** | **ENSG00000229311** |
| **ENSG00000253870** | **ENSG00000205837** | **ENSG00000188282** | **ENSG00000121570** | **ENSG00000271015** | **ENSG00000182040** | **ENSG00000253891** | **ENSG00000260976** |
| **ENSG00000254215** | **ENSG00000226047** | **ENSG00000256553** | **ENSG00000226012** | **ENSG00000223466** | **ENSG00000231645** | **ENSG00000248898** | **ENSG00000263413** |
| **ENSG00000249036** | **ENSG00000236213** | **ENSG00000139610** | **ENSG00000236452** | **ENSG00000271620** | **ENSG00000224109** | **ENSG00000179840** | **ENSG00000211715** |
| **ENSG00000239628** | **ENSG00000276003** | **ENSG00000239975** | **ENSG00000151418** | **ENSG00000211870** | **ENSG00000230088** | **ENSG00000074706** | **ENSG00000248510** |
| **ENSG00000253790** | **ENSG00000224137** | **ENSG00000256659** | **ENSG00000182393** | **ENSG00000271523** | **ENSG00000266861** | **ENSG00000280003** | **ENSG00000259680** |
| **ENSG00000211832** | **ENSG00000257777** | **ENSG00000264773** | **ENSG00000225509** | **ENSG00000280015** | **ENSG00000237571** | **ENSG00000274172** | **ENSG00000258879** |
| **ENSG00000255528** | **ENSG00000205809** | **ENSG00000270966** | **ENSG00000147183** | **ENSG00000104970** | **ENSG00000259446** | **ENSG00000256166** | **ENSG00000123594** |
| **ENSG00000265108** | **ENSG00000211665** | **ENSG00000257191** | **ENSG00000264344** | **ENSG00000252339** | **ENSG00000252119** | **ENSG00000237346** | **ENSG00000267712** |
| **ENSG00000223490** | **ENSG00000152784** | **ENSG00000276699** | **ENSG00000227678** | **ENSG00000232381** | **ENSG00000277619** | **ENSG00000281203** | **ENSG00000211931** |
| **ENSG00000273288** | **ENSG00000180090** | **ENSG00000267074** | **ENSG00000198178** | **ENSG00000187754** | **ENSG00000154227** | **ENSG00000204983** | **ENSG00000269391** |
| **ENSG00000253759** | **ENSG00000251169** | **ENSG00000258511** | **ENSG00000280137** | **ENSG00000253822** | **ENSG00000279459** | **ENSG00000238160** | **ENSG00000250850** |
| **ENSG00000184814** | **ENSG00000236256** | **ENSG00000239744** | **ENSG00000263835** | **ENSG00000264174** | **ENSG00000213210** | **ENSG00000248962** | **ENSG00000227066** |
| **ENSG00000221686** | **ENSG00000274576** | **ENSG00000145850** | **ENSG00000264609** | **ENSG00000280298** | **ENSG00000184502** | **ENSG00000143167** | **ENSG00000255750** |
| **ENSG00000278584** | **ENSG00000254140** | **ENSG00000253479** | **ENSG00000163217** | **ENSG00000225271** | **ENSG00000187821** | **ENSG00000167634** | **ENSG00000260834** |
| **ENSG00000265243** | **ENSG00000228668** | **ENSG00000253823** | **ENSG00000220517** | **ENSG00000271046** | **ENSG00000186212** | **ENSG00000254686** | **ENSG00000270318** |
| **ENSG00000277297** | **ENSG00000256913** | **ENSG00000223662** | **ENSG00000257193** | **ENSG00000254329** | **ENSG00000063515** | **ENSG00000254240** | **ENSG00000260015** |
| **ENSG00000271257** | **ENSG00000202569** | **ENSG00000268027** | **ENSG00000172215** | **ENSG00000234174** | **ENSG00000258215** | **ENSG00000229972** | **ENSG00000223929** |
| **ENSG00000260525** | **ENSG00000254395** | **ENSG00000234332** | **ENSG00000219088** | **ENSG00000259186** | **ENSG00000239211** | **ENSG00000271065** | **ENSG00000236946** |
| **ENSG00000228943** | **ENSG00000233834** | **ENSG00000237372** | **ENSG00000184140** | **ENSG00000211888** | **ENSG00000225950** | **ENSG00000228600** | **ENSG00000272917** |
| **ENSG00000207172** | **ENSG00000259436** | **ENSG00000255569** | **ENSG00000233473** | **ENSG00000223816** | **ENSG00000201839** | **ENSG00000262090** | **ENSG00000259922** |
| **ENSG00000265822** | **ENSG00000204382** | **ENSG00000274134** | **ENSG00000279741** | **ENSG00000261289** | **ENSG00000176194** | **ENSG00000240954** | **ENSG00000183185** |
| **ENSG00000253491** | **ENSG00000228341** | **ENSG00000261644** | **ENSG00000228759** | **ENSG00000233655** | **ENSG00000261268** | **ENSG00000237254** | **ENSG00000201517** |
| **ENSG00000280082** | **ENSG00000218357** | **ENSG00000217643** | **ENSG00000227155** | **ENSG00000278975** | **ENSG00000257500** | **ENSG00000275747** | **ENSG00000201343** |
| **ENSG00000238447** | **ENSG00000030419** | **ENSG00000233306** | **ENSG00000172116** | **ENSG00000227421** | **ENSG00000254967** | **ENSG00000278857** | **ENSG00000276756** |
| **ENSG00000252204** | **ENSG00000144191** | **ENSG00000226254** | **ENSG00000180539** | **ENSG00000105205** | **ENSG00000196805** | **ENSG00000261416** | **ENSG00000267670** |
| **ENSG00000256835** | **ENSG00000278678** | **ENSG00000265517** | **ENSG00000271010** | **ENSG00000281372** | **ENSG00000057149** | **ENSG00000233999** | **ENSG00000211851** |
| **ENSG00000252626** | **ENSG00000226233** | **ENSG00000242534** | **ENSG00000154451** | **ENSG00000214759** | **ENSG00000226792** | **ENSG00000211788** | **ENSG00000265589** |
| **ENSG00000222356** | **ENSG00000280914** | **ENSG00000224107** | **ENSG00000237513** | **ENSG00000236525** | **ENSG00000250456** | **ENSG00000211911** | **ENSG00000274773** |
| **ENSG00000253635** | **ENSG00000235831** | **ENSG00000260516** | **ENSG00000243398** | **ENSG00000200702** | **ENSG00000265554** | **ENSG00000251546** | **ENSG00000274880** |
| **ENSG00000266981** | **ENSG00000231020** | **ENSG00000095627** | **ENSG00000249226** | **ENSG00000275361** | **ENSG00000241131** | **ENSG00000182896** | **ENSG00000236790** |
| **ENSG00000276050** | **ENSG00000274256** | **ENSG00000254157** | **ENSG00000260145** | **ENSG00000279775** | **ENSG00000229526** | **ENSG00000227619** | **ENSG00000267257** |
| **ENSG00000181698** | **ENSG00000224478** | **ENSG00000267046** | **ENSG00000250721** | **ENSG00000249454** | **ENSG00000234056** | **ENSG00000232208** | **ENSG00000006659** |
| **ENSG00000261249** | **ENSG00000140030** | **ENSG00000224609** | **ENSG00000065609** | **ENSG00000261834** | **ENSG00000235054** | **ENSG00000240787** | **ENSG00000211873** |
| **ENSG00000151033** | **ENSG00000223534** | **ENSG00000249334** | **ENSG00000279296** | **ENSG00000181211** | **ENSG00000165553** | **ENSG00000213140** | **ENSG00000275046** |
| **ENSG00000175143** | **ENSG00000168229** | **ENSG00000233665** | **ENSG00000236853** | **ENSG00000239736** | **ENSG00000199350** | **ENSG00000234736** | **ENSG00000225079** |
| **ENSG00000204754** | **ENSG00000258860** | **ENSG00000147138** | **ENSG00000211938** | **ENSG00000244158** | **ENSG00000120471** | **ENSG00000200269** | **ENSG00000160856** |
| **ENSG00000250688** | **ENSG00000228512** | **ENSG00000211778** | **ENSG00000089012** | **ENSG00000165584** | **ENSG00000237159** | **ENSG00000273348** | **ENSG00000258407** |
| **ENSG00000226122** | **ENSG00000211599** | **ENSG00000174946** | **ENSG00000187627** | **ENSG00000280002** | **ENSG00000275216** | **ENSG00000276471** | **ENSG00000226423** |
| **ENSG00000242348** | **ENSG00000243063** | **ENSG00000235113** | **ENSG00000179813** | **ENSG00000164500** | **ENSG00000254592** | **ENSG00000117560** | **ENSG00000281905** |
| **ENSG00000239679** | **ENSG00000274591** | **ENSG00000232359** | **ENSG00000228139** | **ENSG00000201695** | **ENSG00000243872** | **ENSG00000236077** | **ENSG00000199370** |
| **ENSG00000273962** | **ENSG00000132464** | **ENSG00000221519** | **ENSG00000237300** | **ENSG00000197888** | **ENSG00000159516** | **ENSG00000229757** | **ENSG00000221043** |
| **ENSG00000211781** | **ENSG00000211657** | **ENSG00000211833** | **ENSG00000009790** | **ENSG00000270748** | **ENSG00000203857** | **ENSG00000226933** | **ENSG00000166211** |
| **ENSG00000211878** | **ENSG00000167208** | **ENSG00000231265** | **ENSG00000279727** | **ENSG00000251643** | **ENSG00000153404** | **ENSG00000183625** | **ENSG00000111732** |
| **ENSG00000231743** | **ENSG00000215124** | **ENSG00000175779** | **ENSG00000161405** | **ENSG00000276454** | **ENSG00000049283** | **ENSG00000241657** | **ENSG00000231859** |
| **ENSG00000236689** | **ENSG00000260228** | **ENSG00000266908** | **ENSG00000253796** | **ENSG00000252421** | **ENSG00000268654** | **ENSG00000211921** | **ENSG00000224521** |
| **ENSG00000211739** | **ENSG00000174776** | **ENSG00000211814** | **ENSG00000211707** | **ENSG00000211842** | **ENSG00000260969** | **ENSG00000211656** | **ENSG00000218730** |
| **ENSG00000253588** | **ENSG00000237272** | **ENSG00000211821** | **ENSG00000231090** | **ENSG00000234622** | **ENSG00000186529** | **ENSG00000147570** | **ENSG00000237352** |
| **ENSG00000274493** | **ENSG00000234919** | **ENSG00000181109** | **ENSG00000237434** | **ENSG00000253241** | **ENSG00000231228** | **ENSG00000236504** | **ENSG00000229673** |
| **ENSG00000232361** | **ENSG00000280011** | **ENSG00000225783** | **ENSG00000113263** | **ENSG00000202264** | **ENSG00000236396** | **ENSG00000211795** | **ENSG00000265356** |
| **ENSG00000228688** | **ENSG00000256041** | **ENSG00000248991** | **ENSG00000251934** | **ENSG00000270187** | **ENSG00000162344** | **ENSG00000233261** | **ENSG00000197880** |
| **ENSG00000253310** | **ENSG00000256917** | **ENSG00000253409** | **ENSG00000227802** | **ENSG00000275772** | **ENSG00000206909** | **ENSG00000221476** | **ENSG00000252757** |
| **ENSG00000238002** | **ENSG00000105501** | **ENSG00000225541** | **ENSG00000143185** | **ENSG00000226921** | **ENSG00000108417** | **ENSG00000121895** | **ENSG00000224585** |
| **ENSG00000265964** | **ENSG00000138378** | **ENSG00000258760** | **ENSG00000228590** | **ENSG00000253924** | **ENSG00000169509** | **ENSG00000231131** | **ENSG00000245954** |
| **ENSG00000211875** | **ENSG00000259078** | **ENSG00000125900** | **ENSG00000204872** | **ENSG00000253703** | **ENSG00000259457** | **ENSG00000173626** | **ENSG00000260929** |
| **ENSG00000238660** | **ENSG00000278095** | **ENSG00000181803** | **ENSG00000226968** | **ENSG00000243305** | **ENSG00000197641** | **ENSG00000280637** | **ENSG00000249867** |
| **ENSG00000257629** | **ENSG00000253497** | **ENSG00000264024** | **ENSG00000211667** | **ENSG00000197057** | **ENSG00000236770** | **ENSG00000183918** | **ENSG00000152969** |
| **ENSG00000211872** | **ENSG00000249639** | **ENSG00000234568** | **ENSG00000218153** | **ENSG00000189233** | **ENSG00000223784** | **ENSG00000188263** | **ENSG00000235304** |
| **ENSG00000259097** | **ENSG00000196209** | **ENSG00000213809** | **ENSG00000224557** | **ENSG00000213604** | **ENSG00000250820** | **ENSG00000223931** | **ENSG00000211880** |
| **ENSG00000201612** | **ENSG00000254260** | **ENSG00000232542** | **ENSG00000273632** | **ENSG00000188011** | **ENSG00000171711** | **ENSG00000255001** | **ENSG00000249960** |
| **ENSG00000249041** | **ENSG00000211972** | **ENSG00000211694** | **ENSG00000112195** | **ENSG00000223750** | **ENSG00000279271** | **ENSG00000204110** | **ENSG00000263417** |
| **ENSG00000224187** | **ENSG00000243290** | **ENSG00000224875** | **ENSG00000264186** | **ENSG00000171136** | **ENSG00000206177** | **ENSG00000227191** | **ENSG00000236856** |
| **ENSG00000251159** | **ENSG00000200070** | **ENSG00000213262** | **ENSG00000278030** | **ENSG00000214797** | **ENSG00000263639** | **ENSG00000251011** | **ENSG00000211920** |
| **ENSG00000204001** | **ENSG00000257065** | **ENSG00000211883** | **ENSG00000257156** | **ENSG00000234663** | **ENSG00000108759** | **ENSG00000266637** | **ENSG00000187510** |
| **ENSG00000248667** | **ENSG00000241678** | **ENSG00000256568** | **ENSG00000279886** | **ENSG00000229769** | **ENSG00000250561** | **ENSG00000234142** | **ENSG00000259092** |
| **ENSG00000234452** | **ENSG00000183840** | **ENSG00000261218** | **ENSG00000231023** | **ENSG00000207782** | **ENSG00000234787** | **ENSG00000259084** | **ENSG00000254056** |
| **ENSG00000211862** | **ENSG00000265531** | **ENSG00000172362** | **ENSG00000244142** | **ENSG00000276362** | **ENSG00000186226** | **ENSG00000280954** | **ENSG00000279370** |
| **ENSG00000264906** | **ENSG00000179073** | **ENSG00000162897** | **ENSG00000253338** | **ENSG00000281041** | **ENSG00000205325** | **ENSG00000251009** | **ENSG00000187037** |
| **ENSG00000258869** | **ENSG00000229474** | **ENSG00000236935** | **ENSG00000162739** | **ENSG00000263989** | **ENSG00000213606** | **ENSG00000250447** | **ENSG00000259852** |
| **ENSG00000252650** | **ENSG00000225366** | **ENSG00000236009** | **ENSG00000237926** | **ENSG00000250026** | **ENSG00000167757** | **ENSG00000265929** | **ENSG00000184735** |
| **ENSG00000265423** | **ENSG00000159753** | **ENSG00000233093** | **ENSG00000136573** | **ENSG00000271288** | **ENSG00000224984** | **ENSG00000249993** | **ENSG00000196684** |
| **ENSG00000259997** | **ENSG00000161929** | **ENSG00000223631** | **ENSG00000198019** | **ENSG00000227170** | **ENSG00000204175** | **ENSG00000237567** | **ENSG00000171049** |
| **ENSG00000211681** | **ENSG00000104921** | **ENSG00000211854** | **ENSG00000236866** | **ENSG00000271600** | **ENSG00000240519** | **ENSG00000188848** | **ENSG00000253271** |
| **ENSG00000273894** | **ENSG00000244414** | **ENSG00000201342** | **ENSG00000172901** | **ENSG00000250719** | **ENSG00000166828** | **ENSG00000241560** | **ENSG00000134242** |
| **ENSG00000251376** | **ENSG00000238266** | **ENSG00000253763** | **ENSG00000211933** | **ENSG00000253209** | **ENSG00000260230** | **ENSG00000211776** | **ENSG00000183395** |
| **ENSG00000233193** | **ENSG00000186583** | **ENSG00000232613** | **ENSG00000182230** | **ENSG00000258842** | **ENSG00000176075** | **ENSG00000179841** | **ENSG00000260484** |
| **ENSG00000254275** | **ENSG00000253701** | **ENSG00000153789** | **ENSG00000255693** | **ENSG00000211868** | **ENSG00000280335** | **ENSG00000248647** | **ENSG00000180105** |
| **ENSG00000248170** | **ENSG00000240767** | **ENSG00000214686** | **ENSG00000196171** | **ENSG00000207349** | **ENSG00000214822** | **ENSG00000268804** | **ENSG00000101916** |
| **ENSG00000222086** | **ENSG00000255353** | **ENSG00000215177** | **ENSG00000272908** | **ENSG00000259496** | **ENSG00000168907** | **ENSG00000253239** | **ENSG00000265879** |
| **ENSG00000248990** | **ENSG00000104972** | **ENSG00000248672** | **ENSG00000163554** | **ENSG00000244104** | **ENSG00000186844** | **ENSG00000279805** | **ENSG00000249849** |
| **ENSG00000248571** | **ENSG00000264488** | **ENSG00000265070** | **ENSG00000125245** | **ENSG00000177590** | **ENSG00000269855** | **ENSG00000121594** | **ENSG00000210181** |
| **ENSG00000254097** | **ENSG00000184029** | **ENSG00000221492** | **ENSG00000206531** | **ENSG00000279082** | **ENSG00000261150** | **ENSG00000211633** | **ENSG00000107447** |
| **ENSG00000211864** | **ENSG00000232109** | **ENSG00000205846** | **ENSG00000231772** | **ENSG00000232578** | **ENSG00000276521** | **ENSG00000270252** | **ENSG00000211924** |
| **ENSG00000227706** | **ENSG00000168685** | **ENSG00000280348** | **ENSG00000272274** | **ENSG00000268861** | **ENSG00000184945** | **ENSG00000259834** | **ENSG00000166329** |
| **ENSG00000236864** | **ENSG00000279379** | **ENSG00000271582** | **ENSG00000229922** | **ENSG00000225948** | **ENSG00000272050** | **ENSG00000201347** | **ENSG00000264792** |
| **ENSG00000248717** | **ENSG00000249742** | **ENSG00000240505** | **ENSG00000266804** | **ENSG00000255054** | **ENSG00000229544** | **ENSG00000271680** | **ENSG00000182183** |
| **ENSG00000223158** | **ENSG00000236481** | **ENSG00000265714** | **ENSG00000232557** | **ENSG00000261704** | **ENSG00000125998** | **ENSG00000268500** | **ENSG00000211974** |
| **ENSG00000274037** | **ENSG00000187715** | **ENSG00000225269** | **ENSG00000274008** | **ENSG00000211877** | **ENSG00000233670** | **ENSG00000240143** | **ENSG00000258352** |
| **ENSG00000249054** | **ENSG00000211747** | **ENSG00000267654** | **ENSG00000249978** | **ENSG00000179253** | **ENSG00000113430** | **ENSG00000211794** | **ENSG00000259309** |
| **ENSG00000238909** | **ENSG00000243469** | **ENSG00000259490** | **ENSG00000258763** | **ENSG00000164270** | **ENSG00000167080** | **ENSG00000211793** | **ENSG00000236278** |
| **ENSG00000201317** | **ENSG00000230109** | **ENSG00000226298** | **ENSG00000253415** | **ENSG00000236049** | **ENSG00000205678** | **ENSG00000249881** | **ENSG00000269998** |
| **ENSG00000236170** | **ENSG00000221962** | **ENSG00000249096** | **ENSG00000277030** | **ENSG00000200571** | **ENSG00000102243** | **ENSG00000211790** | **ENSG00000211698** |
| **ENSG00000227217** | **ENSG00000234564** | **ENSG00000176566** | **ENSG00000257226** | **ENSG00000187550** | **ENSG00000166869** | **ENSG00000240040** | **ENSG00000237111** |
| **ENSG00000269460** | **ENSG00000185811** | **ENSG00000260990** | **ENSG00000171643** | **ENSG00000226004** | **ENSG00000261092** | **ENSG00000249977** | **ENSG00000076662** |
| **ENSG00000236800** | **ENSG00000244429** | **ENSG00000173585** | **ENSG00000092345** | **ENSG00000211720** | **ENSG00000273760** | **ENSG00000232271** | **ENSG00000251215** |
| **ENSG00000201070** | **ENSG00000225690** | **ENSG00000174123** | **ENSG00000274295** | **ENSG00000225885** | **ENSG00000273252** | **ENSG00000211734** | **ENSG00000213489** |
| **ENSG00000226153** | **ENSG00000228166** | **ENSG00000277096** | **ENSG00000166866** | **ENSG00000253191** | **ENSG00000235357** | **ENSG00000280008** | **ENSG00000258875** |
| **ENSG00000253980** | **ENSG00000243417** | **ENSG00000238025** | **ENSG00000137080** | **ENSG00000211674** | **ENSG00000240152** | **ENSG00000117215** | **ENSG00000259946** |
| **ENSG00000228167** | **ENSG00000232949** | **ENSG00000276405** | **ENSG00000006116** | **ENSG00000222042** | **ENSG00000263571** | **ENSG00000236303** | **ENSG00000238077** |
| **ENSG00000257906** | **ENSG00000253325** | **ENSG00000211777** | **ENSG00000225101** | **ENSG00000266976** | **ENSG00000253124** | **ENSG00000230099** | **ENSG00000054219** |
| **ENSG00000239008** | **ENSG00000211664** | **ENSG00000277282** | **ENSG00000258196** | **ENSG00000255733** | **ENSG00000227721** | **ENSG00000211710** | **ENSG00000086288** |
| **ENSG00000279914** | **ENSG00000256590** | **ENSG00000272053** | **ENSG00000266378** | **ENSG00000225548** | **ENSG00000243905** | **ENSG00000275743** | **ENSG00000227765** |
| **ENSG00000253941** | **ENSG00000146666** | **ENSG00000211727** | **ENSG00000237775** | **ENSG00000211623** | **ENSG00000267629** | **ENSG00000243238** | **ENSG00000127074** |
| **ENSG00000274223** | **ENSG00000205358** | **ENSG00000151577** | **ENSG00000242009** | **ENSG00000254287** | **ENSG00000204889** | **ENSG00000240766** | **ENSG00000183813** |
| **ENSG00000232512** | **ENSG00000226032** | **ENSG00000250310** | **ENSG00000211942** | **ENSG00000248767** | **ENSG00000264144** | **ENSG00000205436** | **ENSG00000253441** |
| **ENSG00000268618** | **ENSG00000263787** | **ENSG00000211695** | **ENSG00000232166** | **ENSG00000237638** | **ENSG00000168267** | **ENSG00000205044** | **ENSG00000270835** |
| **ENSG00000249689** | **ENSG00000268758** | **ENSG00000261182** | **ENSG00000261774** | **ENSG00000231202** | **ENSG00000075673** | **ENSG00000205537** | **ENSG00000261018** |
| **ENSG00000211682** | **ENSG00000233318** | **ENSG00000231621** | **ENSG00000168356** | **ENSG00000248227** | **ENSG00000260765** | **ENSG00000218300** | **ENSG00000253936** |
| **ENSG00000263926** | **ENSG00000181631** | **ENSG00000264254** | **ENSG00000108405** | **ENSG00000250715** | **ENSG00000156150** | **ENSG00000231674** | **ENSG00000231216** |
| **ENSG00000201157** | **ENSG00000261257** | **ENSG00000221356** | **ENSG00000259907** | **ENSG00000182531** | **ENSG00000275061** | **ENSG00000251309** | **ENSG00000226806** |
| **ENSG00000201231** | **ENSG00000204293** | **ENSG00000229088** | **ENSG00000230322** | **ENSG00000211717** | **ENSG00000251410** | **ENSG00000270474** | **ENSG00000233470** |
| **ENSG00000222344** | **ENSG00000133477** | **ENSG00000237747** | **ENSG00000105428** | **ENSG00000254167** | **ENSG00000178243** | **ENSG00000278234** | **ENSG00000230533** |
| **ENSG00000207002** | **ENSG00000259725** | **ENSG00000237593** | **ENSG00000255299** | **ENSG00000211768** | **ENSG00000167754** | **ENSG00000137757** | **ENSG00000244116** |
| **ENSG00000217268** | **ENSG00000180458** | **ENSG00000267349** | **ENSG00000121807** | **ENSG00000188676** | **ENSG00000230937** | **ENSG00000235221** | **ENSG00000177494** |
| **ENSG00000237914** | **ENSG00000259463** | **ENSG00000255987** | **ENSG00000256474** | **ENSG00000174171** | **ENSG00000279844** | **ENSG00000179934** | **ENSG00000255437** |
| **ENSG00000229559** | **ENSG00000129437** | **ENSG00000234184** | **ENSG00000254760** | **ENSG00000207201** | **ENSG00000075461** | **ENSG00000200235** | **ENSG00000230537** |
| **ENSG00000280024** | **ENSG00000277624** | **ENSG00000259028** | **ENSG00000163606** | **ENSG00000260861** | **ENSG00000152931** | **ENSG00000259337** | **ENSG00000238015** |
| **ENSG00000275012** | **ENSG00000231004** | **ENSG00000186152** | **ENSG00000258705** | **ENSG00000233355** | **ENSG00000129951** | **ENSG00000211752** | **ENSG00000188403** |
| **ENSG00000250640** | **ENSG00000064270** | **ENSG00000229255** | **ENSG00000211979** | **ENSG00000261435** | **ENSG00000163202** | **ENSG00000211746** | **ENSG00000105967** |
| **ENSG00000279906** | **ENSG00000256879** | **ENSG00000256948** | **ENSG00000196119** | **ENSG00000252348** | **ENSG00000147689** | **ENSG00000181036** | **ENSG00000247193** |
| **ENSG00000258878** | **ENSG00000169469** | **ENSG00000188822** | **ENSG00000227730** | **ENSG00000197476** | **ENSG00000223948** | **ENSG00000277402** | **ENSG00000211801** |
| **ENSG00000279201** | **ENSG00000207242** | **ENSG00000224791** | **ENSG00000231858** | **ENSG00000236320** | **ENSG00000206073** | **ENSG00000211676** | **ENSG00000170128** |
| **ENSG00000198601** | **ENSG00000143556** | **ENSG00000275791** | **ENSG00000228800** | **ENSG00000101746** | **ENSG00000145934** | **ENSG00000211848** | **ENSG00000224611** |
| **ENSG00000280250** | **ENSG00000269356** | **ENSG00000230481** | **ENSG00000113088** | **ENSG00000269885** | **ENSG00000162040** | **ENSG00000135426** | **ENSG00000177294** |
| **ENSG00000171180** | **ENSG00000280743** | **ENSG00000262823** | **ENSG00000239819** | **ENSG00000281179** | **ENSG00000109101** | **ENSG00000211871** | **ENSG00000237153** |
| **ENSG00000265627** | **ENSG00000130700** | **ENSG00000278195** | **ENSG00000278473** | **ENSG00000234484** | **ENSG00000264230** | **ENSG00000229563** | **ENSG00000224410** |
| **ENSG00000270900** | **ENSG00000225823** | **ENSG00000244661** | **ENSG00000224739** | **ENSG00000273445** | **ENSG00000249727** | **ENSG00000256518** | **ENSG00000202268** |
| **ENSG00000250894** | **ENSG00000200254** | **ENSG00000245869** | **ENSG00000166984** | **ENSG00000170953** | **ENSG00000261320** | **ENSG00000253274** | **ENSG00000229754** |
| **ENSG00000278537** | **ENSG00000169174** | **ENSG00000242371** | **ENSG00000256706** | **ENSG00000217482** | **ENSG00000232394** | **ENSG00000215267** | **ENSG00000011590** |
| **ENSG00000279992** | **ENSG00000267000** | **ENSG00000225720** | **ENSG00000243532** | **ENSG00000253435** | **ENSG00000227300** | **ENSG00000229106** | **ENSG00000254957** |
| **ENSG00000146399** | **ENSG00000250066** | **ENSG00000243961** | **ENSG00000188389** | **ENSG00000250507** | **ENSG00000214305** | **ENSG00000237265** | **ENSG00000250541** |
| **ENSG00000219722** | **ENSG00000140600** | **ENSG00000276953** | **ENSG00000272218** | **ENSG00000239571** | **ENSG00000101180** | **ENSG00000269220** | **ENSG00000079263** |
| **ENSG00000274061** | **ENSG00000127129** | **ENSG00000271225** | **ENSG00000185888** | **ENSG00000259703** | **ENSG00000186474** | **ENSG00000235550** | **ENSG00000197549** |
| **ENSG00000248107** | **ENSG00000186009** | **ENSG00000258826** | **ENSG00000211688** | **ENSG00000226571** | **ENSG00000188089** | **ENSG00000248702** | **ENSG00000225964** |
| **ENSG00000239397** | **ENSG00000100170** | **ENSG00000228403** | **ENSG00000242076** | **ENSG00000267035** | **ENSG00000261437** | **ENSG00000187033** | **ENSG00000102794** |
| **ENSG00000211847** | **ENSG00000205015** | **ENSG00000271375** | **ENSG00000260234** | **ENSG00000259641** | **ENSG00000265190** | **ENSG00000163600** | **ENSG00000253364** |
| **ENSG00000264494** | **ENSG00000266521** | **ENSG00000271178** | **ENSG00000271035** | **ENSG00000211499** | **ENSG00000125850** | **ENSG00000164821** | **ENSG00000211766** |
| **ENSG00000254264** | **ENSG00000237987** | **ENSG00000251380** | **ENSG00000236254** | **ENSG00000257924** | **ENSG00000198854** | **ENSG00000270450** | **ENSG00000211650** |
| **ENSG00000224071** | **ENSG00000269950** | **ENSG00000224523** | **ENSG00000213081** | **ENSG00000241134** | **ENSG00000275011** | **ENSG00000260030** | **ENSG00000170858** |
| **ENSG00000201078** | **ENSG00000255115** | **ENSG00000260177** | **ENSG00000174837** | **ENSG00000258074** | **ENSG00000142273** | **ENSG00000267448** | **ENSG00000263264** |
| **ENSG00000240534** | **ENSG00000257958** | **ENSG00000215984** | **ENSG00000135355** | **ENSG00000211678** | **ENSG00000229361** | **ENSG00000101892** | **ENSG00000153283** |
| **ENSG00000270401** | **ENSG00000197587** | **ENSG00000184293** | **ENSG00000254126** | **ENSG00000211859** | **ENSG00000200105** | **ENSG00000255833** | **ENSG00000255274** |
| **ENSG00000175728** | **ENSG00000162105** | **ENSG00000211867** | **ENSG00000273272** | **ENSG00000266706** | **ENSG00000236858** | **ENSG00000203710** | **ENSG00000168334** |
| **ENSG00000232080** | **ENSG00000214049** | **ENSG00000258181** | **ENSG00000272211** | **ENSG00000267359** | **ENSG00000129455** | **ENSG00000241244** | **ENSG00000242290** |
| **ENSG00000253461** | **ENSG00000119913** | **ENSG00000233501** | **ENSG00000258792** | **ENSG00000202047** | **ENSG00000225289** | **ENSG00000160654** | **ENSG00000280721** |
| **ENSG00000199791** | **ENSG00000143631** | **ENSG00000198883** | **ENSG00000183542** | **ENSG00000211839** | **ENSG00000264340** | **ENSG00000270999** | **ENSG00000211632** |
| **ENSG00000255101** | **ENSG00000166396** | **ENSG00000242222** | **ENSG00000236118** | **ENSG00000225025** | **ENSG00000185962** | **ENSG00000251599** | **ENSG00000267369** |
| **ENSG00000211731** | **ENSG00000069812** | **ENSG00000211810** | **ENSG00000176083** | **ENSG00000229402** | **ENSG00000257757** | **ENSG00000211844** | **ENSG00000211798** |
| **ENSG00000275475** | **ENSG00000157005** | **ENSG00000229424** | **ENSG00000223648** | **ENSG00000238997** | **ENSG00000237330** | **ENSG00000232884** | **ENSG00000253247** |
| **ENSG00000229405** | **ENSG00000137843** | **ENSG00000211809** | **ENSG00000128383** | **ENSG00000211831** | **ENSG00000169900** | **ENSG00000275785** | **ENSG00000110777** |
| **ENSG00000226240** | **ENSG00000230922** | **ENSG00000253451** | **ENSG00000188056** | **ENSG00000255760** | **ENSG00000254239** | **ENSG00000264530** | **ENSG00000211645** |
| **ENSG00000255514** | **ENSG00000248994** | **ENSG00000233887** | **ENSG00000258170** | **ENSG00000181718** | **ENSG00000183249** | **ENSG00000221048** | **ENSG00000185433** |
| **ENSG00000237444** | **ENSG00000279063** | **ENSG00000231280** | **ENSG00000253269** | **ENSG00000211918** | **ENSG00000204572** | **ENSG00000168421** | **ENSG00000260711** |
| **ENSG00000241490** | **ENSG00000154342** | **ENSG00000265488** | **ENSG00000211905** | **ENSG00000211881** | **ENSG00000280558** | **ENSG00000207830** | **ENSG00000256379** |
| **ENSG00000211691** | **ENSG00000261472** | **ENSG00000211792** | **ENSG00000240403** | **ENSG00000134539** | **ENSG00000251338** | **ENSG00000197992** | **ENSG00000278263** |
| **ENSG00000122043** | **ENSG00000221878** | **ENSG00000270325** | **ENSG00000226199** | **ENSG00000279531** | **ENSG00000264785** | **ENSG00000211647** | **ENSG00000177272** |
| **ENSG00000259421** | **ENSG00000064195** | **ENSG00000235196** | **ENSG00000212413** | **ENSG00000254029** | **ENSG00000257920** | **ENSG00000222724** | **ENSG00000249237** |
| **ENSG00000172489** | **ENSG00000088002** | **ENSG00000159618** | **ENSG00000228432** | **ENSG00000252183** | **ENSG00000251256** | **ENSG00000226576** | **ENSG00000253862** |
| **ENSG00000230994** | **ENSG00000227091** | **ENSG00000225825** | **ENSG00000221849** | **ENSG00000224733** | **ENSG00000249731** | **ENSG00000049249** | **ENSG00000238275** |
| **ENSG00000223881** | **ENSG00000167311** | **ENSG00000124256** | **ENSG00000213172** | **ENSG00000232698** | **ENSG00000083307** | **ENSG00000253329** | **ENSG00000277089** |
| **ENSG00000260764** | **ENSG00000166426** | **ENSG00000253240** | **ENSG00000234506** | **ENSG00000261471** | **ENSG00000177108** | **ENSG00000073861** | **ENSG00000264629** |
| **ENSG00000211869** | **ENSG00000237412** | **ENSG00000134545** | **ENSG00000272984** | **ENSG00000228307** | **ENSG00000253768** | **ENSG00000158525** | **ENSG00000251301** |
| **ENSG00000261573** | **ENSG00000121742** | **ENSG00000153064** | **ENSG00000213657** | **ENSG00000275319** | **ENSG00000188334** | **ENSG00000275243** | **ENSG00000211767** |
| **ENSG00000170074** | **ENSG00000274704** | **ENSG00000254098** | **ENSG00000233029** | **ENSG00000245164** | **ENSG00000279387** | **ENSG00000232759** | **ENSG00000211714** |
| **ENSG00000246084** | **ENSG00000167880** | **ENSG00000151962** | **ENSG00000232158** | **ENSG00000253232** | **ENSG00000254024** | **ENSG00000257345** | **ENSG00000228427** |
| **ENSG00000271196** | **ENSG00000279677** | **ENSG00000227508** | **ENSG00000255269** | **ENSG00000207194** | **ENSG00000248317** | **ENSG00000279534** | **ENSG00000256733** |
| **ENSG00000177596** | **ENSG00000104892** | **ENSG00000211787** | **ENSG00000227032** | **ENSG00000260048** | **ENSG00000273225** | **ENSG00000225676** | **ENSG00000255819** |
| **ENSG00000264986** | **ENSG00000165325** | **ENSG00000214866** | **ENSG00000156886** | **ENSG00000211779** | **ENSG00000261436** | **ENSG00000103522** | **ENSG00000264857** |
| **ENSG00000237633** | **ENSG00000236507** | **ENSG00000163114** | **ENSG00000230107** | **ENSG00000248358** | **ENSG00000257084** | **ENSG00000252103** | **ENSG00000244358** |
| **ENSG00000281790** | **ENSG00000275631** | **ENSG00000226938** | **ENSG00000258926** | **ENSG00000268088** | **ENSG00000281273** | **ENSG00000242444** | **ENSG00000213171** |
| **ENSG00000256071** | **ENSG00000166183** | **ENSG00000230539** | **ENSG00000237534** | **ENSG00000226777** | **ENSG00000253783** | **ENSG00000259964** | **ENSG00000223350** |
| **ENSG00000232701** | **ENSG00000260900** | **ENSG00000211593** | **ENSG00000251513** | **ENSG00000211884** | **ENSG00000251616** | **ENSG00000256574** | **ENSG00000211672** |
| **ENSG00000224648** | **ENSG00000221662** | **ENSG00000261453** | **ENSG00000112303** | **ENSG00000211907** | **ENSG00000159455** | **ENSG00000220908** | **ENSG00000242766** |
| **ENSG00000255227** | **ENSG00000188293** | **ENSG00000211840** | **ENSG00000111536** | **ENSG00000211925** | **ENSG00000223259** | **ENSG00000253709** | **ENSG00000253234** |
| **ENSG00000233534** | **ENSG00000223572** | **ENSG00000255428** | **ENSG00000120251** | **ENSG00000253838** | **ENSG00000214514** | **ENSG00000160185** | **ENSG00000139053** |
| **ENSG00000257649** | **ENSG00000164645** | **ENSG00000274508** | **ENSG00000118308** | **ENSG00000187862** | **ENSG00000272703** | **ENSG00000237592** | **ENSG00000279955** |
| **ENSG00000240535** | **ENSG00000232650** | **ENSG00000261127** | **ENSG00000235576** | **ENSG00000278661** | **ENSG00000231132** | **ENSG00000241721** | **ENSG00000224796** |
| **ENSG00000267744** | **ENSG00000075043** | **ENSG00000225477** | **ENSG00000265380** | **ENSG00000211841** | **ENSG00000260131** | **ENSG00000207138** | **ENSG00000244255** |
| **ENSG00000169575** | **ENSG00000085552** | **ENSG00000035720** | **ENSG00000221971** | **ENSG00000201794** | **ENSG00000185742** | **ENSG00000253674** | **ENSG00000004468** |
| **ENSG00000168129** | **ENSG00000134258** | **ENSG00000250706** | **ENSG00000102245** | **ENSG00000254364** | **ENSG00000144452** | **ENSG00000168081** | **ENSG00000253786** |
| **ENSG00000199866** | **ENSG00000189099** | **ENSG00000240056** | **ENSG00000275356** | **ENSG00000128322** | **ENSG00000164451** | **ENSG00000248719** | **ENSG00000233537** |
| **ENSG00000253561** | **ENSG00000171798** | **ENSG00000258560** | **ENSG00000211611** | **ENSG00000164112** | **ENSG00000172782** | **ENSG00000211818** | **ENSG00000102271** |
| **ENSG00000163518** | **ENSG00000264834** | **ENSG00000259374** | **ENSG00000075035** | **ENSG00000272230** | **ENSG00000086696** | **ENSG00000260737** | **ENSG00000253883** |
| **ENSG00000255582** | **ENSG00000226308** | **ENSG00000211765** | **ENSG00000244236** | **ENSG00000243544** | **ENSG00000187180** | **ENSG00000233308** | **ENSG00000259747** |
| **ENSG00000227776** | **ENSG00000264332** | **ENSG00000203852** | **ENSG00000248099** | **ENSG00000204780** | **ENSG00000105141** | **ENSG00000253140** | **ENSG00000187912** |
| **ENSG00000211885** | **ENSG00000261458** | **ENSG00000211751** | **ENSG00000243116** | **ENSG00000136929** | **ENSG00000244222** | **ENSG00000171435** | **ENSG00000169435** |
| **ENSG00000226664** | **ENSG00000272431** | **ENSG00000258637** | **ENSG00000238269** | **ENSG00000224565** | **ENSG00000196734** | **ENSG00000242550** | **ENSG00000231435** |
| **ENSG00000236877** | **ENSG00000230215** | **ENSG00000276014** | **ENSG00000206260** | **ENSG00000165409** | **ENSG00000243550** | **ENSG00000230831** | **ENSG00000242580** |
| **ENSG00000249186** | **ENSG00000158055** | **ENSG00000258386** | **ENSG00000181408** | **ENSG00000181215** | **ENSG00000205331** | **ENSG00000254838** | **ENSG00000230155** |
| **ENSG00000211856** | **ENSG00000230226** | **ENSG00000261239** | **ENSG00000166527** | **ENSG00000233701** | **ENSG00000257998** | **ENSG00000122025** | **ENSG00000230812** |
| **ENSG00000264032** | **ENSG00000182968** | **ENSG00000259162** | **ENSG00000184261** | **ENSG00000239941** | **ENSG00000252332** | **ENSG00000200883** | **ENSG00000275002** |
| **ENSG00000275108** | **ENSG00000103154** | **ENSG00000253481** | **ENSG00000164399** | **ENSG00000272988** | **ENSG00000278529** | **ENSG00000198574** | **ENSG00000234854** |
| **ENSG00000262231** | **ENSG00000274977** | **ENSG00000273612** | **ENSG00000206925** | **ENSG00000232979** | **ENSG00000226480** | **ENSG00000281450** | **ENSG00000166634** |
| **ENSG00000278477** | **ENSG00000183798** | **ENSG00000211946** | **ENSG00000225492** | **ENSG00000238152** | **ENSG00000235242** | **ENSG00000253818** | **ENSG00000253196** |
| **ENSG00000270280** | **ENSG00000279935** | **ENSG00000260943** | **ENSG00000102055** | **ENSG00000211725** | **ENSG00000227528** | **ENSG00000211876** | **ENSG00000197177** |
| **ENSG00000122859** | **ENSG00000234688** | **ENSG00000232591** | **ENSG00000251039** | **ENSG00000234541** | **ENSG00000167741** | **ENSG00000258872** | **ENSG00000115488** |
| **ENSG00000250338** | **ENSG00000252186** | **ENSG00000211782** | **ENSG00000172794** | **ENSG00000205057** | **ENSG00000234620** | **ENSG00000274824** | **ENSG00000263427** |
| **ENSG00000253278** | **ENSG00000161798** | **ENSG00000258385** | **ENSG00000260256** | **ENSG00000235728** | **ENSG00000109182** | **ENSG00000207833** | **ENSG00000266520** |
| **ENSG00000276240** | **ENSG00000176153** | **ENSG00000144460** | **ENSG00000196778** | **ENSG00000226985** | **ENSG00000250517** | **ENSG00000156738** | **ENSG00000261778** |
| **ENSG00000211915** | **ENSG00000279288** | **ENSG00000276210** | **ENSG00000237000** | **ENSG00000260828** | **ENSG00000229009** | **ENSG00000259269** | **ENSG00000135374** |
| **ENSG00000274197** | **ENSG00000271078** | **ENSG00000269919** | **ENSG00000185905** | **ENSG00000234389** | **ENSG00000204949** | **ENSG00000262495** | **ENSG00000233983** |
| **ENSG00000225460** | **ENSG00000189431** | **ENSG00000255151** | **ENSG00000207832** | **ENSG00000234572** | **ENSG00000132677** | **ENSG00000169031** | **ENSG00000254187** |
| **ENSG00000217527** | **ENSG00000255375** | **ENSG00000226025** | **ENSG00000254518** | **ENSG00000281795** | **ENSG00000258625** | **ENSG00000235419** | **ENSG00000218672** |
| **ENSG00000200889** | **ENSG00000266248** | **ENSG00000255998** | **ENSG00000268447** | **ENSG00000266604** | **ENSG00000267924** | **ENSG00000234722** | **ENSG00000222698** |
| **ENSG00000138684** | **ENSG00000137975** | **ENSG00000254030** | **ENSG00000146385** | **ENSG00000211784** | **ENSG00000224308** | **ENSG00000166523** | **ENSG00000179755** |
| **ENSG00000235141** | **ENSG00000196104** | **ENSG00000122224** | **ENSG00000224430** | **ENSG00000244620** | **ENSG00000214856** | **ENSG00000211799** | **ENSG00000268988** |
| **ENSG00000253188** | **ENSG00000177551** | **ENSG00000202309** | **ENSG00000266924** | **ENSG00000227108** | **ENSG00000205847** | **ENSG00000236469** | **ENSG00000237466** |
| **ENSG00000113249** | **ENSG00000235338** | **ENSG00000226217** | **ENSG00000173198** | **ENSG00000237547** | **ENSG00000197595** | **ENSG00000256262** | **ENSG00000112494** |
| **ENSG00000258449** | **ENSG00000205334** | **ENSG00000226702** | **ENSG00000265524** | **ENSG00000228171** | **ENSG00000213022** | **ENSG00000232855** | **ENSG00000249484** |
| **ENSG00000227800** | **ENSG00000232992** | **ENSG00000258150** | **ENSG00000265510** | **ENSG00000258546** | **ENSG00000187223** | **ENSG00000248993** | **ENSG00000215397** |
| **ENSG00000237020** | **ENSG00000018625** | **ENSG00000250485** | **ENSG00000267121** | **ENSG00000255225** | **ENSG00000215353** | **ENSG00000242609** | **ENSG00000254447** |
| **ENSG00000267075** | **ENSG00000104140** | **ENSG00000121380** | **ENSG00000266020** | **ENSG00000172673** | **ENSG00000216708** | **ENSG00000253809** | **ENSG00000261104** |
| **ENSG00000280014** | **ENSG00000210135** | **ENSG00000184033** | **ENSG00000149651** | **ENSG00000231128** | **ENSG00000276703** | **ENSG00000236965** | **ENSG00000229520** |
| **ENSG00000212146** | **ENSG00000250428** | **ENSG00000115607** | **ENSG00000233096** | **ENSG00000235995** | **ENSG00000136697** | **ENSG00000261448** | **ENSG00000267443** |
| **ENSG00000206695** | **ENSG00000254566** | **ENSG00000263961** | **ENSG00000231292** | **ENSG00000251578** | **ENSG00000279458** | **ENSG00000221771** | **ENSG00000269741** |
| **ENSG00000207310** | **ENSG00000149654** | **ENSG00000271569** | **ENSG00000070190** | **ENSG00000239620** | **ENSG00000168703** | **ENSG00000223719** | **ENSG00000205897** |
| **ENSG00000207931** | **ENSG00000257431** | **ENSG00000070915** | **ENSG00000186479** | **ENSG00000271615** | **ENSG00000235942** | **ENSG00000211807** | **ENSG00000142449** |
| **ENSG00000244720** | **ENSG00000259041** | **ENSG00000280350** | **ENSG00000269964** | **ENSG00000276674** | **ENSG00000260377** | **ENSG00000232869** | **ENSG00000239129** |
| **ENSG00000163646** | **ENSG00000281655** | **ENSG00000205045** | **ENSG00000228064** | **ENSG00000248684** | **ENSG00000256906** | **ENSG00000250050** | **ENSG00000265644** |
| **ENSG00000271402** | **ENSG00000267372** | **ENSG00000211713** | **ENSG00000227403** | **ENSG00000223511** | **ENSG00000233017** | **ENSG00000226751** | **ENSG00000251718** |
| **ENSG00000175841** | **ENSG00000170465** | **ENSG00000264572** | **ENSG00000269652** | **ENSG00000271856** | **ENSG00000276850** | **ENSG00000261618** | **ENSG00000258051** |
| **ENSG00000237323** | **ENSG00000271171** | **ENSG00000254246** | **ENSG00000161640** | **ENSG00000224220** | **ENSG00000241776** | **ENSG00000211978** | **ENSG00000254916** |
| **ENSG00000229642** | **ENSG00000150556** | **ENSG00000252142** | **ENSG00000277054** | **ENSG00000233387** | **ENSG00000205867** | **ENSG00000224041** | **ENSG00000223729** |
| **ENSG00000128438** | **ENSG00000178372** | **ENSG00000161270** | **ENSG00000233264** | **ENSG00000270268** | **ENSG00000229699** | **ENSG00000211716** | **ENSG00000259889** |
| **ENSG00000249446** | **ENSG00000244094** | **ENSG00000274752** | **ENSG00000279965** | **ENSG00000258116** | **ENSG00000216014** | **ENSG00000237702** | **ENSG00000231175** |
| **ENSG00000251517** | **ENSG00000171124** | **ENSG00000197721** | **ENSG00000254106** | **ENSG00000226434** | **ENSG00000187664** | **ENSG00000110665** | **ENSG00000224243** |
| **ENSG00000235665** | **ENSG00000069011** | **ENSG00000235366** | **ENSG00000134571** | **ENSG00000278469** | **ENSG00000185966** | **ENSG00000276854** | **ENSG00000251823** |
| **ENSG00000238262** | **ENSG00000171403** | **ENSG00000227009** | **ENSG00000125384** | **ENSG00000249893** | **ENSG00000184032** | **ENSG00000225992** | **ENSG00000203985** |
| **ENSG00000232543** | **ENSG00000239197** | **ENSG00000211819** | **ENSG00000099715** | **ENSG00000243488** | **ENSG00000236466** | **ENSG00000167483** | **ENSG00000094796** |
| **ENSG00000251300** | **ENSG00000228210** | **ENSG00000078589** | **ENSG00000197520** | **ENSG00000270379** | **ENSG00000146926** | **ENSG00000275301** | **ENSG00000184148** |
| **ENSG00000211845** | **ENSG00000244122** | **ENSG00000243664** | **ENSG00000246526** | **ENSG00000211578** | **ENSG00000271969** | **ENSG00000254046** | **ENSG00000257797** |
| **ENSG00000259815** | **ENSG00000259752** | **ENSG00000109943** | **ENSG00000148735** | **ENSG00000259990** | **ENSG00000269124** | **ENSG00000227039** | **ENSG00000241956** |
| **ENSG00000271351** | **ENSG00000254670** | **ENSG00000266305** | **ENSG00000198312** | **ENSG00000256714** | **ENSG00000143520** | **ENSG00000117322** | **ENSG00000271440** |
| **ENSG00000237127** | **ENSG00000166558** | **ENSG00000258835** | **ENSG00000211968** | **ENSG00000163519** | **ENSG00000213030** | **ENSG00000253691** | **ENSG00000281282** |
| **ENSG00000253534** | **ENSG00000261821** | **ENSG00000234282** | **ENSG00000258864** | **ENSG00000143297** | **ENSG00000276335** | **ENSG00000110848** | **ENSG00000177201** |
| **ENSG00000227550** | **ENSG00000179148** | **ENSG00000281347** | **ENSG00000254174** | **ENSG00000254965** | **ENSG00000270400** | **ENSG00000261208** | **ENSG00000274749** |
| **ENSG00000254045** | **ENSG00000279597** | **ENSG00000267664** | **ENSG00000240350** | **ENSG00000236894** | **ENSG00000256346** | **ENSG00000259772** | **ENSG00000179443** |
| **ENSG00000233563** | **ENSG00000233052** | **ENSG00000259954** | **ENSG00000279846** | **ENSG00000254220** | **ENSG00000254603** | **ENSG00000222821** | **ENSG00000265788** |
| **ENSG00000253412** | **ENSG00000188523** | **ENSG00000211797** | **ENSG00000211750** | **ENSG00000251014** | **ENSG00000253715** | **ENSG00000211706** | **ENSG00000255501** |
| **ENSG00000249667** | **ENSG00000137440** | **ENSG00000230489** | **ENSG00000167476** | **ENSG00000222230** | **ENSG00000269989** | **ENSG00000255518** | **ENSG00000235523** |
| **ENSG00000240487** | **ENSG00000234162** | **ENSG00000227061** | **ENSG00000049247** | **ENSG00000235620** | **ENSG00000219159** | **ENSG00000264800** | **ENSG00000278264** |
| **ENSG00000227531** | **ENSG00000100593** | **ENSG00000239215** | **ENSG00000204718** | **ENSG00000268620** | **ENSG00000251985** | **ENSG00000226669** | **ENSG00000265258** |
| **ENSG00000277083** | **ENSG00000182938** | **ENSG00000270472** | **ENSG00000163530** | **ENSG00000262488** | **ENSG00000234398** | **ENSG00000206669** | **ENSG00000139144** |
| **ENSG00000256151** | **ENSG00000229414** | **ENSG00000254802** | **ENSG00000233673** | **ENSG00000211917** | **ENSG00000260741** | **ENSG00000253182** | **ENSG00000258507** |
| **ENSG00000271005** | **ENSG00000188828** | **ENSG00000271507** | **ENSG00000183629** | **ENSG00000237532** | **ENSG00000145920** | **ENSG00000253369** | **ENSG00000252745** |
| **ENSG00000238033** | **ENSG00000253130** | **ENSG00000186265** | **ENSG00000269404** | **ENSG00000235142** | **ENSG00000236595** | **ENSG00000197272** | **ENSG00000264643** |
| **ENSG00000211886** | **ENSG00000170788** | **ENSG00000182156** | **ENSG00000236451** | **ENSG00000281550** | **ENSG00000257664** | **ENSG00000211957** | **ENSG00000259385** |
| **ENSG00000241917** | **ENSG00000076826** | **ENSG00000227920** | **ENSG00000227945** | **ENSG00000224715** | **ENSG00000279578** | **ENSG00000253631** | **ENSG00000279773** |
| **ENSG00000242856** | **ENSG00000198889** | **ENSG00000258867** | **ENSG00000236446** | **ENSG00000156755** | **ENSG00000279754** | **ENSG00000235215** | **ENSG00000248517** |
| **ENSG00000264327** | **ENSG00000108242** | **ENSG00000251916** | **ENSG00000163564** | **ENSG00000253780** | **ENSG00000232841** | **ENSG00000172365** | **ENSG00000244034** |
| **ENSG00000231461** | **ENSG00000223829** | **ENSG00000253448** | **ENSG00000226397** | **ENSG00000176160** | **ENSG00000232596** | **ENSG00000181847** | **ENSG00000232135** |
| **ENSG00000181023** | **ENSG00000155066** | **ENSG00000160683** | **ENSG00000223907** | **ENSG00000256039** | **ENSG00000223740** | **ENSG00000237484** | **ENSG00000225458** |
| **ENSG00000236252** | **ENSG00000205864** | **ENSG00000050730** | **ENSG00000233609** | **ENSG00000211887** | **ENSG00000280093** | **ENSG00000173200** | **ENSG00000083622** |
| **ENSG00000259842** | **ENSG00000167769** | **ENSG00000253535** | **ENSG00000256358** | **ENSG00000255448** | **ENSG00000223026** | **ENSG00000253988** | **ENSG00000273558** |
| **ENSG00000205056** | **ENSG00000215262** | **ENSG00000252619** | **ENSG00000145287** | **ENSG00000211815** | **ENSG00000212657** | **ENSG00000231964** | **ENSG00000257402** |
| **ENSG00000211861** | **ENSG00000238166** | **ENSG00000274466** | **ENSG00000227017** | **ENSG00000254658** | **ENSG00000109193** | **ENSG00000225046** | **ENSG00000276430** |
| **ENSG00000274961** | **ENSG00000173467** | **ENSG00000211786** | **ENSG00000261025** | **ENSG00000232603** | **ENSG00000200506** | **ENSG00000105492** | **ENSG00000188324** |
| **ENSG00000239862** | **ENSG00000163497** | **ENSG00000212447** | **ENSG00000227777** | **ENSG00000230227** | **ENSG00000240393** | **ENSG00000243661** | **ENSG00000171060** |
| **ENSG00000226681** | **ENSG00000166535** | **ENSG00000268845** | **ENSG00000252254** | **ENSG00000111537** | **ENSG00000105143** | **ENSG00000221121** | **ENSG00000260426** |
| **ENSG00000211783** | **ENSG00000221118** | **ENSG00000233235** | **ENSG00000147647** | **ENSG00000181074** | **ENSG00000178395** | **ENSG00000211724** | **ENSG00000260385** |
| **ENSG00000211855** | **ENSG00000188508** | **ENSG00000113302** | **ENSG00000232765** | **ENSG00000211836** | **ENSG00000207581** | **ENSG00000237555** | **ENSG00000226903** |
| **ENSG00000259819** | **ENSG00000140254** | **ENSG00000274603** | **ENSG00000229926** | **ENSG00000232518** | **ENSG00000244057** | **ENSG00000268041** | **ENSG00000175065** |
| **ENSG00000109471** | **ENSG00000214318** | **ENSG00000211689** | **ENSG00000252867** | **ENSG00000223092** | **ENSG00000159337** | **ENSG00000268510** | **ENSG00000240842** |
| **ENSG00000236911** | **ENSG00000188883** | **ENSG00000257925** | **ENSG00000156234** | **ENSG00000253132** | **ENSG00000103546** | **ENSG00000211745** | **ENSG00000186925** |
| **ENSG00000243066** | **ENSG00000260673** | **ENSG00000248978** | **ENSG00000228209** | **ENSG00000270467** | **ENSG00000278172** | **ENSG00000173578** | **ENSG00000149133** |
| **ENSG00000214301** | **ENSG00000278192** | **ENSG00000138472** | **ENSG00000150681** | **ENSG00000276842** | **ENSG00000231870** | **ENSG00000184682** | **ENSG00000253038** |
| **ENSG00000229435** | **ENSG00000117507** | **ENSG00000236008** | **ENSG00000237383** | **ENSG00000225380** | **ENSG00000178597** | **ENSG00000214872** | **ENSG00000252816** |
| **ENSG00000215765** | **ENSG00000079841** | **ENSG00000211791** | **ENSG00000146955** | **ENSG00000255163** | **ENSG00000200168** | **ENSG00000260135** | **ENSG00000260066** |
| **ENSG00000235785** | **ENSG00000186207** | **ENSG00000227722** | **ENSG00000254326** | **ENSG00000211850** | **ENSG00000201920** | **ENSG00000254073** | **ENSG00000172023** |
| **ENSG00000106038** | **ENSG00000249007** | **ENSG00000234425** | **ENSG00000169248** | **ENSG00000276961** | **ENSG00000007216** | **ENSG00000211721** | **ENSG00000253107** |
| **ENSG00000225520** | **ENSG00000199629** | **ENSG00000227303** | **ENSG00000231954** | **ENSG00000243489** | **ENSG00000255960** | **ENSG00000240871** | **ENSG00000241123** |
| **ENSG00000274013** | **ENSG00000006059** | **ENSG00000258360** | **ENSG00000212722** | **ENSG00000139648** | **ENSG00000279211** | **ENSG00000262352** | **ENSG00000198271** |
| **ENSG00000244025** | **ENSG00000202351** | **ENSG00000223201** | **ENSG00000238010** | **ENSG00000186393** | **ENSG00000254190** | **ENSG00000177257** | **ENSG00000272804** |
| **ENSG00000258411** | **ENSG00000135443** | **ENSG00000233036** | **ENSG00000241595** | **ENSG00000173908** | **ENSG00000235431** | **ENSG00000257095** | **ENSG00000204873** |
| **ENSG00000241156** | **ENSG00000204571** | **ENSG00000177511** | **ENSG00000244456** | **ENSG00000214518** | **ENSG00000263761** | **ENSG00000251931** |  |
| **ENSG00000256725** | **ENSG00000276098** | **ENSG00000236151** | **ENSG00000265689** | **ENSG00000244537** | **ENSG00000212721** | **ENSG00000102891** |  |

**TableS2.** The KEGG analysis result of DEGs in high- and low-risk groups in the TCGA cohort.

| **ID** | **Description** | **GeneRatio** | **BgRatio** | **pvalue** | **Count** |
| --- | --- | --- | --- | --- | --- |
| hsa04060 | Cytokine-cytokine receptor interaction | 39/266 | 295/8385 | 1.46E-14 | 39 |
| hsa04061 | Viral protein interaction with cytokine and cytokine receptor | 18/266 | 100/8385 | 1.77E-09 | 18 |
| hsa04640 | Hematopoietic cell lineage | 16/266 | 99/8385 | 7.14E-08 | 16 |
| hsa05340 | Primary immunodeficiency | 9/266 | 38/8385 | 2.07E-06 | 9 |
| hsa04080 | Neuroactive ligand-receptor interaction | 28/266 | 367/8385 | 1.35E-05 | 28 |
| hsa04672 | Intestinal immune network for IgA production | 9/266 | 49/8385 | 1.92E-05 | 9 |
| hsa05150 | Staphylococcus aureus infection | 12/266 | 96/8385 | 4.72E-05 | 12 |
| hsa04660 | T cell receptor signaling pathway | 12/266 | 104/8385 | 1.04E-04 | 12 |
| hsa04630 | JAK-STAT signaling pathway | 15/266 | 166/8385 | 2.44E-04 | 15 |
| hsa04650 | Natural killer cell mediated cytotoxicity | 13/266 | 132/8385 | 2.72E-04 | 13 |
| hsa05332 | Graft-versus-host disease | 7/266 | 42/8385 | 3.11E-04 | 7 |
| hsa04062 | Chemokine signaling pathway | 16/266 | 192/8385 | 3.84E-04 | 16 |
| hsa04612 | Antigen processing and presentation | 9/266 | 78/8385 | 7.63E-04 | 9 |
| hsa05321 | Inflammatory bowel disease | 8/266 | 65/8385 | 9.72E-04 | 8 |
| hsa05330 | Allograft rejection | 6/266 | 38/8385 | 1.13E-03 | 6 |
| hsa04740 | Olfactory transduction | 26/266 | 439/8385 | 1.52E-03 | 26 |
| hsa04940 | Type I diabetes mellitus | 6/266 | 43/8385 | 2.19E-03 | 6 |

**TableS3.** The GO analysis result of DEGs in high- and low-risk groups in the TCGA cohort.

| **ONTOLOGY** | **ID** | **Description** | **GeneRatio** | **BgRatio** | **pvalue** | **Count** |
| --- | --- | --- | --- | --- | --- | --- |
| BP | GO:0002377 | immunoglobulin production | 60/756 | 219/18903 | 1.51E-33 | 60 |
| BP | GO:0002440 | production of molecular mediator of immune response | 70/756 | 333/18903 | 4.56E-31 | 70 |
| BP | GO:0002768 | immune response-regulating cell surface receptor signaling pathway | 57/756 | 341/18903 | 2.56E-20 | 57 |
| BP | GO:0031424 | keratinization | 28/756 | 84/18903 | 8.05E-19 | 28 |
| BP | GO:0030216 | keratinocyte differentiation | 37/756 | 170/18903 | 1.83E-17 | 37 |
| CC | GO:0042101 | T cell receptor complex | 107/787 | 147/19869 | 2.76E-118 | 107 |
| CC | GO:0098802 | plasma membrane signaling receptor complex | 113/787 | 319/19869 | 4.46E-77 | 113 |
| CC | GO:0019814 | immunoglobulin complex | 55/787 | 166/19869 | 7.79E-36 | 55 |
| CC | GO:0009897 | external side of plasma membrane | 74/787 | 462/19869 | 3.86E-25 | 74 |
| CC | GO:0005882 | intermediate filament | 34/787 | 215/19869 | 5.03E-12 | 34 |
| MF | GO:0003823 | antigen binding | 36/647 | 171/18432 | 2.40E-18 | 36 |
| MF | GO:0140375 | immune receptor activity | 29/647 | 148/18432 | 3.60E-14 | 29 |
| MF | GO:0042287 | MHC protein binding | 14/647 | 42/18432 | 7.99E-11 | 14 |
| MF | GO:0034987 | immunoglobulin receptor binding | 16/647 | 79/18432 | 1.22E-08 | 16 |
| MF | GO:0016493 | C-C chemokine receptor activity | 9/647 | 23/18432 | 4.02E-08 | 9 |

**TableS4.** The clinical characteristics of the patients in the TCGA cohort.

| **samples** | **Age** | **Gender** | **Stage** | **Breslow depth** | **BRAF mutant** | **NRAS mutant** | **Tumor location** | **Sample type** | **Ulceration** | **Radiation therapy** | **Immunotherapy** | **Chemotherapy** |
| --- | --- | --- | --- | --- | --- | --- | --- | --- | --- | --- | --- | --- |
| **TCGA-GN-A263-01** | 24 | Male | IV | NA | Mutant | WT | Primary Tumor | Primary Tumor | YES | NA | NO | NO |
| **TCGA-EB-A431-01** | 34 | Male | II | 15 | Mutant | WT | Primary Tumor | Primary Tumor | YES | NO | NO | NO |
| **TCGA-BF-A3DJ-01** | 36 | Female | III | 11 | Mutant | WT | Primary Tumor | Primary Tumor | YES | NA | NO | NO |
| **TCGA-EB-A3HV-01** | 37 | Male | II | 13 | Mutant | WT | Primary Tumor | Primary Tumor | YES | NA | NO | NO |
| **TCGA-GF-A769-01** | 39 | Male | II | 74 | Mutant | WT | Primary Tumor | Primary Tumor | YES | NA | NO | NO |
| **TCGA-EB-A4OZ-01** | 41 | Female | III | 18 | WT | WT | Primary Tumor | Primary Tumor | NO | NO | NO | NO |
| **TCGA-EB-A4IQ-01** | 42 | Female | III | 15 | WT | WT | Primary Tumor | Primary Tumor | YES | NO | NO | NO |
| **TCGA-D3-A5GT-01** | 43 | Male | III | 1.92 | Mutant | WT | Primary Tumor | Primary Tumor | YES | NO | YES | NO |
| **TCGA-FS-A1ZN-01** | 43 | Male | III | 8 | Mutant | WT | Primary Tumor | Primary Tumor | YES | NA | NO | NO |
| **TCGA-BF-A1PU-01** | 46 | Female | II | 13 | Mutant | WT | Primary Tumor | Primary Tumor | YES | NA | NO | NO |
| **TCGA-EB-A1NK-01** | 48 | Male | II | 8 | WT | Mutant | Primary Tumor | Primary Tumor | YES | NO | NO | NO |
| **TCGA-EB-A5UM-01** | 48 | Female | II | 5 | WT | WT | Primary Tumor | Primary Tumor | YES | NO | NO | NO |
| **TCGA-GF-A2C7-01** | 48 | Male | II | NA | Mutant | WT | Primary Tumor | Primary Tumor | YES | NO | NO | NO |
| **TCGA-FR-A2OS-01** | 49 | Female | II | NA | WT | WT | Primary Tumor | Primary Tumor | YES | NO | NO | YES |
| **TCGA-EB-A42Z-01** | 49 | Male | III | 10 | Mutant | WT | Primary Tumor | Primary Tumor | YES | NO | NO | NO |
| **TCGA-D9-A4Z2-01** | 50 | Male | III | 25 | Mutant | WT | Primary Tumor | Primary Tumor | YES | NO | NO | NO |
| **TCGA-ER-A19T-01** | 51 | Male | IV | 15 | WT | WT | Primary Tumor | Primary Tumor | NO | YES | NO | NO |
| **TCGA-XV-A9W5-01** | 51 | Male | I/II | 2 | Mutant | WT | Primary Tumor | Primary Tumor | NA | NO | YES | NO |
| **TCGA-ER-A2NF-01** | 53 | Male | III | 4 | WT | WT | Primary Tumor | Primary Tumor | YES | NO | YES | YES |
| **TCGA-EB-A3XD-01** | 53 | Female | II | NA | WT | WT | Primary Tumor | Primary Tumor | NA | NO | NO | NO |
| **TCGA-EB-A51B-01** | 53 | Male | II | 10 | WT | WT | Primary Tumor | Primary Tumor | YES | NO | NO | NO |
| **TCGA-FR-A728-01** | 54 | Female | III | 12 | Mutant | WT | Primary Tumor | Primary Tumor | YES | NO | NO | YES |
| **TCGA-D9-A3Z4-01** | 54 | Male | III | 12 | WT | Mutant | Primary Tumor | Primary Tumor | YES | NO | NO | NO |
| **TCGA-BF-AAP6-01** | 55 | Male | III | 20 | Mutant | WT | Primary Tumor | Primary Tumor | YES | NO | NO | NO |
| **TCGA-EB-A24C-01** | 56 | Male | NA | 10 | Mutant | WT | Primary Tumor | Primary Tumor | YES | NO | YES | YES |
| **TCGA-EB-A3Y6-01** | 56 | Female | II | 4.5 | Mutant | WT | Primary Tumor | Primary Tumor | YES | NO | NO | YES |
| **TCGA-EB-A57M-01** | 56 | Male | III | 8 | WT | WT | Primary Tumor | Primary Tumor | YES | NO | YES | YES |
| **TCGA-BF-A1PX-01** | 56 | Male | III | 12 | WT | WT | Primary Tumor | Primary Tumor | YES | NA | NO | NO |
| **TCGA-EB-A4XL-01** | 56 | Female | II | 7 | WT | WT | Primary Tumor | Primary Tumor | YES | NO | NO | NO |
| **TCGA-EB-A5VU-01** | 56 | Male | III | 15 | WT | Mutant | Primary Tumor | Primary Tumor | YES | NO | NO | NO |
| **TCGA-XV-AAZV-01** | 56 | Female | II | 10 | Mutant | WT | Primary Tumor | Primary Tumor | NA | NO | NO | NO |
| **TCGA-EB-A3XF-01** | 57 | Male | II | 10 | Mutant | WT | Primary Tumor | Primary Tumor | YES | NO | NO | NO |
| **TCGA-ER-A2NB-01** | 57 | Male | III | 4.39 | WT | Mutant | Primary Tumor | Primary Tumor | YES | NO | NO | NO |
| **TCGA-EB-A6R0-01** | 58 | Female | II | 10 | Mutant | WT | Primary Tumor | Primary Tumor | YES | NO | NO | YES |
| **TCGA-BF-AAP8-01** | 58 | Male | II | 6 | WT | WT | Primary Tumor | Primary Tumor | YES | NO | NO | NO |
| **TCGA-EB-A44P-01** | 58 | Female | II | NA | Mutant | WT | Primary Tumor | Primary Tumor | NO | NO | NO | NO |
| **TCGA-EB-A82B-01** | 58 | Female | III | 20 | WT | WT | Primary Tumor | Primary Tumor | YES | NO | NO | NO |
| **TCGA-EB-A44N-01** | 59 | Male | II | 5 | WT | WT | Primary Tumor | Primary Tumor | YES | NO | NO | NO |
| **TCGA-BF-AAP4-01** | 61 | Male | II | 15 | WT | WT | Primary Tumor | Primary Tumor | YES | NO | NO | NO |
| **TCGA-GN-A4U5-01** | 61 | Female | I | 1.15 | Mutant | WT | Primary Tumor | Primary Tumor | NO | NO | YES | NO |
| **TCGA-IH-A3EA-01** | 61 | Male | II | NA | Mutant | WT | Primary Tumor | Primary Tumor | YES | NA | NO | NO |
| **TCGA-EB-A553-01** | 62 | Male | II | 10 | Mutant | WT | Primary Tumor | Primary Tumor | YES | NO | NO | YES |
| **TCGA-BF-AAP2-01** | 62 | Male | II | 3 | WT | Mutant | Primary Tumor | Primary Tumor | YES | NO | NO | NO |
| **TCGA-XV-AAZW-01** | 62 | Female | II | 13 | Mutant | WT | Primary Tumor | Primary Tumor | NA | NO | NO | NO |
| **TCGA-BF-A3DM-01** | 63 | Male | II | 1.5 | Mutant | WT | Primary Tumor | Primary Tumor | YES | NO | NO | NO |
| **TCGA-BF-A5EQ-01** | 63 | Male | II | 5 | Mutant | WT | Primary Tumor | Primary Tumor | NA | NO | NO | NO |
| **TCGA-BF-A5ER-01** | 63 | Male | II | 14 | Mutant | WT | Primary Tumor | Primary Tumor | YES | NO | NO | NO |
| **TCGA-EB-A299-01** | 63 | Male | II | 2 | Mutant | WT | Primary Tumor | Primary Tumor | YES | NO | NO | NO |
| **TCGA-EB-A3XB-01** | 63 | Male | II | NA | Mutant | WT | Primary Tumor | Primary Tumor | NA | NO | NO | NO |
| **TCGA-ER-A196-01** | 64 | Female | II | 22 | WT | WT | Primary Tumor | Primary Tumor | YES | NO | NO | NO |
| **TCGA-EB-A4OY-01** | 65 | Female | III | 10 | WT | WT | Primary Tumor | Primary Tumor | NA | NO | NO | YES |
| **TCGA-EB-A5FP-01** | 65 | Female | IV | 8 | Mutant | WT | Primary Tumor | Primary Tumor | YES | NO | NO | YES |
| **TCGA-BF-A5EO-01** | 65 | Male | II | 8 | WT | Mutant | Primary Tumor | Primary Tumor | YES | NO | NO | NO |
| **TCGA-EB-A85J-01** | 66 | Female | II | 5.5 | WT | Mutant | Primary Tumor | Primary Tumor | NO | NO | NO | YES |
| **TCGA-EB-A85I-01** | 66 | Male | II | 10 | Mutant | WT | Primary Tumor | Primary Tumor | YES | NO | NO | NO |
| **TCGA-EB-A97M-01** | 66 | Male | II | 15 | WT | WT | Primary Tumor | Primary Tumor | YES | NO | NO | NO |
| **TCGA-YG-AA3N-01** | 67 | Male | II | 17 | Mutant | WT | Primary Tumor | Primary Tumor | NA | NO | NO | NO |
| **TCGA-GN-A8LN-01** | 68 | Male | II | 4.85 | WT | Mutant | Primary Tumor | Primary Tumor | YES | NO | YES | YES |
| **TCGA-D9-A4Z5-01** | 68 | Male | II | NA | Mutant | WT | NA | Primary Tumor | NO | NO | NO | NO |
| **TCGA-EB-A44O-01** | 69 | Male | II | 5 | WT | Mutant | Primary Tumor | Primary Tumor | NO | NO | NO | NO |
| **TCGA-FR-A3R1-01** | 69 | Male | II | 6.3 | WT | WT | Primary Tumor | Primary Tumor | YES | NO | NO | NO |
| **TCGA-EB-A82C-01** | 70 | Female | II | 10 | WT | WT | Primary Tumor | Primary Tumor | YES | NA | NO | NO |
| **TCGA-GN-A269-01** | 70 | Male | III | 5 | WT | WT | Primary Tumor | Primary Tumor | YES | NA | NO | NO |
| **TCGA-EB-A6QY-01** | 71 | Male | II | 10 | Mutant | WT | Primary Tumor | Primary Tumor | YES | NO | YES | YES |
| **TCGA-BF-A1PZ-01** | 71 | Female | II | 8 | WT | Mutant | Primary Tumor | Primary Tumor | NO | NO | NO | NO |
| **TCGA-FW-A5DX-01** | 71 | Male | III | 7 | Mutant | WT | Primary Tumor | Primary Tumor | NO | NO | NO | NO |
| **TCGA-EB-A24D-01** | 72 | Male | III | 36 | Mutant | WT | Primary Tumor | Primary Tumor | NO | NO | YES | YES |
| **TCGA-BF-AAOU-01** | 73 | Female | II | 12 | WT | WT | Primary Tumor | Primary Tumor | YES | NO | NO | NO |
| **TCGA-D9-A4Z3-01** | 73 | Female | III | 75 | WT | Mutant | Primary Tumor | Primary Tumor | YES | NO | NO | NO |
| **TCGA-DA-A960-01** | 73 | Male | II | 2.32 | WT | Mutant | Regional Cutaneous or Subcutaneous Tissue | Primary Tumor | YES | NO | NO | NO |
| **TCGA-EB-A42Y-01** | 73 | Female | II | 5 | WT | WT | Primary Tumor | Primary Tumor | YES | NO | NO | NO |
| **TCGA-EB-A5SE-01** | 73 | Male | II | 4 | Mutant | WT | Primary Tumor | Primary Tumor | YES | NO | NO | NO |
| **TCGA-EB-A3XC-01** | 74 | Male | II | NA | Mutant | WT | Primary Tumor | Primary Tumor | YES | NO | NO | YES |
| **TCGA-BF-A1PV-01** | 74 | Female | II | 9 | WT | Mutant | Primary Tumor | Primary Tumor | YES | NA | NO | NO |
| **TCGA-BF-A5EP-01** | 75 | Female | III | 70 | WT | WT | Primary Tumor | Primary Tumor | YES | NO | NO | NO |
| **TCGA-EB-A550-01** | 75 | Female | II | 15 | Mutant | WT | Primary Tumor | Primary Tumor | YES | NO | NO | NO |
| **TCGA-EB-A6QZ-01** | 76 | Female | II | 3 | Mutant | WT | Primary Tumor | Primary Tumor | NO | YES | NO | YES |
| **TCGA-BF-A5ES-01** | 76 | Female | II | 8 | WT | WT | Primary Tumor | Primary Tumor | YES | NO | NO | NO |
| **TCGA-BF-AAP7-01** | 76 | Female | II | 5 | Mutant | WT | Primary Tumor | Primary Tumor | YES | NO | NO | NO |
| **TCGA-EB-A41B-01** | 76 | Female | II | 28 | WT | WT | Primary Tumor | Primary Tumor | YES | NO | NO | NO |
| **TCGA-ER-A42H-01** | 76 | Male | NA | NA | WT | WT | Primary Tumor | Primary Tumor | NA | NA | NO | NO |
| **TCGA-XV-AAZY-01** | 76 | Female | III | 16 | WT | Mutant | Primary Tumor | Primary Tumor | NA | NO | NO | NO |
| **TCGA-BF-A9VF-01** | 77 | Male | II | 12 | Mutant | WT | Primary Tumor | Primary Tumor | YES | NO | NO | NO |
| **TCGA-EB-A3XE-01** | 77 | Female | II | 3 | Mutant | WT | Primary Tumor | Primary Tumor | NO | NO | NO | NO |
| **TCGA-EB-A4IS-01** | 77 | Male | II | 2.5 | Mutant | WT | Primary Tumor | Primary Tumor | YES | NO | NO | NO |
| **TCGA-ER-A194-01** | 77 | Male | NA | 4.5 | WT | WT | Primary Tumor | Primary Tumor | YES | NA | NO | NO |
| **TCGA-GN-A26C-01** | 77 | Male | III | 14 | WT | WT | Primary Tumor | Primary Tumor | YES | NA | NO | NO |
| **TCGA-EB-A551-01** | 78 | Female | III | 10 | WT | Mutant | Primary Tumor | Primary Tumor | YES | NO | NO | NO |
| **TCGA-EB-A5SF-01** | 78 | Female | II | 11 | WT | WT | Primary Tumor | Primary Tumor | YES | NO | NO | NO |
| **TCGA-ER-A19K-01** | 79 | Female | II | 6.8 | Mutant | WT | Primary Tumor | Primary Tumor | YES | NA | NO | YES |
| **TCGA-BF-A1Q0-01** | 80 | Male | II | 5 | WT | WT | Primary Tumor | Primary Tumor | YES | NO | NO | NO |
| **TCGA-BF-A3DN-01** | 81 | Female | III | 3 | Mutant | WT | Primary Tumor | Primary Tumor | YES | NO | NO | NO |
| **TCGA-XV-A9W2-01** | 81 | Male | I | 1 | WT | WT | Primary Tumor | Primary Tumor | NA | NO | NO | NO |
| **TCGA-EB-A4P0-01** | 82 | Male | II | 8 | Mutant | WT | Primary Tumor | Primary Tumor | YES | NO | NO | NO |
| **TCGA-BF-AAOX-01** | 83 | Male | II | 11 | WT | Mutant | Primary Tumor | Primary Tumor | YES | NO | NO | NO |
| **TCGA-BF-A3DL-01** | 84 | Female | III | 3 | Mutant | WT | Primary Tumor | Primary Tumor | YES | NO | NO | NO |
| **TCGA-WE-A8K4-01** | 85 | Male | II | 12 | WT | WT | Primary Tumor | Primary Tumor | NA | NO | NO | NO |
| **TCGA-BF-AAP1-01** | 86 | Male | II | 15 | Mutant | WT | Primary Tumor | Primary Tumor | YES | NO | NO | NO |
| **TCGA-EB-A3Y7-01** | 86 | Female | III | 4 | WT | WT | Primary Tumor | Primary Tumor | NO | NO | NO | NO |
| **TCGA-D3-A1QA-07** | 55 | Male | I | 1.12 | NA | NA | Regional Lymph Node | Metastatic | NO | NO | NO | NO |
| **TCGA-D3-A5GN-06** | 15 | Female | I | 0.6 | Mutant | WT | Regional Lymph Node | Metastatic | NA | NA | NO | NO |
| **TCGA-D3-A8GJ-06** | 18 | Male | II | 2.4 | Mutant | WT | Distant Metastasis | Metastatic | NA | NO | NO | NO |
| **TCGA-EE-A2MK-06** | 18 | Female | III | 5.2 | Mutant | WT | Regional Lymph Node | Metastatic | NO | NO | NO | NO |
| **TCGA-D3-A51J-06** | 19 | Male | III | NA | Mutant | WT | Regional Cutaneous or Subcutaneous Tissue | Metastatic | NA | NO | NO | NO |
| **TCGA-FS-A1Z7-06** | 19 | Male | III | 17 | Mutant | WT | Regional Lymph Node | Metastatic | YES | NA | NO | NO |
| **TCGA-EE-A29C-06** | 20 | Male | I | 1.7 | Mutant | WT | Regional Lymph Node | Metastatic | NO | NO | YES | YES |
| **TCGA-EE-A2MG-06** | 23 | Male | I | 0.7 | WT | WT | Regional Lymph Node | Metastatic | NA | NA | NO | YES |
| **TCGA-D3-A5GR-06** | 23 | Female | III | 0.98 | Mutant | WT | Regional Lymph Node | Metastatic | NA | NO | NO | NO |
| **TCGA-D3-A2JK-06** | 24 | Male | III | 4.3 | Mutant | WT | Regional Lymph Node | Metastatic | YES | NA | NO | NO |
| **TCGA-LH-A9QB-06** | 24 | Female | NA | NA | Mutant | WT | Distant Metastasis | Metastatic | NA | NO | NO | NO |
| **TCGA-D3-A8GV-06** | 25 | Male | I/II | NA | Mutant | WT | Distant Metastasis | Metastatic | NA | NA | NO | NO |
| **TCGA-EE-A3AG-06** | 25 | Male | III | NA | Mutant | WT | Regional Cutaneous or Subcutaneous Tissue | Metastatic | NA | NO | NO | NO |
| **TCGA-ER-A3ES-06** | 25 | Male | NA | NA | WT | WT | Distant Metastasis | Metastatic | NA | NA | NO | NO |
| **TCGA-WE-A8ZT-06** | 25 | Female | IV | 4 | Mutant | WT | Regional Lymph Node | Metastatic | YES | NO | NO | NO |
| **TCGA-W3-AA21-06** | 26 | Male | I | 1.1 | Mutant | WT | Regional Lymph Node | Metastatic | NO | NA | YES | YES |
| **TCGA-D3-A8GE-06** | 26 | Male | IV | NA | WT | WT | Regional Cutaneous or Subcutaneous Tissue | Metastatic | NA | NO | NO | NO |
| **TCGA-D3-A8GN-06** | 27 | Female | I/II | NA | Mutant | WT | Distant Metastasis | Metastatic | NA | NO | YES | NO |
| **TCGA-DA-A1I5-06** | 27 | Female | IV | 0.6 | Mutant | WT | Distant Metastasis | Metastatic | NO | NO | NO | NO |
| **TCGA-EE-A2GS-06** | 28 | Female | I | 1.8 | Mutant | WT | Regional Cutaneous or Subcutaneous Tissue | Metastatic | NO | NO | YES | NO |
| **TCGA-FR-A44A-06** | 29 | Female | II | 2.51 | Mutant | WT | Regional Lymph Node | Metastatic | NO | YES | YES | NO |
| **TCGA-EE-A3AH-06** | 30 | Male | II | 3.7 | Mutant | WT | Regional Lymph Node | Metastatic | NO | NA | NO | YES |
| **TCGA-D3-A2JG-06** | 30 | Female | III | 2.5 | Mutant | WT | Regional Lymph Node | Metastatic | NO | NA | NO | NO |
| **TCGA-EE-A3AB-06** | 30 | Male | III | NA | Mutant | WT | Regional Lymph Node | Metastatic | NA | NA | NO | NO |
| **TCGA-ER-A3PL-06** | 30 | Male | IV | 2.2 | Mutant | WT | Regional Cutaneous or Subcutaneous Tissue | Metastatic | YES | NO | NO | NO |
| **TCGA-GN-A4U3-06** | 30 | Male | III | 3 | WT | Mutant | Distant Metastasis | Metastatic | NO | YES | YES | NO |
| **TCGA-FS-A1ZQ-06** | 31 | Male | I/II | NA | WT | Mutant | Distant Metastasis | Metastatic | NA | NA | NO | NO |
| **TCGA-DA-A1IA-06** | 32 | Female | III | 1.3 | WT | WT | Distant Metastasis | Metastatic | NO | NO | NO | NO |
| **TCGA-FS-A1Z0-06** | 32 | Female | I | 0.95 | WT | Mutant | Distant Metastasis | Metastatic | NO | NA | NO | NO |
| **TCGA-D3-A1Q8-06** | 33 | Male | IV | NA | WT | WT | Regional Lymph Node | Metastatic | NA | NA | NO | NO |
| **TCGA-EE-A29M-06** | 33 | Female | I | 1.02 | Mutant | WT | Regional Lymph Node | Metastatic | NO | NO | NO | NO |
| **TCGA-EE-A2GH-06** | 34 | Male | I | 0.8 | Mutant | WT | Regional Lymph Node | Metastatic | NA | NO | NO | NO |
| **TCGA-EE-A2MP-06** | 34 | Female | I | 1.05 | Mutant | WT | Regional Lymph Node | Metastatic | NA | NA | NO | NO |
| **TCGA-FR-A69P-06** | 34 | Female | III | NA | WT | Mutant | Regional Lymph Node | Metastatic | NA | YES | YES | NO |
| **TCGA-EE-A2ML-06** | 35 | Male | II | 3 | WT | Mutant | Regional Lymph Node | Metastatic | NO | NO | YES | YES |
| **TCGA-ER-A19L-06** | 35 | Male | NA | NA | WT | WT | Regional Lymph Node | Metastatic | NA | NA | NO | NO |
| **TCGA-ER-A19E-06** | 36 | Female | I | 1.1 | Mutant | WT | Regional Lymph Node | Metastatic | NO | NA | NO | YES |
| **TCGA-D3-A5GU-06** | 36 | Male | I | 0.48 | Mutant | WT | Regional Lymph Node | Metastatic | NO | NA | NO | NO |
| **TCGA-ER-A19M-06** | 36 | Male | I | 1.9 | Mutant | WT | Regional Lymph Node | Metastatic | NO | NA | YES | NO |
| **TCGA-FS-A1ZR-06** | 36 | Male | II | 2 | Mutant | WT | Regional Cutaneous or Subcutaneous Tissue | Metastatic | NA | NA | NO | NO |
| **TCGA-ER-A19Q-06** | 37 | Female | NA | 1.2 | Mutant | WT | Regional Lymph Node | Metastatic | NO | NA | YES | YES |
| **TCGA-D3-A2JP-06** | 37 | Male | III | NA | Mutant | WT | Regional Lymph Node | Metastatic | NA | NO | YES | NO |
| **TCGA-DA-A1HW-06** | 37 | Female | III | 0.7 | Mutant | WT | Regional Lymph Node | Metastatic | NO | NO | NO | NO |
| **TCGA-WE-AA9Y-06** | 37 | Male | III | 1.4 | WT | WT | Regional Lymph Node | Metastatic | NO | NO | NO | NO |
| **TCGA-GN-A267-06** | 38 | Male | III | 5.2 | WT | Mutant | Regional Lymph Node | Metastatic | NO | NA | NO | YES |
| **TCGA-D3-A3MV-06** | 38 | Female | III | 1.2 | WT | Mutant | Regional Cutaneous or Subcutaneous Tissue | Metastatic | YES | NO | YES | NO |
| **TCGA-FR-A729-06** | 38 | Female | I | 0.25 | Mutant | WT | Regional Lymph Node | Metastatic | NO | NO | YES | NO |
| **TCGA-D3-A51E-06** | 39 | Female | I/II | 1.2 | WT | Mutant | Regional Cutaneous or Subcutaneous Tissue | Metastatic | NA | NO | NO | YES |
| **TCGA-D3-A3CB-06** | 39 | Male | I/II | 1.75 | Mutant | WT | Regional Lymph Node | Metastatic | NA | NO | NO | NO |
| **TCGA-D9-A3Z3-06** | 39 | Female | III | 3.9 | Mutant | WT | Regional Lymph Node | Metastatic | NO | NO | NO | NO |
| **TCGA-DA-A3F8-06** | 39 | Male | III | 1.9 | Mutant | WT | Regional Lymph Node | Metastatic | NO | NO | NO | NO |
| **TCGA-EE-A2GI-06** | 39 | Male | I | 0.65 | WT | WT | Regional Lymph Node | Metastatic | NO | NO | NO | NO |
| **TCGA-EE-A2MF-06** | 39 | Female | I | 1.5 | WT | Mutant | Regional Lymph Node | Metastatic | NA | NA | NO | NO |
| **TCGA-ER-A2NE-06** | 39 | Male | 0 | NA | WT | Mutant | Distant Metastasis | Metastatic | NA | NA | NO | NO |
| **TCGA-FS-A1YX-06** | 39 | Female | I | 1.1 | Mutant | WT | Regional Cutaneous or Subcutaneous Tissue | Metastatic | NO | NA | NO | NO |
| **TCGA-FS-A4FD-06** | 39 | Male | III | 2 | WT | Mutant | Regional Lymph Node | Metastatic | NA | NO | NO | NO |
| **TCGA-ER-A19H-06** | 40 | Male | NA | 0.4 | Mutant | WT | Regional Cutaneous or Subcutaneous Tissue | Metastatic | NA | NA | NO | YES |
| **TCGA-FS-A1ZE-06** | 40 | Male | II | 5 | WT | Mutant | Distant Metastasis | Metastatic | YES | NO | NO | YES |
| **TCGA-BF-AAP0-06** | 40 | Female | IV | NA | Mutant | WT | Regional Lymph Node | Metastatic | NA | NO | NO | NO |
| **TCGA-D9-A148-06** | 40 | Male | NA | NA | Mutant | WT | Regional Lymph Node | Metastatic | NO | YES | YES | NO |
| **TCGA-EE-A2GL-06** | 40 | Female | II | 3.1 | WT | Mutant | Regional Lymph Node | Metastatic | NO | NO | NO | NO |
| **TCGA-ER-A42K-06** | 40 | Female | III | 5.5 | Mutant | WT | Regional Lymph Node | Metastatic | YES | NO | NO | NO |
| **TCGA-FR-A8YE-06** | 41 | Male | I | 0.79 | Mutant | WT | Regional Lymph Node | Metastatic | NO | NO | NO | NO |
| **TCGA-D3-A1Q7-06** | 42 | Female | I | 0.68 | WT | WT | Regional Lymph Node | Metastatic | NO | YES | NO | NO |
| **TCGA-D3-A3MR-06** | 42 | Male | III | NA | Mutant | WT | Regional Cutaneous or Subcutaneous Tissue | Metastatic | NA | NO | NO | NO |
| **TCGA-DA-A1HY-06** | 42 | Male | III | 1.13 | Mutant | WT | Regional Lymph Node | Metastatic | YES | NO | NO | NO |
| **TCGA-EE-A29W-06** | 42 | Male | 0 | 0 | Mutant | WT | Regional Lymph Node | Metastatic | NA | YES | NO | NO |
| **TCGA-EE-A3J3-06** | 42 | Male | I | 0.98 | Mutant | WT | Regional Cutaneous or Subcutaneous Tissue | Metastatic | NA | NA | NO | NO |
| **TCGA-ER-A19B-06** | 42 | Male | NA | NA | Mutant | WT | Regional Cutaneous or Subcutaneous Tissue | Metastatic | NA | NA | NO | NO |
| **TCGA-Z2-A8RT-06** | 42 | Female | II | 4 | WT | WT | Regional Cutaneous or Subcutaneous Tissue | Metastatic | YES | NO | NO | NO |
| **TCGA-EE-A2A5-06** | 43 | Male | I | 1.1 | WT | Mutant | Regional Cutaneous or Subcutaneous Tissue | Metastatic | NO | NA | NO | YES |
| **TCGA-ER-A2NG-06** | 43 | Female | III | 3 | Mutant | WT | Regional Lymph Node | Metastatic | YES | NO | YES | YES |
| **TCGA-D3-A2JL-06** | 43 | Female | I/II | NA | WT | WT | Regional Lymph Node | Metastatic | NA | NO | NO | NO |
| **TCGA-D3-A8GL-06** | 43 | Male | III | 1.32 | WT | Mutant | Regional Cutaneous or Subcutaneous Tissue | Metastatic | NO | NA | NO | NO |
| **TCGA-EE-A2A6-06** | 43 | Male | I | 0.55 | Mutant | WT | Regional Lymph Node | Metastatic | NO | NO | NO | NO |
| **TCGA-EE-A2MI-06** | 43 | Male | II | 0.4 | WT | WT | Regional Lymph Node | Metastatic | NA | NA | NO | NO |
| **TCGA-EE-A3J7-06** | 43 | Male | I | 1.12 | WT | Mutant | Regional Cutaneous or Subcutaneous Tissue | Metastatic | YES | NA | NO | NO |
| **TCGA-YD-A89C-06** | 43 | Female | I | 1 | WT | Mutant | Distant Metastasis | Metastatic | NO | NA | NO | NO |
| **TCGA-EE-A2GE-06** | 44 | Male | I | 0.9 | WT | WT | Regional Lymph Node | Metastatic | NO | YES | NO | NO |
| **TCGA-EE-A3JA-06** | 44 | Male | I | 1.5 | WT | Mutant | Regional Lymph Node | Metastatic | NO | NA | NO | NO |
| **TCGA-FR-A3YN-06** | 44 | Male | I | 1.25 | WT | Mutant | Regional Lymph Node | Metastatic | NO | NO | YES | NO |
| **TCGA-ER-A198-06** | 45 | Male | NA | 10 | Mutant | WT | Regional Cutaneous or Subcutaneous Tissue | Metastatic | YES | NA | NO | YES |
| **TCGA-D3-A8GK-06** | 45 | Male | II | 2.6 | WT | Mutant | Distant Metastasis | Metastatic | NO | YES | NO | NO |
| **TCGA-DA-A1I2-06** | 45 | Male | III | NA | Mutant | WT | Regional Lymph Node | Metastatic | YES | NO | NO | NO |
| **TCGA-DA-A3F5-06** | 45 | Male | I | 0.4 | Mutant | WT | Regional Lymph Node | Metastatic | NA | NO | YES | NO |
| **TCGA-EE-A2MT-06** | 45 | Male | I | 1.5 | WT | WT | Regional Lymph Node | Metastatic | NO | YES | NO | NO |
| **TCGA-FS-A1ZA-06** | 45 | Female | III | 4.5 | WT | Mutant | Distant Metastasis | Metastatic | YES | NA | NO | NO |
| **TCGA-GN-A266-06** | 45 | Male | NA | NA | WT | WT | Regional Cutaneous or Subcutaneous Tissue | Metastatic | NA | NA | NO | NO |
| **TCGA-WE-A8ZX-06** | 45 | Male | III | NA | Mutant | WT | Regional Lymph Node | Metastatic | NA | YES | NO | NO |
| **TCGA-HR-A2OH-06** | 46 | Female | III | 3.4 | Mutant | WT | Distant Metastasis | Metastatic | YES | YES | YES | YES |
| **TCGA-D3-A2JN-06** | 46 | Female | III | NA | Mutant | WT | Regional Lymph Node | Metastatic | NA | NO | YES | NO |
| **TCGA-EE-A2A1-06** | 46 | Male | I | 1.3 | WT | WT | Regional Lymph Node | Metastatic | NO | NO | NO | NO |
| **TCGA-EE-A2GK-06** | 46 | Female | I | 0.8 | WT | WT | Regional Lymph Node | Metastatic | NA | NA | NO | NO |
| **TCGA-ER-A195-06** | 46 | Male | NA | NA | WT | Mutant | Regional Lymph Node | Metastatic | NA | NA | YES | NO |
| **TCGA-ER-A19D-06** | 46 | Female | I | 1.75 | WT | Mutant | Regional Cutaneous or Subcutaneous Tissue | Metastatic | NO | NA | NO | NO |
| **TCGA-FS-A4F2-06** | 46 | Female | II | 4.7 | WT | WT | Regional Cutaneous or Subcutaneous Tissue | Metastatic | YES | NA | NO | NO |
| **TCGA-FS-A4FB-06** | 46 | Female | III | 2 | Mutant | WT | Regional Lymph Node | Metastatic | NA | NA | NO | NO |
| **TCGA-D3-A3MO-06** | 47 | Male | III | NA | Mutant | WT | Regional Cutaneous or Subcutaneous Tissue | Metastatic | NA | NA | NO | NO |
| **TCGA-EE-A3AA-06** | 47 | Male | III | NA | Mutant | WT | Regional Lymph Node | Metastatic | NA | NA | NO | NO |
| **TCGA-EE-A3AC-06** | 47 | Male | III | NA | WT | Mutant | Regional Lymph Node | Metastatic | NA | NO | NO | NO |
| **TCGA-ER-A19N-06** | 47 | Male | NA | NA | WT | Mutant | Regional Lymph Node | Metastatic | NA | YES | YES | NO |
| **TCGA-ER-A19P-06** | 47 | Female | NA | NA | WT | WT | Regional Lymph Node | Metastatic | NA | NA | NO | NO |
| **TCGA-GN-A262-06** | 47 | Female | NA | 3 | Mutant | WT | Regional Cutaneous or Subcutaneous Tissue | Metastatic | NA | NO | NO | NO |
| **TCGA-WE-AAA0-06** | 47 | Male | I | 0.8 | Mutant | WT | Regional Lymph Node | Metastatic | NO | YES | YES | NO |
| **TCGA-EE-A3AF-06** | 48 | Female | III | NA | Mutant | WT | Regional Lymph Node | Metastatic | NA | NA | NO | YES |
| **TCGA-EE-A3JI-06** | 48 | Male | I | 1.2 | WT | Mutant | Regional Lymph Node | Metastatic | NO | YES | YES | YES |
| **TCGA-ER-A19G-06** | 48 | Female | NA | 1.6 | Mutant | WT | Regional Lymph Node | Metastatic | NO | NO | NO | YES |
| **TCGA-WE-A8ZQ-06** | 48 | Male | II | 2.3 | Mutant | WT | Regional Lymph Node | Metastatic | NO | NO | NO | YES |
| **TCGA-D3-A2J8-06** | 48 | Male | I | 1.4 | WT | WT | Regional Lymph Node | Metastatic | NO | NA | NO | NO |
| **TCGA-D3-A8GB-06** | 48 | Male | III | 3.5 | Mutant | WT | Regional Lymph Node | Metastatic | NO | NA | YES | NO |
| **TCGA-D3-A8GC-06** | 48 | Male | III | NA | WT | Mutant | Regional Lymph Node | Metastatic | NA | NA | NO | NO |
| **TCGA-EE-A183-06** | 48 | Male | 0 | NA | Mutant | WT | Regional Lymph Node | Metastatic | NA | NA | NO | NO |
| **TCGA-EE-A29R-06** | 48 | Female | III | 3.5 | WT | Mutant | Regional Lymph Node | Metastatic | YES | NA | NO | NO |
| **TCGA-ER-A19W-06** | 48 | Female | NA | 0.75 | WT | WT | Regional Cutaneous or Subcutaneous Tissue | Metastatic | NO | NA | NO | NO |
| **TCGA-FW-A5DY-06** | 48 | Female | III | NA | WT | WT | Regional Lymph Node | Metastatic | NA | NO | NO | NO |
| **TCGA-EE-A2M5-06** | 49 | Male | I | 0.8 | Mutant | WT | Regional Lymph Node | Metastatic | NO | NA | NO | YES |
| **TCGA-WE-A8ZR-06** | 49 | Male | III | 7.3 | Mutant | WT | Regional Lymph Node | Metastatic | YES | YES | NO | YES |
| **TCGA-EB-A5UN-06** | 49 | Male | II | 14 | Mutant | WT | Distant Metastasis | Metastatic | YES | NO | NO | NO |
| **TCGA-ER-A2NH-06** | 49 | Male | III | 4 | WT | Mutant | Regional Lymph Node | Metastatic | NO | NO | NO | NO |
| **TCGA-ER-A42L-06** | 49 | Male | II | 1.46 | WT | WT | Distant Metastasis | Metastatic | NO | NO | NO | NO |
| **TCGA-OD-A75X-06** | 49 | Male | NA | NA | Mutant | WT | Distant Metastasis | Metastatic | NA | NO | NO | NO |
| **TCGA-QB-A6FS-06** | 49 | Male | III | NA | Mutant | WT | Regional Lymph Node | Metastatic | NA | NO | NO | NO |
| **TCGA-ER-A2NC-06** | 50 | Male | I | 1.97 | Mutant | Mutant | Distant Metastasis | Metastatic | NO | NA | NO | YES |
| **TCGA-D3-A2JO-06** | 50 | Female | III | NA | WT | Mutant | Regional Cutaneous or Subcutaneous Tissue | Metastatic | YES | YES | NO | NO |
| **TCGA-EE-A3AD-06** | 50 | Male | III | NA | Mutant | WT | Regional Lymph Node | Metastatic | NA | NO | NO | NO |
| **TCGA-FR-A7U8-06** | 50 | Male | III | NA | WT | Mutant | Distant Metastasis | Metastatic | NA | NO | YES | NO |
| **TCGA-HR-A2OG-06** | 50 | Female | NA | NA | WT | Mutant | Distant Metastasis | Metastatic | NA | NA | NO | NO |
| **TCGA-ER-A19T-06** | 51 | Male | IV | 15 | WT | WT | Primary Tumor | Metastatic | NO | YES | NO | NO |
| **TCGA-D3-A51F-06** | 51 | Male | III | 7 | WT | WT | Regional Lymph Node | Metastatic | YES | NO | NO | NO |
| **TCGA-D3-A51K-06** | 51 | Male | III | 0 | WT | WT | Regional Lymph Node | Metastatic | NA | NO | NO | NO |
| **TCGA-DA-A1I4-06** | 51 | Male | III | 3.4 | Mutant | WT | Regional Cutaneous or Subcutaneous Tissue | Metastatic | YES | NO | NO | NO |
| **TCGA-EB-A44Q-06** | 51 | Female | III | NA | WT | Mutant | Regional Lymph Node | Metastatic | YES | NO | NO | NO |
| **TCGA-EE-A29T-06** | 51 | Female | NA | NA | Mutant | WT | Regional Lymph Node | Metastatic | NA | NO | NO | NO |
| **TCGA-EE-A2GB-06** | 51 | Male | III | 1.65 | Mutant | WT | Regional Lymph Node | Metastatic | YES | YES | NO | NO |
| **TCGA-EE-A2ME-06** | 51 | Male | I | 0.52 | WT | WT | Regional Lymph Node | Metastatic | NO | NA | NO | NO |
| **TCGA-FS-A1ZC-06** | 51 | Male | I/II | NA | Mutant | WT | Distant Metastasis | Metastatic | NA | NA | NO | NO |
| **TCGA-GN-A4U8-06** | 51 | Male | NA | NA | Mutant | WT | Regional Lymph Node | Metastatic | NA | YES | YES | NO |
| **TCGA-EE-A2MD-06** | 52 | Male | II | 3 | WT | Mutant | Regional Lymph Node | Metastatic | YES | NA | NO | YES |
| **TCGA-D3-A8GS-06** | 52 | Male | I | 0.6 | Mutant | WT | Distant Metastasis | Metastatic | NA | NA | NO | NO |
| **TCGA-DA-A3F3-06** | 52 | Male | III | NA | WT | Mutant | Regional Lymph Node | Metastatic | NA | NO | NO | NO |
| **TCGA-DA-A95W-06** | 52 | Male | III | NA | Mutant | WT | Regional Lymph Node | Metastatic | NA | NO | NO | NO |
| **TCGA-EB-A44R-06** | 52 | Male | III | NA | WT | Mutant | Regional Lymph Node | Metastatic | NA | NO | NO | NO |
| **TCGA-EE-A3AE-06** | 52 | Female | I | 0.9 | WT | WT | Regional Lymph Node | Metastatic | NO | NO | NO | NO |
| **TCGA-FS-A1YW-06** | 52 | Male | I | 1 | WT | Mutant | Regional Lymph Node | Metastatic | NO | NA | NO | NO |
| **TCGA-FS-A1ZP-06** | 52 | Male | II | 2.5 | WT | Mutant | Regional Lymph Node | Metastatic | NA | NA | NO | NO |
| **TCGA-FS-A4F8-06** | 52 | Male | I | 0.9 | Mutant | WT | Regional Lymph Node | Metastatic | NA | NA | NO | NO |
| **TCGA-ER-A2NF-06** | 53 | Male | III | 4 | WT | WT | Primary Tumor | Metastatic | YES | NO | YES | YES |
| **TCGA-D3-A1Q4-06** | 53 | Female | III | 1.45 | Mutant | WT | Regional Lymph Node | Metastatic | YES | NO | NO | NO |
| **TCGA-D3-A2JC-06** | 53 | Female | III | NA | WT | WT | Regional Lymph Node | Metastatic | NA | NO | YES | NO |
| **TCGA-D3-A3MU-06** | 53 | Male | III | 2.9 | Mutant | WT | Regional Lymph Node | Metastatic | NO | NO | NO | NO |
| **TCGA-EE-A20F-06** | 53 | Male | I | 0.5 | Mutant | WT | Regional Lymph Node | Metastatic | NA | YES | NO | NO |
| **TCGA-EE-A29G-06** | 53 | Male | III | 7.4 | WT | WT | Regional Cutaneous or Subcutaneous Tissue | Metastatic | NO | NA | NO | NO |
| **TCGA-GN-A265-06** | 53 | Male | NA | NA | Mutant | WT | Regional Lymph Node | Metastatic | NA | NO | NO | NO |
| **TCGA-ER-A19J-06** | 54 | Male | IV | NA | Mutant | WT | Regional Lymph Node | Metastatic | NA | NO | NO | YES |
| **TCGA-D3-A3C6-06** | 54 | Female | I | 1.3 | Mutant | WT | Regional Lymph Node | Metastatic | NO | NA | NO | NO |
| **TCGA-D3-A8GR-06** | 54 | Female | 0 | 0.01 | WT | Mutant | Distant Metastasis | Metastatic | NA | NA | NO | NO |
| **TCGA-D9-A4Z6-06** | 54 | Male | III | NA | Mutant | WT | Regional Lymph Node | Metastatic | NA | NO | NO | NO |
| **TCGA-EE-A17X-06** | 54 | Male | I | 0.8 | WT | Mutant | Regional Lymph Node | Metastatic | NO | NA | NO | NO |
| **TCGA-EE-A29E-06** | 54 | Male | III | 3.2 | Mutant | WT | Regional Lymph Node | Metastatic | NO | NO | NO | NO |
| **TCGA-EE-A2M8-06** | 54 | Female | III | 2 | WT | WT | Regional Lymph Node | Metastatic | NO | NA | NO | NO |
| **TCGA-EE-A3JH-06** | 54 | Male | I | 1.3 | WT | WT | Regional Lymph Node | Metastatic | NA | NO | NO | NO |
| **TCGA-FS-A1ZS-06** | 54 | Male | I | 1.15 | Mutant | WT | Regional Lymph Node | Metastatic | NA | NO | YES | NO |
| **TCGA-FS-A1ZZ-06** | 54 | Female | II | 3.8 | Mutant | Mutant | Distant Metastasis | Metastatic | YES | NA | NO | NO |
| **TCGA-XV-AB01-06** | 54 | Female | II | NA | NA | NA | Regional Lymph Node | Metastatic | NA | NO | YES | NO |
| **TCGA-D3-A1QA-06** | 55 | Male | I | 1.12 | Mutant | WT | Regional Lymph Node | Metastatic | NO | NO | NO | NO |
| **TCGA-EB-A5KH-06** | 55 | Male | III | NA | WT | WT | Regional Lymph Node | Metastatic | NA | NO | YES | YES |
| **TCGA-EE-A185-06** | 55 | Female | III | 6 | WT | Mutant | Regional Lymph Node | Metastatic | YES | NA | NO | YES |
| **TCGA-D3-A1Q6-06** | 55 | Male | III | 50 | Mutant | WT | Regional Cutaneous or Subcutaneous Tissue | Metastatic | NA | NA | YES | NO |
| **TCGA-DA-A1I1-06** | 55 | Male | III | NA | WT | Mutant | Regional Cutaneous or Subcutaneous Tissue | Metastatic | NA | NO | YES | NO |
| **TCGA-DA-A3F2-06** | 55 | Male | III | 5.1 | WT | WT | Regional Cutaneous or Subcutaneous Tissue | Metastatic | NO | NO | YES | NO |
| **TCGA-EB-A6L9-06** | 55 | Male | III | NA | WT | WT | Regional Lymph Node | Metastatic | YES | NO | NO | NO |
| **TCGA-ER-A3EV-06** | 55 | Male | III | 4.4 | WT | WT | Distant Metastasis | Metastatic | NA | NO | NO | NO |
| **TCGA-FS-A1YY-06** | 55 | Female | II | 2.7 | Mutant | WT | Distant Metastasis | Metastatic | NO | NA | NO | NO |
| **TCGA-FS-A1ZT-06** | 55 | Male | III | 1.15 | WT | Mutant | Distant Metastasis | Metastatic | NA | NO | YES | NO |
| **TCGA-GN-A4U7-06** | 56 | Female | III | 1.39 | Mutant | WT | Regional Lymph Node | Metastatic | NO | NO | NO | YES |
| **TCGA-WE-AAA4-06** | 56 | Female | III | NA | Mutant | WT | Regional Cutaneous or Subcutaneous Tissue | Metastatic | NA | NO | YES | YES |
| **TCGA-D3-A51N-06** | 56 | Female | IV | NA | Mutant | WT | Regional Lymph Node | Metastatic | NA | NO | NO | NO |
| **TCGA-D9-A6EC-06** | 56 | Male | III | 3 | Mutant | WT | Regional Lymph Node | Metastatic | NO | NO | NO | NO |
| **TCGA-D9-A6EG-06** | 56 | Male | III | 5 | Mutant | WT | Regional Cutaneous or Subcutaneous Tissue | Metastatic | YES | NO | NO | NO |
| **TCGA-EE-A20H-06** | 56 | Male | I | 1.05 | Mutant | WT | Distant Metastasis | Metastatic | NA | NA | NO | NO |
| **TCGA-FR-A8YD-06** | 56 | Female | II | 4.5 | WT | WT | Distant Metastasis | Metastatic | YES | NA | YES | NO |
| **TCGA-W3-AA1Q-06** | 57 | Male | III | NA | Mutant | WT | Distant Metastasis | Metastatic | NA | NA | YES | YES |
| **TCGA-WE-A8ZN-06** | 57 | Male | II | 4.5 | WT | Mutant | Regional Cutaneous or Subcutaneous Tissue | Metastatic | NO | NO | NO | YES |
| **TCGA-D3-A3C7-06** | 57 | Female | III | NA | Mutant | WT | Regional Lymph Node | Metastatic | NA | NO | YES | NO |
| **TCGA-EB-A5SG-06** | 57 | Female | NA | NA | WT | WT | Regional Lymph Node | Metastatic | NO | NO | NO | NO |
| **TCGA-EE-A17Z-06** | 57 | Male | II | 29 | WT | WT | Regional Lymph Node | Metastatic | NO | NA | NO | NO |
| **TCGA-ER-A2ND-06** | 57 | Female | III | 1 | Mutant | WT | Distant Metastasis | Metastatic | NO | NA | NO | NO |
| **TCGA-FS-A1ZB-06** | 57 | Male | II | 3 | WT | Mutant | Distant Metastasis | Metastatic | NO | NA | NO | NO |
| **TCGA-FW-A3TV-06** | 57 | Female | III | 0.5 | Mutant | WT | Regional Lymph Node | Metastatic | NA | NO | YES | NO |
| **TCGA-Z2-AA3V-06** | 57 | Female | I | 0.5 | Mutant | WT | Regional Lymph Node | Metastatic | NO | NO | NO | NO |
| **TCGA-EE-A29X-06** | 58 | Female | I | 2 | WT | Mutant | Regional Lymph Node | Metastatic | NO | NA | NO | YES |
| **TCGA-EE-A2MN-06** | 58 | Male | I | 1 | WT | Mutant | Regional Lymph Node | Metastatic | NO | NA | NO | YES |
| **TCGA-GF-A3OT-06** | 58 | Female | III | NA | WT | Mutant | Regional Lymph Node | Metastatic | NA | NA | YES | YES |
| **TCGA-D3-A2JD-06** | 58 | Male | III | 18 | Mutant | WT | Regional Lymph Node | Metastatic | YES | NA | NO | NO |
| **TCGA-D3-A3C8-06** | 58 | Female | III | NA | WT | WT | Regional Cutaneous or Subcutaneous Tissue | Metastatic | NA | YES | NO | NO |
| **TCGA-D3-A5GS-06** | 58 | Male | IV | 0.62 | Mutant | WT | Regional Lymph Node | Metastatic | NO | NO | NO | NO |
| **TCGA-EE-A2GD-06** | 58 | Female | II | 4.4 | WT | Mutant | Regional Cutaneous or Subcutaneous Tissue | Metastatic | YES | NO | NO | NO |
| **TCGA-ER-A1A1-06** | 58 | Male | III | NA | WT | WT | Regional Lymph Node | Metastatic | NA | YES | YES | NO |
| **TCGA-Z2-AA3S-06** | 58 | Male | I | 0.5 | WT | WT | Regional Lymph Node | Metastatic | NO | YES | NO | NO |
| **TCGA-GN-A9SD-06** | 59 | Female | I | 0.5 | Mutant | WT | Regional Lymph Node | Metastatic | NO | NA | NO | YES |
| **TCGA-D3-A51T-06** | 59 | Female | III | 4.2 | Mutant | WT | Regional Lymph Node | Metastatic | YES | YES | NO | NO |
| **TCGA-EE-A20C-06** | 59 | Male | 0 | NA | WT | Mutant | Distant Metastasis | Metastatic | NA | NA | NO | NO |
| **TCGA-EE-A29H-06** | 59 | Female | I | 1 | Mutant | WT | Regional Lymph Node | Metastatic | NO | NO | NO | NO |
| **TCGA-EE-A3J8-06** | 59 | Male | III | 4.8 | WT | Mutant | Regional Cutaneous or Subcutaneous Tissue | Metastatic | NO | NA | NO | NO |
| **TCGA-FW-A3I3-06** | 59 | Female | IV | NA | WT | WT | Distant Metastasis | Metastatic | NA | NO | NO | NO |
| **TCGA-D3-A51H-06** | 60 | Male | III | 0.91 | WT | WT | Regional Lymph Node | Metastatic | YES | NO | NO | YES |
| **TCGA-EB-A5SH-06** | 60 | Female | III | 5 | WT | WT | Regional Lymph Node | Metastatic | YES | NO | YES | YES |
| **TCGA-EE-A3JB-06** | 60 | Female | III | 1.86 | Mutant | WT | Regional Cutaneous or Subcutaneous Tissue | Metastatic | NA | NA | NO | YES |
| **TCGA-W3-A825-06** | 60 | Female | II | 1.3 | Mutant | Mutant | Distant Metastasis | Metastatic | NA | NA | YES | YES |
| **TCGA-D3-A1Q5-06** | 60 | Male | I/II | NA | Mutant | WT | Regional Lymph Node | Metastatic | NA | NA | NO | NO |
| **TCGA-D3-A51R-06** | 60 | Male | II | 2.5 | WT | Mutant | Regional Lymph Node | Metastatic | NO | NO | NO | NO |
| **TCGA-EE-A2MJ-06** | 60 | Male | III | 11 | WT | WT | Regional Lymph Node | Metastatic | NO | NA | NO | NO |
| **TCGA-FS-A1ZG-06** | 60 | Female | III | 6 | WT | WT | Regional Lymph Node | Metastatic | YES | NA | NO | NO |
| **TCGA-GN-A264-06** | 60 | Male | NA | 7 | WT | WT | Regional Lymph Node | Metastatic | NO | NA | YES | NO |
| **TCGA-D3-A3CF-06** | 61 | Female | III | 9 | WT | WT | Regional Lymph Node | Metastatic | YES | NA | NO | NO |
| **TCGA-D3-A5GO-06** | 61 | Male | II | 4.3 | WT | Mutant | Distant Metastasis | Metastatic | NA | NA | NO | NO |
| **TCGA-EE-A2M6-06** | 61 | Male | I | 0.7 | Mutant | WT | Regional Lymph Node | Metastatic | NO | NA | NO | NO |
| **TCGA-EE-A2MR-06** | 61 | Male | I | 1.25 | WT | WT | Regional Lymph Node | Metastatic | NO | YES | NO | NO |
| **TCGA-DA-A1I7-06** | 62 | Male | III | NA | Mutant | WT | Regional Cutaneous or Subcutaneous Tissue | Metastatic | NA | NO | NO | NO |
| **TCGA-DA-A95X-06** | 62 | Male | I | 1.45 | WT | Mutant | Regional Lymph Node | Metastatic | NO | NO | NO | NO |
| **TCGA-ER-A193-06** | 62 | Male | II | NA | Mutant | WT | Regional Lymph Node | Metastatic | NO | NA | NO | NO |
| **TCGA-FS-A1Z4-06** | 62 | Male | I | 0.85 | WT | WT | Regional Lymph Node | Metastatic | NO | NA | NO | NO |
| **TCGA-GF-A6C8-06** | 62 | Female | II | 4 | Mutant | WT | Regional Lymph Node | Metastatic | YES | NA | NO | NO |
| **TCGA-WE-A8ZY-06** | 62 | Male | II | 3 | WT | Mutant | Regional Cutaneous or Subcutaneous Tissue | Metastatic | NO | NO | NO | NO |
| **TCGA-YG-AA3O-06** | 62 | Male | NA | NA | Mutant | WT | Regional Lymph Node | Metastatic | NA | NO | YES | NO |
| **TCGA-D3-A3BZ-06** | 63 | Male | II | 4.6 | WT | WT | Regional Lymph Node | Metastatic | NO | YES | NO | NO |
| **TCGA-D3-A8GD-06** | 63 | Female | III | 20 | Mutant | WT | Regional Lymph Node | Metastatic | YES | NO | NO | NO |
| **TCGA-D9-A1X3-06** | 63 | Male | NA | 12 | WT | WT | Regional Cutaneous or Subcutaneous Tissue | Metastatic | YES | YES | NO | NO |
| **TCGA-DA-A1I0-06** | 63 | Male | IV | 4.5 | Mutant | WT | Regional Cutaneous or Subcutaneous Tissue | Metastatic | YES | NO | NO | NO |
| **TCGA-DA-A1I8-06** | 63 | Female | II | 8 | Mutant | WT | Regional Cutaneous or Subcutaneous Tissue | Metastatic | YES | NO | NO | NO |
| **TCGA-EE-A2MM-06** | 63 | Female | I | 0.6 | WT | Mutant | Regional Lymph Node | Metastatic | NA | NA | NO | NO |
| **TCGA-FR-A7U9-06** | 63 | Female | III | 2.8 | WT | WT | Regional Lymph Node | Metastatic | YES | NO | YES | NO |
| **TCGA-FS-A1ZD-06** | 63 | Male | II | 1.18 | Mutant | WT | Regional Lymph Node | Metastatic | YES | NA | NO | NO |
| **TCGA-GN-A26A-06** | 63 | Female | III | 2.3 | WT | WT | Regional Cutaneous or Subcutaneous Tissue | Metastatic | NO | NA | NO | NO |
| **TCGA-W3-A824-06** | 63 | Male | I | 1.5 | WT | Mutant | Regional Lymph Node | Metastatic | NA | NA | YES | NO |
| **TCGA-YG-AA3P-06** | 63 | Female | II | 10 | WT | WT | Distant Metastasis | Metastatic | NO | NO | YES | NO |
| **TCGA-D3-A1Q3-06** | 64 | Male | II | 4.6 | WT | WT | Regional Lymph Node | Metastatic | YES | NA | NO | YES |
| **TCGA-ER-A3ET-06** | 64 | Female | III | 3.55 | WT | WT | Regional Cutaneous or Subcutaneous Tissue | Metastatic | NA | YES | NO | NO |
| **TCGA-FS-A4F4-06** | 64 | Male | II | 2 | WT | WT | Regional Cutaneous or Subcutaneous Tissue | Metastatic | YES | NA | NO | NO |
| **TCGA-W3-AA1W-06** | 64 | Male | II | 1.54 | Mutant | WT | Distant Metastasis | Metastatic | NO | YES | YES | NO |
| **TCGA-WE-A8K5-06** | 65 | Male | IV | 1.7 | Mutant | WT | Regional Cutaneous or Subcutaneous Tissue | Metastatic | NO | NO | NO | YES |
| **TCGA-D3-A2J6-06** | 65 | Male | II | 3.2 | WT | WT | Regional Lymph Node | Metastatic | YES | NA | NO | NO |
| **TCGA-D9-A149-06** | 65 | Female | NA | NA | WT | WT | Regional Cutaneous or Subcutaneous Tissue | Metastatic | NO | NO | NO | NO |
| **TCGA-EE-A2GU-06** | 65 | Female | I | 0.3 | WT | Mutant | Regional Lymph Node | Metastatic | NO | NO | NO | NO |
| **TCGA-FR-A7UA-06** | 65 | Female | I | 1.02 | Mutant | WT | Regional Lymph Node | Metastatic | NO | YES | NO | NO |
| **TCGA-FS-A1ZW-06** | 65 | Male | III | 1.2 | WT | Mutant | Regional Lymph Node | Metastatic | YES | NO | YES | NO |
| **TCGA-D3-A8GQ-06** | 66 | Male | II | 2.6 | WT | Mutant | Distant Metastasis | Metastatic | NA | NA | NO | NO |
| **TCGA-D9-A3Z1-06** | 66 | Male | III | 1.7 | WT | Mutant | Regional Lymph Node | Metastatic | NA | NO | NO | NO |
| **TCGA-EE-A20B-06** | 66 | Female | II | 2.6 | Mutant | WT | Regional Cutaneous or Subcutaneous Tissue | Metastatic | NA | NO | NO | NO |
| **TCGA-EE-A2GO-06** | 66 | Female | II | 3.4 | WT | WT | Regional Cutaneous or Subcutaneous Tissue | Metastatic | NO | NO | NO | NO |
| **TCGA-EE-A2M7-06** | 66 | Male | II | 2.2 | Mutant | WT | Regional Lymph Node | Metastatic | NA | NA | NO | NO |
| **TCGA-EE-A2MH-06** | 66 | Male | III | 3.4 | Mutant | WT | Regional Lymph Node | Metastatic | NO | NA | NO | NO |
| **TCGA-RP-A690-06** | 66 | Female | NA | NA | WT | WT | Distant Metastasis | Metastatic | NA | NA | NO | NO |
| **TCGA-W3-A828-06** | 66 | Male | II | 0.5 | WT | WT | Distant Metastasis | Metastatic | NA | NA | YES | NO |
| **TCGA-FS-A4F0-06** | 67 | Female | II | 14 | WT | WT | Regional Cutaneous or Subcutaneous Tissue | Metastatic | NO | NO | YES | YES |
| **TCGA-D3-A2J7-06** | 67 | Male | III | 3.6 | Mutant | WT | Regional Lymph Node | Metastatic | YES | NA | NO | NO |
| **TCGA-EE-A29B-06** | 67 | Male | II | 4 | WT | WT | Distant Metastasis | Metastatic | YES | NO | NO | NO |
| **TCGA-EE-A2GN-06** | 67 | Male | II | 1.9 | WT | Mutant | Regional Cutaneous or Subcutaneous Tissue | Metastatic | YES | NO | NO | NO |
| **TCGA-D3-A2JA-06** | 68 | Male | III | 1.2 | Mutant | WT | Regional Cutaneous or Subcutaneous Tissue | Metastatic | NO | NO | NO | NO |
| **TCGA-D3-A2JH-06** | 68 | Male | I | 1 | Mutant | WT | Regional Cutaneous or Subcutaneous Tissue | Metastatic | NO | YES | YES | NO |
| **TCGA-D3-A8GI-06** | 68 | Male | I | 0.98 | WT | Mutant | Regional Lymph Node | Metastatic | NO | NA | NO | NO |
| **TCGA-DA-A95Y-06** | 68 | Male | II | 5.03 | WT | WT | Regional Lymph Node | Metastatic | YES | NO | NO | NO |
| **TCGA-EE-A29A-06** | 68 | Male | III | 2.3 | Mutant | WT | Distant Metastasis | Metastatic | NO | NA | NO | NO |
| **TCGA-FS-A1ZK-06** | 68 | Male | II | 5 | WT | WT | Distant Metastasis | Metastatic | NA | NA | NO | NO |
| **TCGA-FW-A3R5-06** | 68 | Male | III | NA | Mutant | WT | Regional Lymph Node | Metastatic | NA | NO | YES | NO |
| **TCGA-GN-A8LL-06** | 68 | Female | II | 5 | WT | WT | Regional Lymph Node | Metastatic | YES | NA | NO | NO |
| **TCGA-D3-A3CC-06** | 69 | Female | II | 7.2 | WT | WT | Regional Cutaneous or Subcutaneous Tissue | Metastatic | YES | NO | NO | NO |
| **TCGA-DA-A1IB-06** | 69 | Female | III | 1.5 | WT | WT | Regional Lymph Node | Metastatic | YES | NO | NO | NO |
| **TCGA-EE-A17Y-06** | 69 | Male | III | 2.8 | Mutant | WT | Regional Cutaneous or Subcutaneous Tissue | Metastatic | YES | NA | NO | NO |
| **TCGA-EE-A180-06** | 69 | Male | III | 4.9 | WT | WT | Distant Metastasis | Metastatic | NO | NA | NO | NO |
| **TCGA-EE-A29Q-06** | 70 | Female | II | 2.3 | WT | Mutant | Regional Cutaneous or Subcutaneous Tissue | Metastatic | YES | NO | NO | YES |
| **TCGA-EE-A2MQ-06** | 70 | Female | III | 3.1 | Mutant | WT | Regional Lymph Node | Metastatic | NO | NA | NO | YES |
| **TCGA-GN-A8LK-06** | 70 | Male | I | 0.7 | WT | WT | Distant Metastasis | Metastatic | NO | NA | NO | YES |
| **TCGA-D3-A2JB-06** | 70 | Female | 0 | NA | WT | WT | Regional Lymph Node | Metastatic | NA | NA | NO | NO |
| **TCGA-D3-A3ML-06** | 70 | Male | III | 2.3 | WT | Mutant | Regional Lymph Node | Metastatic | NO | NA | NO | NO |
| **TCGA-D9-A6EA-06** | 70 | Male | III | 6 | WT | Mutant | NA | Metastatic | NO | NO | NO | NO |
| **TCGA-EE-A3JD-06** | 70 | Male | III | NA | WT | WT | Regional Lymph Node | Metastatic | NA | NA | NO | NO |
| **TCGA-FS-A1ZU-06** | 70 | Female | II | 8.4 | WT | WT | Distant Metastasis | Metastatic | YES | NA | NO | NO |
| **TCGA-WE-A8JZ-06** | 70 | Male | III | 14 | WT | Mutant | Regional Lymph Node | Metastatic | YES | NA | NO | NO |
| **TCGA-WE-A8ZM-06** | 70 | Male | III | NA | WT | WT | Regional Cutaneous or Subcutaneous Tissue | Metastatic | NA | YES | YES | NO |
| **TCGA-EB-A5UL-06** | 71 | Male | III | 4 | WT | Mutant | Regional Lymph Node | Metastatic | NA | NO | NO | YES |
| **TCGA-GN-A4U9-06** | 71 | Male | III | 1.7 | WT | Mutant | Regional Lymph Node | Metastatic | YES | NO | YES | YES |
| **TCGA-3N-A9WB-06** | 71 | Male | I | 0.7 | Mutant | Mutant | Distant Metastasis | Metastatic | NO | NA | NO | NO |
| **TCGA-EE-A2A2-06** | 71 | Male | III | 7 | WT | Mutant | Regional Lymph Node | Metastatic | YES | NO | NO | NO |
| **TCGA-EE-A2MU-06** | 71 | Male | I | 0.5 | WT | Mutant | Regional Cutaneous or Subcutaneous Tissue | Metastatic | NO | NA | NO | NO |
| **TCGA-EE-A3J5-06** | 71 | Male | III | 4.8 | WT | WT | Regional Cutaneous or Subcutaneous Tissue | Metastatic | NO | NA | NO | NO |
| **TCGA-FS-A1ZH-06** | 71 | Female | IV | 3 | Mutant | WT | Regional Lymph Node | Metastatic | YES | NA | NO | NO |
| **TCGA-FS-A1ZY-06** | 71 | Male | II | 2.9 | WT | Mutant | Distant Metastasis | Metastatic | YES | NA | NO | NO |
| **TCGA-RP-A694-06** | 71 | Male | IV | NA | WT | Mutant | Distant Metastasis | Metastatic | NA | NA | NO | NO |
| **TCGA-W3-AA1R-06** | 71 | Male | II | NA | Mutant | WT | Regional Lymph Node | Metastatic | NA | NA | YES | NO |
| **TCGA-D3-A1Q9-06** | 72 | Male | III | 6 | Mutant | WT | Regional Lymph Node | Metastatic | YES | NA | NO | NO |
| **TCGA-EE-A184-06** | 72 | Male | I | 1.95 | WT | Mutant | Distant Metastasis | Metastatic | NO | NA | NO | NO |
| **TCGA-EE-A2MS-06** | 72 | Male | II | 1.8 | WT | WT | Regional Lymph Node | Metastatic | NO | NO | NO | NO |
| **TCGA-EE-A3J4-06** | 72 | Male | II | 2 | Mutant | WT | Regional Cutaneous or Subcutaneous Tissue | Metastatic | YES | NA | NO | NO |
| **TCGA-FS-A1Z3-06** | 72 | Female | IV | NA | WT | Mutant | Distant Metastasis | Metastatic | NA | NA | NO | NO |
| **TCGA-FW-A3TU-06** | 72 | Female | NA | NA | WT | WT | Distant Metastasis | Metastatic | NA | NA | NO | NO |
| **TCGA-GN-A26D-06** | 72 | Female | II | 12 | WT | WT | Regional Lymph Node | Metastatic | YES | NO | NO | NO |
| **TCGA-GN-A4U4-06** | 73 | Male | II | 1.7 | WT | Mutant | Regional Lymph Node | Metastatic | YES | NO | YES | YES |
| **TCGA-D3-A8GM-06** | 73 | Male | II | 3.8 | WT | WT | Regional Lymph Node | Metastatic | YES | YES | NO | NO |
| **TCGA-EE-A29P-06** | 73 | Female | II | 4.5 | Mutant | WT | Regional Lymph Node | Metastatic | YES | NA | NO | NO |
| **TCGA-EE-A2MC-06** | 73 | Male | I | 1 | WT | Mutant | Regional Lymph Node | Metastatic | NO | NA | NO | NO |
| **TCGA-QB-AA9O-06** | 73 | Male | III | NA | WT | Mutant | Regional Lymph Node | Metastatic | NA | NA | YES | NO |
| **TCGA-WE-A8ZO-06** | 73 | Female | III | 2.4 | WT | Mutant | Regional Lymph Node | Metastatic | NO | NO | NO | NO |
| **TCGA-D3-A2JF-06** | 74 | Male | I | 0.28 | WT | WT | Distant Metastasis | Metastatic | NO | NO | NO | NO |
| **TCGA-D3-A3CE-06** | 74 | Female | III | NA | Mutant | WT | Regional Lymph Node | Metastatic | NA | NA | NO | NO |
| **TCGA-D3-A5GL-06** | 74 | Male | I | 1.6 | WT | WT | Regional Lymph Node | Metastatic | NO | YES | NO | NO |
| **TCGA-EB-A5VV-06** | 74 | Female | III | 4 | WT | WT | Regional Lymph Node | Metastatic | YES | NO | NO | NO |
| **TCGA-FS-A1ZM-06** | 74 | Male | III | 1.2 | Mutant | WT | Regional Cutaneous or Subcutaneous Tissue | Metastatic | NA | NA | NO | NO |
| **TCGA-GF-A4EO-06** | 74 | Female | III | NA | Mutant | WT | Regional Lymph Node | Metastatic | NA | YES | NO | NO |
| **TCGA-WE-A8K1-06** | 74 | Male | III | 3 | WT | Mutant | Regional Cutaneous or Subcutaneous Tissue | Metastatic | YES | NO | NO | NO |
| **TCGA-D3-A1QB-06** | 75 | Female | III | NA | WT | WT | Regional Lymph Node | Metastatic | NA | NO | NO | NO |
| **TCGA-D3-A2J9-06** | 75 | Male | III | 21 | WT | WT | Regional Lymph Node | Metastatic | YES | NA | NO | NO |
| **TCGA-D3-A2JE-06** | 75 | Female | III | NA | Mutant | WT | Regional Lymph Node | Metastatic | NA | NA | NO | NO |
| **TCGA-D9-A6E9-06** | 75 | Female | III | 4 | Mutant | WT | Primary Tumor | Metastatic | NO | NO | NO | NO |
| **TCGA-DA-A1HV-06** | 75 | Female | III | NA | WT | Mutant | Regional Lymph Node | Metastatic | NA | NO | NO | NO |
| **TCGA-EE-A3JE-06** | 75 | Male | III | 3.7 | Mutant | WT | Regional Cutaneous or Subcutaneous Tissue | Metastatic | YES | NO | NO | NO |
| **TCGA-FS-A1ZJ-06** | 75 | Female | I | 1.22 | WT | WT | Regional Lymph Node | Metastatic | NO | NA | NO | NO |
| **TCGA-FS-A4FC-06** | 75 | Female | II | 4 | WT | Mutant | Regional Cutaneous or Subcutaneous Tissue | Metastatic | NO | NO | NO | NO |
| **TCGA-YD-A9TA-06** | 75 | Male | NA | 1.5 | Mutant | WT | Distant Metastasis | Metastatic | NA | NO | NO | NO |
| **TCGA-D3-A8GP-06** | 77 | Male | III | 1.8 | WT | WT | Distant Metastasis | Metastatic | NA | NO | NO | NO |
| **TCGA-EE-A2A0-06** | 77 | Female | II | 2.2 | Mutant | WT | Regional Lymph Node | Metastatic | NO | NA | NO | NO |
| **TCGA-EE-A2GT-06** | 77 | Male | II | 2.2 | Mutant | WT | Regional Lymph Node | Metastatic | NO | NO | NO | NO |
| **TCGA-ER-A19C-06** | 77 | Male | I | 0.75 | Mutant | WT | Regional Lymph Node | Metastatic | NO | NA | NO | NO |
| **TCGA-FS-A4F5-06** | 77 | Female | I | 1.48 | WT | Mutant | Regional Cutaneous or Subcutaneous Tissue | Metastatic | NO | NA | NO | NO |
| **TCGA-RP-A693-06** | 77 | Male | IV | NA | WT | WT | Distant Metastasis | Metastatic | NA | NA | NO | NO |
| **TCGA-FR-A8YC-06** | 78 | Male | II | 2.6 | WT | WT | Distant Metastasis | Metastatic | YES | NA | NO | YES |
| **TCGA-EE-A29L-06** | 78 | Male | III | 7 | WT | Mutant | Regional Lymph Node | Metastatic | YES | NA | NO | NO |
| **TCGA-EE-A29N-06** | 78 | Male | I/II | NA | WT | WT | Regional Lymph Node | Metastatic | NA | NA | NO | NO |
| **TCGA-EE-A2GR-06** | 78 | Male | II | 6.9 | WT | WT | Regional Cutaneous or Subcutaneous Tissue | Metastatic | NA | NO | NO | NO |
| **TCGA-FS-A1ZF-06** | 78 | Female | II | 4.3 | Mutant | WT | Distant Metastasis | Metastatic | YES | NA | NO | NO |
| **TCGA-GF-A6C9-06** | 78 | Male | III | NA | Mutant | WT | Distant Metastasis | Metastatic | NA | YES | NO | NO |
| **TCGA-D3-A1Q1-06** | 79 | Female | III | 0.4 | WT | Mutant | Regional Lymph Node | Metastatic | NO | NA | YES | YES |
| **TCGA-WE-A8K6-06** | 79 | Male | III | NA | Mutant | WT | Regional Lymph Node | Metastatic | NA | NO | NO | YES |
| **TCGA-EE-A20I-06** | 79 | Male | IV | NA | WT | WT | Regional Cutaneous or Subcutaneous Tissue | Metastatic | YES | NA | NO | NO |
| **TCGA-EE-A29S-06** | 79 | Male | II | 2.5 | WT | Mutant | Regional Lymph Node | Metastatic | NO | NO | NO | NO |
| **TCGA-ER-A19A-06** | 79 | Male | IV | NA | WT | WT | Regional Cutaneous or Subcutaneous Tissue | Metastatic | NA | NO | NO | NO |
| **TCGA-FS-A4F9-06** | 80 | Male | III | 10 | WT | Mutant | Regional Lymph Node | Metastatic | YES | NO | NO | YES |
| **TCGA-D9-A1JX-06** | 80 | Female | NA | NA | Mutant | WT | Regional Lymph Node | Metastatic | NO | NO | NO | NO |
| **TCGA-EE-A2GP-06** | 80 | Male | III | 4.2 | WT | Mutant | Regional Cutaneous or Subcutaneous Tissue | Metastatic | YES | NA | NO | NO |
| **TCGA-DA-A1IC-06** | 81 | Male | III | 2.5 | WT | WT | Regional Cutaneous or Subcutaneous Tissue | Metastatic | NO | NO | NO | NO |
| **TCGA-ER-A19S-06** | 81 | Female | NA | NA | Mutant | WT | Regional Lymph Node | Metastatic | NA | NO | NO | NO |
| **TCGA-3N-A9WC-06** | 82 | Male | II | 1.8 | Mutant | Mutant | Regional Lymph Node | Metastatic | YES | NO | NO | NO |
| **TCGA-3N-A9WD-06** | 82 | Male | III | 1.25 | WT | Mutant | Regional Lymph Node | Metastatic | NO | NA | NO | NO |
| **TCGA-D9-A1JW-06** | 82 | Male | NA | NA | Mutant | WT | Regional Lymph Node | Metastatic | NO | NO | NO | NO |
| **TCGA-EE-A181-06** | 82 | Female | II | 2.5 | WT | WT | Regional Lymph Node | Metastatic | NA | NA | NO | NO |
| **TCGA-EE-A2GC-06** | 82 | Male | II | 2.3 | Mutant | WT | Regional Lymph Node | Metastatic | YES | NO | NO | NO |
| **TCGA-ER-A19F-06** | 82 | Male | NA | 6.7 | Mutant | WT | Regional Lymph Node | Metastatic | NA | NA | NO | NO |
| **TCGA-DA-A95V-06** | 83 | Female | II | 4.1 | WT | WT | Regional Lymph Node | Metastatic | YES | NO | NO | NO |
| **TCGA-EE-A2GJ-06** | 83 | Male | I | 0.4 | Mutant | Mutant | Regional Lymph Node | Metastatic | NO | NO | NO | NO |
| **TCGA-ER-A197-06** | 83 | Female | III | 8 | WT | WT | NA | Metastatic | YES | NA | NO | NO |
| **TCGA-GN-A268-06** | 83 | Female | II | 9.8 | WT | Mutant | Regional Lymph Node | Metastatic | NO | NA | NO | NO |
| **TCGA-EE-A182-06** | 84 | Female | III | 14 | WT | WT | Distant Metastasis | Metastatic | YES | NA | NO | NO |
| **TCGA-WE-AAA3-06** | 84 | Female | III | 7 | WT | Mutant | Regional Lymph Node | Metastatic | YES | NO | NO | NO |
| **TCGA-EE-A29V-06** | 85 | Male | III | 3.5 | WT | Mutant | Distant Metastasis | Metastatic | YES | NA | NO | YES |
| **TCGA-W3-AA1O-06** | 85 | Male | III | NA | Mutant | WT | Regional Lymph Node | Metastatic | NA | NA | NO | YES |
| **TCGA-ER-A199-06** | 86 | Female | III | 3 | WT | Mutant | Regional Lymph Node | Metastatic | YES | NA | NO | NO |
| **TCGA-DA-A95Z-06** | 87 | Male | IV | NA | Mutant | WT | Regional Cutaneous or Subcutaneous Tissue | Metastatic | NA | NO | NO | NO |
| **TCGA-EE-A29D-06** | 87 | Male | III | 3.2 | WT | WT | Regional Lymph Node | Metastatic | YES | NA | NO | NO |
